# Synthesis and structure–activity relationship studies of benzimidazole-thioquinoline derivatives as α-glucosidase inhibitors

**DOI:** 10.1038/s41598-023-31080-2

**Published:** 2023-03-16

**Authors:** Sara Moghadam Farid, Milad Noori, Mohammad Nazari Montazer, Minoo Khalili Ghomi, Marjan Mollazadeh, Navid Dastyafteh, Cambyz Irajie, Kamiar Zomorodian, Seyedeh Sara Mirfazli, Somayeh Mojtabavi, Mohammad Ali Faramarzi, Bagher Larijani, Aida Iraji, Mohammad Mahdavi

**Affiliations:** 1 Endocrinology and Metabolism Research Center, Endocrinology and Metabolism Clinical Sciences Institute, Tehran University of Medical Sciences, Tehran, Iran; 2 Department of Medical Biotechnology, School of Advanced Medical Sciences and Technologies, Shiraz University of Medical Sciences, Shiraz, Iran; 3 Department of Medical Mycology and Parasitology, School of Medicine, Shiraz University of Medical Sciences, Shiraz, Iran; 4 Department of Medicinal Chemistry, School of Pharmacy, Iran University of Medical Sciences, Tehran, Iran; 5 Department of Pharmaceutical Biotechnology, Faculty of Pharmacy, Tehran University of Medical Sciences, Tehran, Iran; 6 Stem Cells Technology Research Center, Shiraz University of Medical Sciences, Shiraz, Iran; 7 Central Research Laboratory, Shiraz University of Medical Sciences, Shiraz, Iran

**Keywords:** Biological techniques, Chemical biology

## Abstract

In this article, different s-substituted benzimidazole-thioquinoline derivatives were designed, synthesized, and evaluated for their possible α-glucosidase inhibitory activities. The most active compound in this series, **6j** (X = 4-bromobenzyl) exhibited significant potency with an IC_50_ value of 28.0 ± 0.6 µM compared to acarbose as the positive control with an IC_50_ value of 750.0 µM. The kinetic study showed a competitive inhibition pattern against α-glucosidase for the **6j** derivative. Also, the molecular dynamic simulations were performed to determine key interactions between compounds and the targeted enzyme. The in silico pharmacodynamics and ADMET properties were executed to illustrate the druggability of the novel derivatives. In general, it can be concluded that these derivatives can serve as promising leads to the design of potential α-glucosidase inhibitors.

## Introduction

Diabetes is a metabolic disorder characterized by prolonged high blood sugar levels (hyperglycemia) which are associated with complications such as heart, kidney, and nervous system diseases as well as leg amputation and blindness^1,2^. According to the World Health Organization around 422 million people suffered from diabetes in 2014 and this number is predicted to reach 642 million by 2040^3^. Among different types of diabetes, about 90% of cases are type 2 diabetes (T2D)^4^. Current therapeutic approaches to target T2D include dipeptidyl peptidase-IV (DPP-IV) inhibitors^5^, glucagon-like peptide-1 (GLP-1) agonists^6^, and α-glucosidase inhibitors^7^.

α-glucosidase (EC 3.2.1.20) is a key carbohydrate hydrolase enzyme that regulates blood glucose levels by hydrolyzing 1,4-α-glucopyranosidic of oligosaccharide and disaccharide to produce monosaccharides and as a result, the level of glucose in the body increase^8,9^. The primary structure of lysosomal a-glucosidase has 952 amino acids with an apparent molecular mass of 110 kDa. Based on the sequence similarity and the mechanism of binding, Trp-516 and Asp-518 are demonstrated to be critical for catalytic functions^10^. It was shown that inhibition of α-glucosidase decreases carbohydrate digestion and glucose absorption, therefore, stabilizing blood glucose levels and preventing hyperglycemia^7^. Acarbose, (the first approved inhibitor), voglibose (discontinue), and miglitol (the first pseudo-monosaccharide inhibitor), were approved drugs as α-glucosidase inhibitors which reduce postprandial glucose^11^. However, low efficiency and unexpected adverse effects such as flatulence, diarrhea, and stomachache limited their clinical application. As a result, numerous efforts have been carried out to find and develop new a-glucosidase inhibitors from diverse sources, such as natural products and chemical synthetic compounds^12^.

Heterocycle-based α-glucosidase inhibitors have gained attention in the last few years including benzofuran^13^, xanthones^14^, imidazole^15^, benzothiazole^16^, isatin^17^, imidazopyridines^18^ triazole^19^ as well benzimidazole^20,21^ and quinolone.

**Figure 1:**
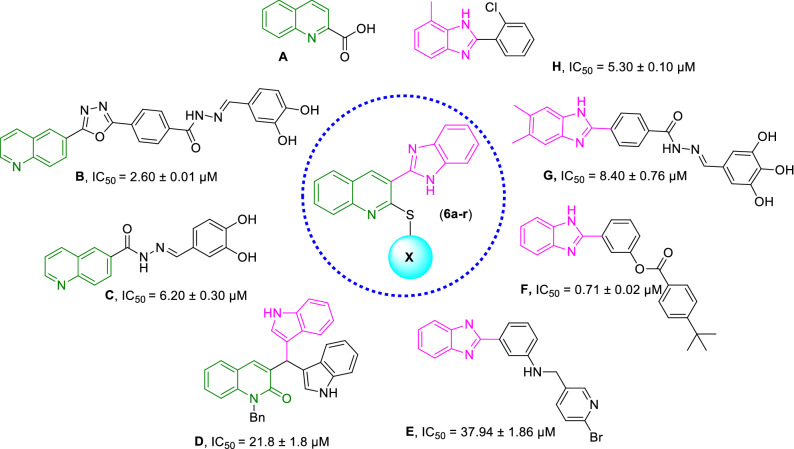
Design of novel α-glucosidase inhibitors (**6a–r**).

Quinoline has been proven to be a very effective pharmacophore as α-glucosidase inhibitors capable of providing hits or leads with easy synthetic protocol and structural diversity which makes ideal structure in anti-diabetic drug discovery. Quinoline-2-carboxylic acid (Compound **A,** Fig. 1) framework showed IC_50_ values of 9.1 ± 2.3 µg/mL^22^. Furthermore, substituted quinolines were reported to possess anti-α-glucosidase inhibition effects. By way of illustration, oxadiazole-quinoline (Compound **B**) has shown potent α-glucosidase inhibition activity (IC_50_ = 2.60 to 102.12 μM) concerning that of the standard acarbose (IC_50_ = 38.25 ± 0.12 μM)^23^, In 2019 anther set of quinoline derivatives (Compound **C**) to target α-glucosidase were synthesized. In vitro assessments demonstrated IC_50_ values in the range of 6.20 to > 50 µM^24^. A series of quinolone- bis(indolyl)methane hybrids bearing a wide range of functional groups (Compound **D**) were synthesized as α-glucosidase inhibitors. Most of them showed significant α-glucosidase inhibitory activity compared to acarbose (IC_50_ = 154.7 ± 1.9 μM)^25^.

Also, benzimidazole pharmacophore is well known for its α-glucosidase inhibitory activities with strong interactions with the active site^21^. It was identified as anti-α-glucosidase agents via the random screening of the in-house compound library^26^. The follow-up optimization of hit **E** resulted in a series 2-phenyl-1H-benzo[d]imidazole derivatives (compound **F**). The kinetic study of **F** exhibited non-competitive inhibition with no cytotoxicity against LO2 cells^27^. Zawawi and coworkers prepared twenty-six analogs of benzimidazole derivatives (compound **G**) with IC_50_ values ranging from 8.40—12.49 μM which showed potency greater than standard acarbose (IC_50_ = 774.5 ± 1.94)^28^. The high potency of benzimidazole was also confirmed in the previous studies (compound **H**)^28,29^.

Regarding that, the α-glucosidase inhibitory activity is affected by combining the quinoline and benzimidazole moieties in one molecule and inspired by these results aiming to develop more effective α-glucosidase inhibitors, novel series of benzimidazole-thioquinoline hybrids were designed. Also, it was assumed that sulfur atoms might provide special interactions with critical residues of the enzyme binding site. All derivatives were synthesized and evaluated for α-glucosidase inhibition to identify lead molecules. The structure–activity relationships (SARs), molecular dynamic simulations (in silico), as well as kinetic assessments were also performed.

## Results and discussion

### Chemistry

**Figure 2:**
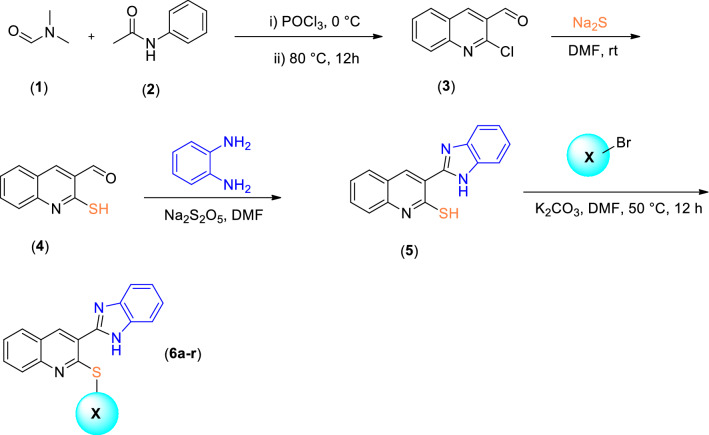
Synthesis of compounds **6a–r.**

The synthetic route to target compounds **6a–r** is represented in Fig. 2. First, commercially available N,N-dimethylformamide (**1**) was reacted with phosphoryl chloride at 0 °C then phenyl-acetamide was added dropwise, and the mixture was stirred at 80 °C for 12 h to afford compound **3**. The crude product was purified by recrystallization in ethanol. Sodium sulfide was added to 2-chloroquinoline-3-carbaldehyde (compound **3**) in DMF and was stirred at room temperature for 2 h leading to the formation of 3-formyl-2-mercaptoquinoline (**4**). Compound **5** was synthesized by the reaction of commercially available o-phenylenediamine with compound **4** in the presence of the catalytic amount of Na_2_S_2_O_5_ under reflux conditions in DMF for 2 h. Finally, different substituted **6a–r** were synthesized by the reaction of different alkyl or aryl halides with compound **5** in DMF at 50 °C for 12 h. The crude products were purified by recrystallization in ethanol. The structures of purified products were confirmed by IR, ^1^H -NMR, ^13^C -NMR, and elemental analysis (Supplementary files 1, 2).

### Evaluation of α-glucosidase inhibitory activity and structure–activity relationships

In vitro α-glucosidase inhibitory activity of synthesized compounds, **6a–r** was performed compared with acarbose as the reference inhibitor. The results of the anti-α-glucosidase assay were presented in Table 1 in terms of IC_50_. In this series, all compounds had promising inhibition against α-glucosidase with IC_50_ values ranging from 28.0 to 663.7 µM compared with a positive control with an IC_50_ value of 750.0 µM.

**Table 1:** α-glucosidase inhibitory activity of **6a–r**


Compounds	X	IC_50_ ± SD (µM)^a^
6a	Benzyl	153.7 ± 0.9
6b	2-Fluorobenzyl	187.9 ± 2.4
6c	3-Fluorobenzyl	76.7 ± 0.7
6d	4-Fluorobenzyl	80.9 ± 1.1
6e	2-Chlorobenzyl	663.7 ± 1.2
6f	3-Chlorobenzyl	48.2 ± 0.4
6g	4-Chlorobenzyl	96.6 ± 0.1
6h	2-Bromobenzyl	133.5 ± 1.3
6i	3-Bromobenzyl	65.5 ± 2.0
6j	4-Bromobenzyl	28.0 ± 0.6
6k	3,4-Dichlorobenzyl	99. 4 ± 0.7
6l	2-Methylbenzyl	195.7 ± 0.6
6m	3-Methylbenzyl	158.4 ± 2.4
6n	4-Methylbenzyl	116.6 ± 0.5
6o	2,3-Dimethylbenzyl	126.9 ± 0.5
6p	4-Nitrobenzyl	89.2 ± 1.3
6q	4-Methoxybenzyl	67.3 ± 0.8
6r	Ethyl	300.7 ± 2.0
Acarbose	–	750.0 ± 5.0

^a^Data represented in terms of mean ± SD.

The unsubstituted benzyl derivative **6a** showed a considerable inhibitory effect against α-glucosidase with IC_50_ values of 153.7 μM. Different moieties were introduced at different positions of the benzyl pendant to investigate the effect of substitution on the phenyl ring. First, the inhibitory effect of halogen groups was evaluated. The introduction of a *meta* fluorine (**6c**) or *para* fluorine (**6d**) group on the benzyl ring improved the activity compared to the unsubstituted one, and there is no significant between the *meta* (**6c**) and *para*-substituted (**6d)** fluorine groups. However, the *ortho* fluorine substitution (**6b**) deteriorated the potency.

Next, Cl was substituted at the various position of the phenyl pendant. 3-chlorobenzyl (**6f.**, IC_50_ = 48.2 μM) exhibited significant inhibitory activity in comparison with **6g** (X = 4-chlorobenzyl, IC_50_ = 96.6 μM), **6k** (X = 3,4-dichlorobenzyl, IC_50_ = 99.4 μM) and **6e** (X = 2-chlorobenzyl, IC_50_ = 663.7 μM).

The 4-bromobenzyl derivative (**6j,** IC_50_ = 28.0 μM) was the most promising α-glucosidase inhibitor of this series, with around a 30-fold improvement in the potency compared with positive control. Similar to the previous sets, *ortho*-bromine substitution (**6h**) was inferior to the potency.

Subsequently, the inhibitory effect of electron-donating groups was assessed. The introduction of a 2-methyl group (**6l**) slightly reduced the activity (IC_50_ = 195.7 μM) compared to an unsubstantiated one (**6a**). However, this compound still demonstrated better activity compared to acarbose as the positive control. Similar to previous derivatives, the replacement of the position from *ortho* (**6l**) to *meta* (**6m**) or *para* (**6n**) empowers the potency to the IC_50_ value of 158.4 and 116.6 µM, respectively. It seems that the optimum position of the electron-withdrawing group was the *para* position. Furthermore, methyl multi-substitutions also disclosed improvement in the activity compared to unsubstituted derivative (**6a**).

With the promising results on the α-glucosidase inhibitory activity of different substitutions, the hydrogen bond interacting motifs were also synthesized. **6p** (X = 4-nitrobenzyl) and **6q** (X = 4-methoxybenzyl) demonstrated good activity with IC_50_ values of 89.2 and 67.3 μM, respectively. Based on enzymatic inhibitory activity, **6r** containing ethyl fragment with IC_50_ = 300.7 μM deteriorated the activity compared to all aromatic substituted groups except **6e**. It seems that aliphatic substitutions are not favorable. Also, it was understood that *ortho* substitution and aliphatic moiety on the benzyl ring reduced the inhibitory potencies, which could be due to the steric hinder at this position.

### Enzyme kinetic studies

**Figure 3:**
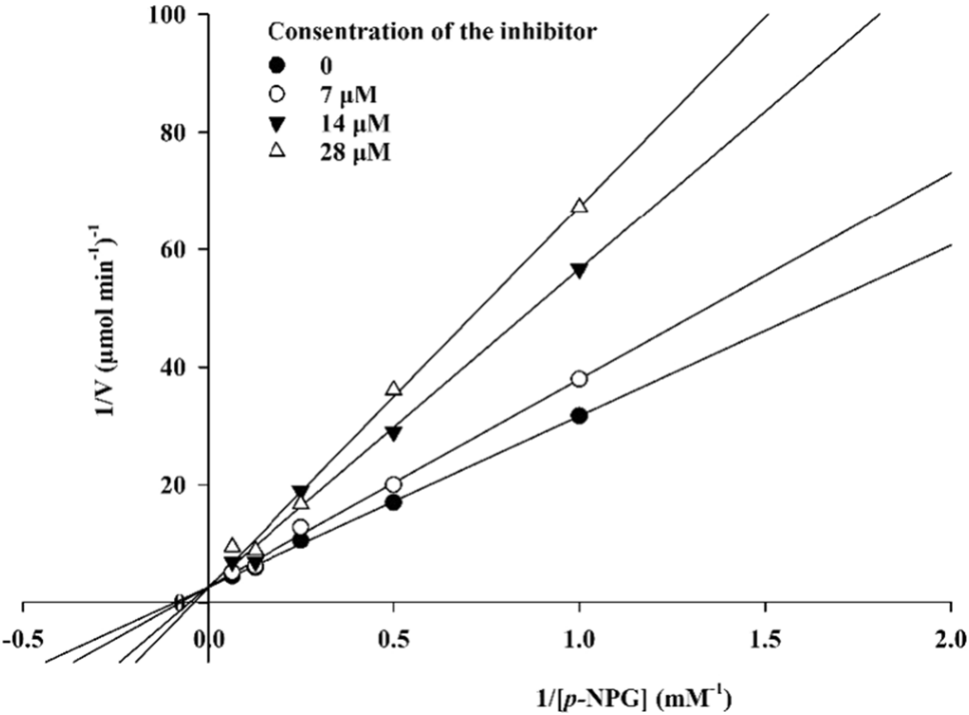
The Lineweaver–Burk plot in the absence and presence of different concentrations of **6j.**

**Figure 4:**
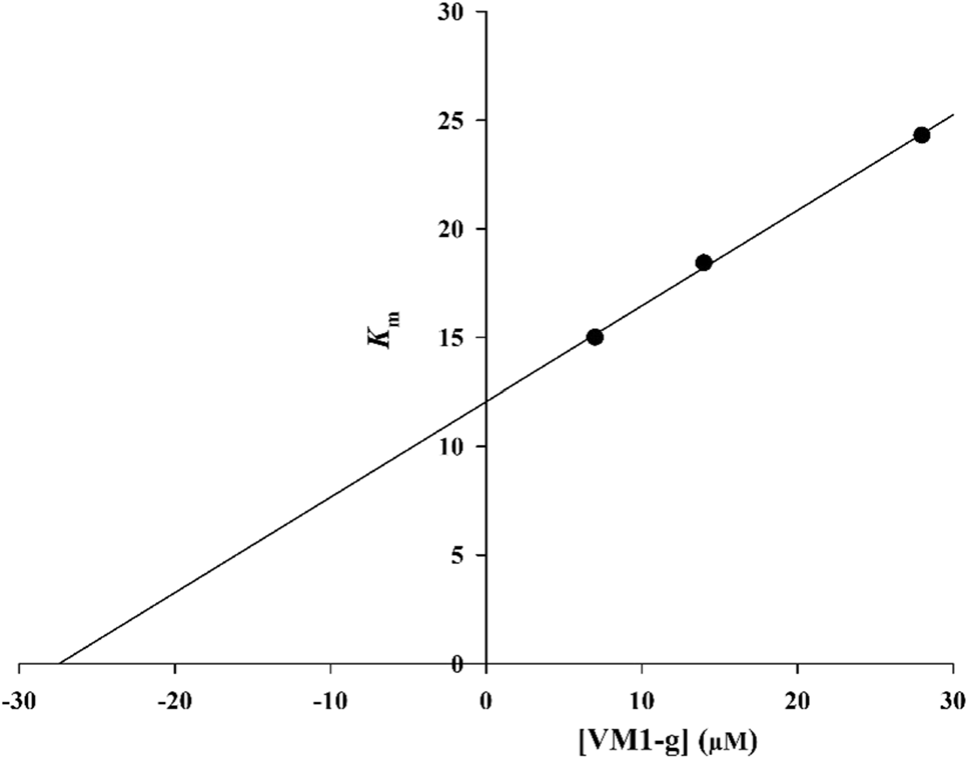
The secondary plot between *K*_m_ and various concentrations of **6j.**

According to Fig. 3, the Lineweaver–Burk plot showed that the *K*_m_ gradually increased and *V*_*max*_ remained unchanged with increasing inhibitor concentration indicating a competitive inhibition. The results show that **6j** bound to the active site on the enzyme and competed with the substrate for binding to the active site. Furthermore, the plot of the *K*_m_ versus different concentrations of inhibitor gave an estimate of the inhibition constant, *K*_i_ of 28.1 µM (Fig. 4).

### Docking Study

Molecular docking was analyzed in order to gain an understanding of the binding mechanism of fluorine substituted derivatives which is less bulky compared with bromine substituted derivatives with bulkier moiety. First, the molecular docking validation was performed to dock acarbose as a native ligand inside the α-glucosidase and the alignment of the best pose of acarbose in the active site of the enzyme and crystallographic ligand recorded an RMSD value less than 2 Å confirming the accuracy of docking. Then, the docking procedures were applied to all synthesized derivatives. It was reported that Glu276, His348, and Asp349 play critical roles in the catalytic mechanism of in α-glucosidase. The detailed interactions of all derivatives are presented in Table 2. As can be seen, **6j** exhibited the best value with a GlideScore of -8.08 and participated in critical interactions with Asp349 and Asp408 categorized as essential residues of the binding site. Also, some studies exhibited the important residues of Asp 214, Glu 276, Arg 312, Asp 408, and Arg 439 within the enzyme's binding site^30–32^. The other derivatives **6h** and **6i** showed values of -6.55 and -6.85 GlideScore. Although **6b** recorded the second top GlideScore value (-7.95), it exhibited low potency in the biological assessments. A closer lock at this interaction reveals that benzimidazole moiety participates in unfavorable interactions with Phe157. Also, **6c** and **6d** bearing 3-flurobenzy and 4-flurobenzyl exhibited unfavorable interactions through benzimidazole moieties.

**Table 2:** The predicted binding energy of all derivatives with the desired enzyme.

Compound	GlideScore	Moiety	Residue	Type of interaction
6b	-7.95	Benzimidazole	Phe157	H-bound
		Benzimidazole	Phe157	One bad interaction
		Benzimidazole	Lys239	Pi-pi stacking
		Benzimidazole	Arg312	H-bound
**6c**	-5.81	Benzimidazole	Tyr71	Pi-pi stacking
		Benzimidazole	Tyr177	Pi-pi stacking
		Benzimidazole	Asp214	Three bad interactions
		Quinoline	Arg312	Pi-cation
		3-Fluorobenzyl	Tyr313	Pi-pi stacking
		Benzimidazole	Asp349	H-bound
		Benzimidazole	Arg439	Pi-cation
**6d**	-6.16	Benzimidazole	Tyr71	Pi-pi stacking
		3-Fluorobenzyl	Phe157	Pi-pi stacking
		Benzimidazole	Phe177	Pi-pi stacking
		Benzimidazole	Arg439	Two bad interactions
		Benzimidazole	Arg439	Pi-cation
		Benzimidazole	Arg439	Pi-cation
**6h**	-6.55	Benzimidazole	Phe157	Pi-pi stacking
		Benzimidazole	Phe157	Pi-pi stacking
		Benzimidazole	Phe157	one bad interaction
		Benzimidazole	His279	Pi-cation
		Quinoline	Phe311	Pi-pi stacking
		Quinoline	Arg312	Pi-cation
**6i**	-6.85	Benzimidazole	Phe157	H-bound
		Benzimidazole	Lys239	Pi-pi stacking
		Benzimidazole	Arg312	H-bound
		4-bromobenzyl	Gln350	Halogen interaction
**6j**	-8.08	Benzimidazole	Tyr71	Pi-pi stacking
		Quinoline	His279	Pi-pi stacking
		Quinoline	Phe300	Pi-pi stacking
		Quinoline	Phe300	Pi-pi stacking
		Benzimidazole	Asp349	H-bound
		4-Bromobenzyl	Asp408	Halogen interaction

### Molecular dynamic simulations

**Figure 5:**
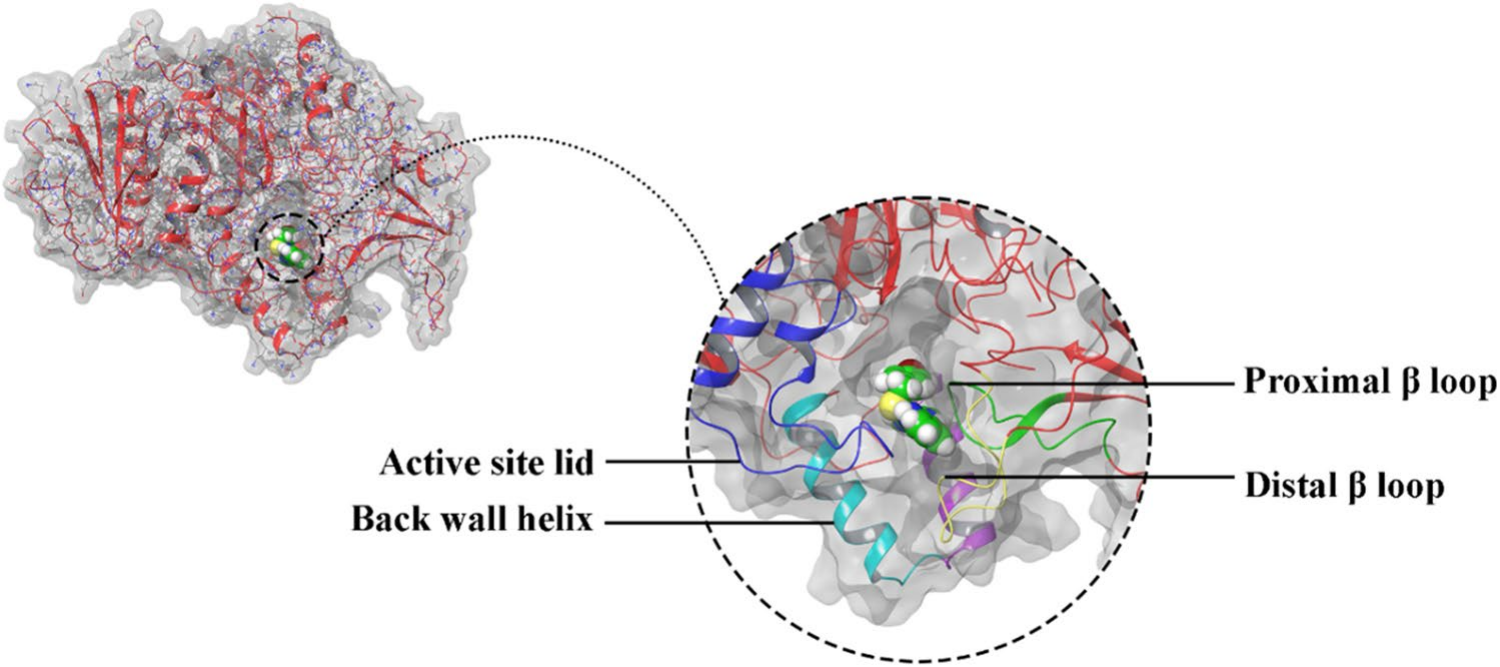
The structure of modeled enzyme active site in complex with compound **6j** consisted of active site lide (blue), back wall helix (cyan), distal β-loop (yellow), and proximal β-loop (red).

Considering that the α-glucosidase x-ray crystallographic structure of *S. cerevisiae* is unavailable, the in silico study was performed using the homology-modeled enzyme previously reported in our articles^33^. The overall architecture of this enzyme is similar to the human intestinal α-glucosidase enzyme. According to our in silico evaluations (Fig. 5), the active site pocket of the enzyme consists of a functional site lid (blue- residues 305–315), the back wall helix (Teal -residues 425–437), and two β-sheet loops demonstrated in the green and yellow cartoon (residues 150 -160 and 250 -260).

**Figure 6:**
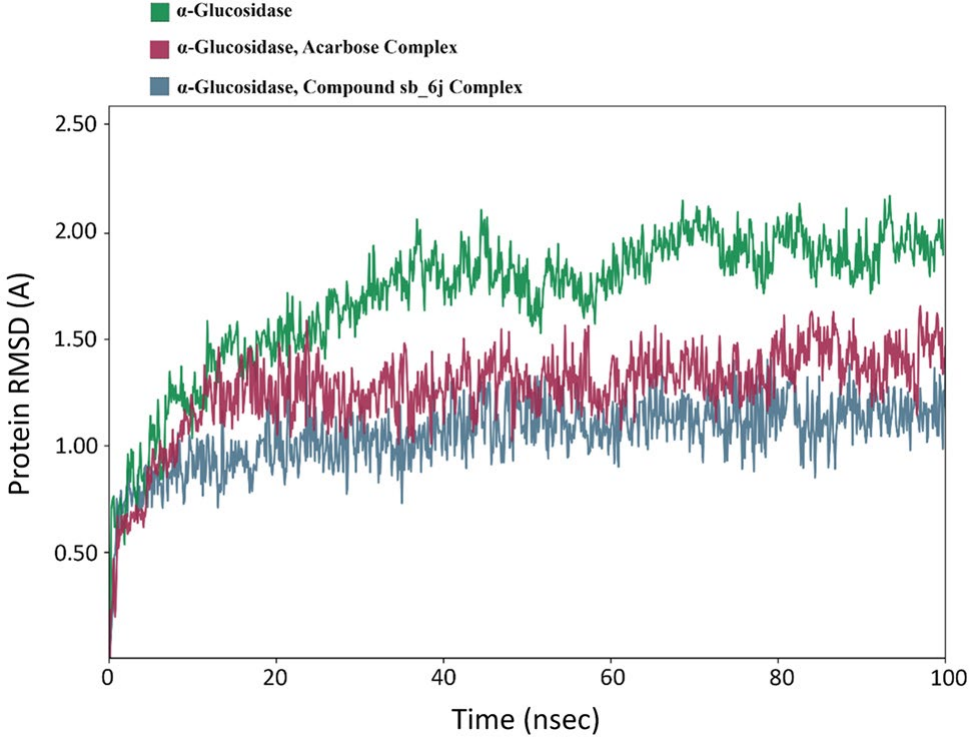
The RMSD values of the α-glucosidase apo-enzyme (green), acarbose in complex with the α-glucosidase enzyme (red), and compound **6j** in complex with the α-glucosidase enzyme (blue).

The stability of the protein–ligand complex trajectories was assessed with the enzymes’ backbone Root Mean Square Deviation (RMSD) during the 100 ns MD simulation. The RMSD comparison of apo-enzyme alongside the enzyme in complex with acarbose as natural ligand and compound **6j** as the most potent inhibitor is demonstrated in Fig. 6. The RMSD value of α-glucosidase-acarbose complex stabilized after 10 ns with the range of (1.25 Å). It remained in the same situation with fewer fluctuations till the end of the simulation. On the other hand, the α-glucosidase enzyme took longer to stabilize (about 20 ns) and had higher values of fluctuation until the end of the simulation. The RMSD plot of α-glucosidase with **6h** had more fluctuations than the latter complex, the complex stabilized after 5 ns around the (1.00 Å) until the end of the simulation with an average RMSD value of 2 Å. The overall RMSD values of both complexes didn’t seem to have a significant difference which can be contributed to the low steric hindrance and high flexibility of compound **6j**. However, the RMSD of apo-enzyme had a significant difference with complexes which can be justified by the absence of any potent ligand.

**Figure 7:**
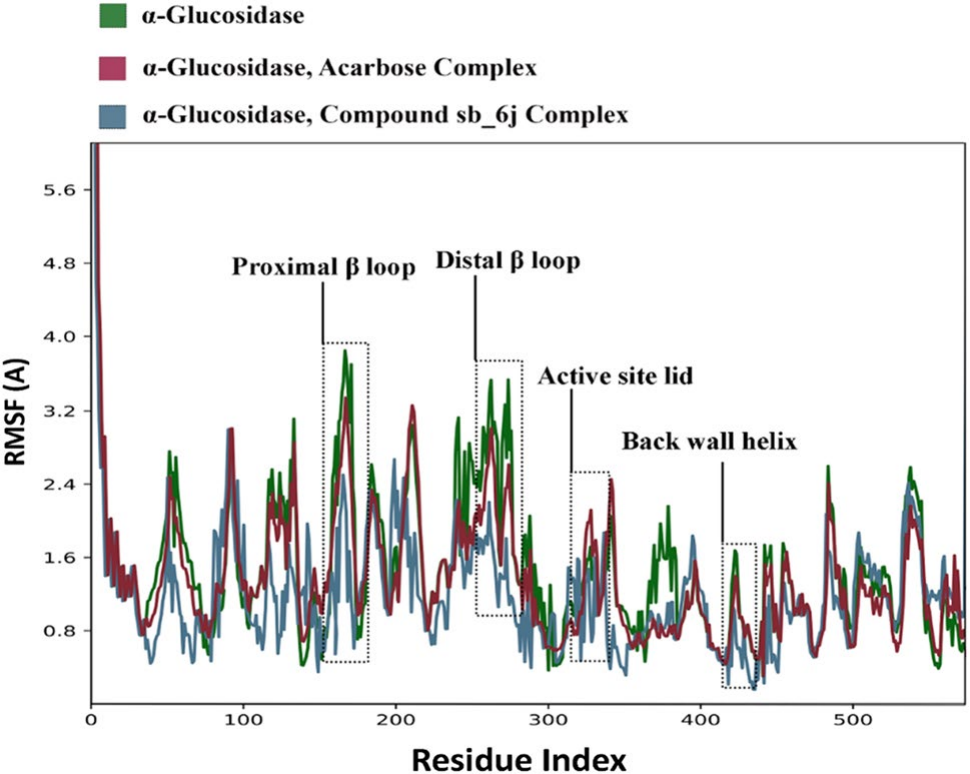
The RMSF values of α-glucosidase, acarbose- α-glucosidase and compound **6j** in complex with α-glucosidase.

The Root Mean Square Fluctuations (RMSF) of Cα atoms from both complexes and the α-glucosidase revealed the detailed mechanism of the ligand interaction with the enzyme. Upon the binding of ligands to the α-glucosidase, residues movement decrease due to non-bonding interactions between the ligand and the enzyme^34^. The most important residues of the active site, including the functional site lid, the back-wall helix, and two β-sheet loops, mostly had a smaller RMSF value than the acarbose (Fig. 7).

**Figure 8:**
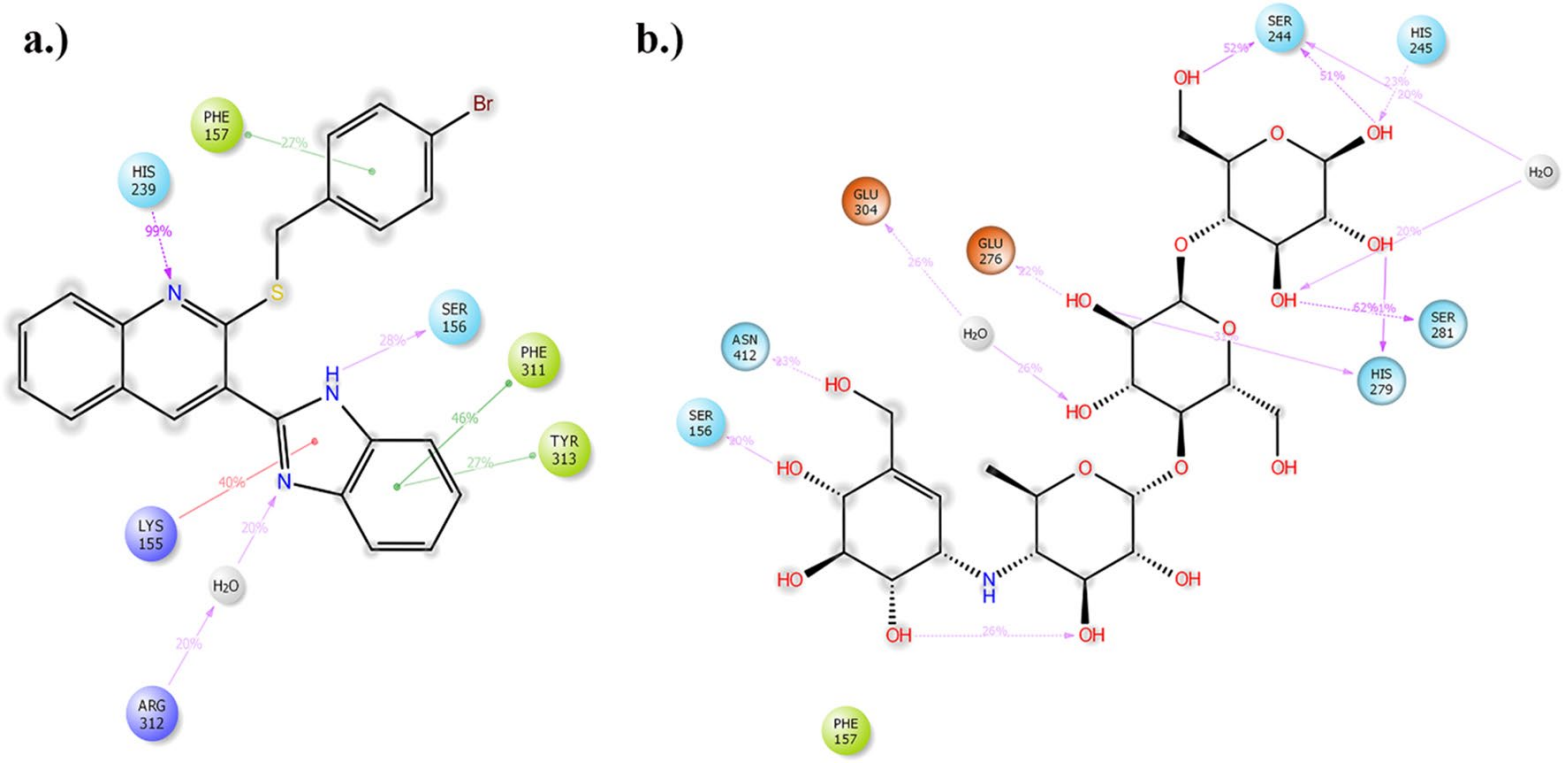
(**a**) 2D presentation of compound **6j** interactions with the active site of the enzyme (**b**) 2D presentation of acarbose interactions with the active site of the enzyme.

The interactions of compound **6j** with the active site pocket of the enzyme, which has been present in more than 20% of the duration of simulations, are demonstrated in Fig. 8. His239 made a major H-bond interaction with the nitrogen atom of the quinoline system, two pi–stacking interactions was observed between the active side lid residues Phe311 and Tyr313. From the proximal β loop of the enzyme residues Phe157, Lys155 and Ser156 were found to have pi-pi staking, pi-stacking, and H-bond interactions, respectively. Conversely, acarbose showed multiple H-bond interactions with Ser244, His245, Ser281, His289, Ser156, and Asn412. Other interactions included charged interaction with Glu276 and double water bridged interactions with Ser244 and Glu304.

### ADME-Toxicity profiles and physicochemical properties

The physicochemical properties and pharmacokinetic profile of the new benzimidazole-thioquinoline hybrid were calculated as part of preclinical drug development studies^35^. The intestine is usually the primary site for orally administered drugs, and a value of more than 30% is considered good absorption. As can be seen in Table 3, the good human intestinal absorption of all compounds caused fast absorption from the intestine to the bloodstream. The steady-state volume of distribution (VDss) is the theoretical volume, and the higher the VD, the more of a drug is distributed in tissue rather than plasma. Log VDss < -0.15 is considered low, while log VDss > 0.45 categorize as high. All derivatives showed moderate VDss value with steady distribution in blood. The cytochrome P450’s are responsible for the metabolism of many drugs and an important detoxification enzyme in the body. The drug-metabolizing enzymes most studied are the cytochrome P450 superfamily with different isozymes, 2D6, 3A4, 1A2, 2C19, 2C9, 2D6, and 3A4 which cover a wide range of chemical structures in drug metabolism and distribution. The differences in these isoforms comebacks to the site of expression, and type of drug to be detoxified, which comes back to the structure of the enzyme and its sequences^36–38^. Results of Table 3 showed that the molecules are likely to be metabolized by 3A4, which affect the pharmacokinetics of these drugs. P450 3A4 (abbreviated CYP3A4), mainly found in the liver and the intestine, metabolizing broad substrate from most therapeutic categories and many endogenous substances. On the other hand, CYP2D6 is primarily expressed in the liver and central nervous system and is involved in antipsychotic metabolism. Cytochrome P450 inhibitors can increase the bioavailability of drugs with a high first-pass metabolism, and the inhibition of CYP450 isoform can result in the accumulation of parent drug concentrations.

**Table 3:** ADMET prediction of the synthesized derivatives as α-glucosidase inhibitors.

	Absorption	Distribution	Metabolism							Excretion	Toxicity
	Human Intestinal Absorption (% absorbed)	VDss (logL/Kg)	2D6	3A4	1A2	2C19	2C9	2D6	3A4	Total Clearance (log mL/min/kg)	Oral rat acute toxicity (mol/kg)
			Substrate		Inhibitor						
**6a**	84.416	0.051	No	Yes	Yes	Yes	Yes	Yes	Yes	1.051	2.44
**6b**	83.65	0.047	No	Yes	Yes	Yes	Yes	Yes	Yes	0.956	2.44
**6c**	83.65	0.047	No	Yes	Yes	Yes	Yes	Yes	Yes	0.99	2.44
**6d**	83.65	0.047	No	Yes	Yes	Yes	Yes	Yes	Yes	1.006	2.44
**6e**	82.755	0.051	No	Yes	Yes	Yes	Yes	Yes	Yes	0.961	2.44
**6f.**	82.755	0.051	No	Yes	Yes	Yes	Yes	Yes	Yes	0.961	2.44
**6g**	82.755	0.051	No	Yes	Yes	Yes	Yes	Yes	Yes	0.961	2.44
**6h**	82.688	0.051	No	Yes	Yes	Yes	Yes	Yes	Yes	1.047	2.44
**6i**	82.688	0.051	No	Yes	Yes	Yes	Yes	Yes	Yes	0.933	2.44
**6j**	82.688	0.051	No	Yes	Yes	Yes	Yes	Yes	Yes	0.896	2.44
**6k**	81.094	0.051	No	Yes	Yes	Yes	Yes	Yes	Yes	1.084	2.44
**6l**	84.214	0.051	No	Yes	Yes	Yes	Yes	Yes	Yes	1.035	2.44
**6m**	84.214	0.051	No	Yes	Yes	Yes	Yes	Yes	Yes	1.035	2.44
**6n**	84.214	0.051	No	Yes	Yes	Yes	Yes	Yes	Yes	1.035	2.44
**6o**	84.011	0.054	No	Yes	Yes	Yes	Yes	Yes	Yes	1.132	2.44
**6p**	82.091	0.085	No	Yes	Yes	Yes	Yes	No	Yes	0.778	2.48
**6q**	84.413	0.049	No	Yes	Yes	Yes	Yes	Yes	Yes	1.08	2.44
**6r**	84.011	0.043	Yes	No	Yes	Yes	Yes	Yes	No	1.037	2.42
**Acarbose**	4.172	-0.836	No	No	No	No	No	No	No	0.428	2.45

Total clearance is a combination of hepatic and renal clearance expressed in the log (ml/min/kg). Low renal clearance is defined as ≤ 0.1 mL/min/kg, moderate as > 0.1 to < 1 mL/min/kg, and high as 1 > mL/min/kg^39^. In most cases, synthesized compounds showed moderate total clearance. Oral rat acute toxicity (LD_50_) is the amount of material given all at once that causes the death of 50% (one-half) of a group of test animals. The LD50 is one way to measure a material's short-term poisoning potential (acute toxicity) compounds, and a value less than 0.5 is categorized as high toxic demonestrated LD50 value in the range of 2.42 to 2.48 mol/kg.

Also the physicochemical and molecular properties from the SwissADME website were presented in Table 4^40^. Lipinski’s rule of five is a valid method to evaluate the drug-likeness criteria of compounds (lipophilicity ≤ 5, molecular weight ≤ 500, hydrogen bond donor (HBD) ≤ 5 (OH and NH groups), and hydrogen bond acceptor (HBA) ≤ 10 (N and O atoms). As seen in Table 4, all derivatives have acceptable molecular weight, number of rotatable bonds, number of H-bond acceptors, and number of H-bond donors. However, there is the validation of lipophilicity optimum range of all compounds except **6r.**

**Table 4:** Drug-likeness properties of derivatives.

Compound	MW	Num. rotatable bonds	Num. H-bond acceptors	Num. H-bond donors	Log *P*
**6a**	367.477	4	3	2	6.071
**6b**	385.467	4	3	1	6.21
**6c**	385.467	4	3	1	6.21
**6d**	385.467	4	3	1	6.21
**6e**	401.922	4	3	1	6.72
**6f**	401.922	4	3	1	6.72
**6g**	401.922	4	3	1	6.72
**6h**	446.373	4	3	1	6.83
**6i**	446.373	4	3	1	6.83
**6j**	446.373	4	3	1	6.83
**6k**	436.367	4	3	1	7.38
**6l**	381.504	4	3	1	6.38
**6m**	381.504	4	3	1	6.38
**6n**	381.504	4	3	1	6.38
**6o**	395.53	4	3	1	6.69
**6p**	412.474	5	5	1	5.97
**6q**	397.503	4	3	1	6.69
**6r**	305.406	3	3	1	4.89

## Conclusion

Following our interest in the rational design of α-glucosidase inhibitors; herein, a series of benzimidazole-thioquinolines were designed and synthesized. All derivatives demonstrated promising glucosidase activity inhibitory activities with IC_50_ values of 28.0–663.7 μM compared with the reference compound, acarbose (IC_50_ = 750.0 μM). The SAR data revealed mostly any substitution at the *para* position is favorable regardless of the type of inhibition. Compound **6j** (IC_50_ = 28.0 ± 0.6 μM) as the most potent inhibitor revealed competitive inhibition patterns in the kinetic experiments. Molecular docking studies justify the designing strategy as benzimidazole recorded H-bound interaction with Asp616 and the linker participate in pi-sulfur interaction with the binding site. Noteworthy the least active compound exhibited unfavorable interaction which justifies its low potency. MD studies showed that **6j**-enzyme were stable during the simulation time and participated in pronounced interaction with the α-glucosidase active site through several H-bound interactions. Computed physicochemical and ADMET properties exhibited the druggability of the developed derivatives. These findings will be prominent for the structural design of a-glucosidase inhibitors in the development of novel anti-diabetic agents.

## Methods and materials

### Chemistry

#### 3-(1H-benzo[d]imidazol-2-yl)-2-(benzylthio) quinoline (**6a**)

IR (ν,cm^-1^): 3376, 1642, 1627, 1450, 1580. ^1^HNMR (300 MHz, DMSO-*d*_*6*_) δ: 13.00 (brs, 1H, NH), 8.69 (s, 1H, H_4 quinoline_), 8.11–7.78 (m, 8H, Ar), 7.64–7.57 (m, 3H, Ar), 7.26–7.24 (m, 2H, Ar), 4.64 (s, 2H, CH_2_). ^13^CNMR (75 MHz, DMSO-*d*_*6*_) δ: 158.2, 149.8, 149.0, 148.1, 147.7, 137.9, 132.7, 132.0, 129.7,128.8, 127.8, 126.1, 124.7, 124.3, 124.0, 117.0, 35.0. Anal. Calcd for C_23_H_17_N_3_S (367.47): C, 75.18; H, 4.66; N, 11.44. Found: C, 75.25; H, 4.56; N, 11.40%.

#### 3-(1H-benzo[d]imidazol-2-yl)-2-((2-fluorobenzyl)thio)quinoline (**6b**)

IR (ν,cm^-1^): 3385, 1645, 1630, 1460, 1570. ^1^HNMR (300 MHz, DMSO-d_6_) δ: 13.14 (s, 1H, NH), 8.43 (s, 1H, H_4 quinoline_), 8.04 (d, 1H, *J* = 9 Hz, H_6 quinoline_), 7.94 (d, 1H, *J* = 8.8 Hz, H_8 quinoline_),7.90 (t, 1H, *J* = 8.8 Hz, H_7 quinoline_), 7.86–7.81 (m, 3H, Ar), 7.80–7.75 (m, 1H, Ar), 7.68–7.60 (m, 3H, Ar), 7.42 (s, 1H, Ar,7.23–7.11 (m, 2H, Ar), 4.55 (s, 2H, CH_2_). ^13^CNMR (75 MHz, DMSO-*d*_*6*_) δ: 157.54163.4 (d, CF, ^1^*J*_CF_ = 284.5 Hz), 149.29, 147.09, 136.76, 133.06, 131.54, 128.53, 127.87, 126, 125.94, 125.25, 123.60, 122.91, 116.69, 115.96. Anal. Calcd for C_23_H_16_FN_3_S (385.46): C, 71.67; H, 4.18, N, 10.90. Found: C, 71.78; H, 4.24, N, 19.82%.

#### 3-(1H-benzo[d]imidazol-2-yl)-2-((3-fluorobenzyl)thio)quinoline (**6c**)

IR (ν,cm^-1^): 3380, 1647, 1625, 1462, 1580. ^1^HNMR (300 MHz, DMSO-d_6_) δ: 12.99 (s, 1H, NH), 8.67 (s, 1H, H_4 quinoline_), 8.03–7.99 (m, 2H, H_6,8 quinoline_), 7.81 (t, 1H, *J* = 9 Hz, H_7 quinoline_), 7.70 (brs, 1H, Ar), 7.57 (t, 1H, *J* = 9 Hz, Ar), 7.32–7.25 (m, 5H, Ar, H_9 quinoline_), 6.99 (t, 1H, *J* = 9 Hz, Ar), 4.54 (s, 2H, CH_2_). ^13^CNMR (75 MHz, DMSO-*d*_*6*_)δ 157.92(d, CF, ^1^*J*_CF_ = 289.5 Hz), 148.95, 147.08, 143.99, 139.19, 136.66, 135.05, 132.07, 131.64, 128.79, 126.77, 125.09, 123.76, 123.16, 122.42, 119.79, 112.03, 36.63.. Anal. Calcd for C_23_H_16_FN_3_S (385.46): C, 71.67; H, 4.18; N, 10.90. Found: C, 71.58; H, 4.09; N, 10.98%.

#### 3-(1H-benzo[d]imidazol-2-yl)-2-((4-fluorobenzyl)thio)quinoline (**6d**)

IR (ν,cm^-1^): 3380, 1649, 1630, 1455, 1579. ^1^HNMR (300 MHz, DMSO-d_6_) δ: 12.91 (s, 1H, NH), 8.67 (s, 1H, H_4 quinoline_), 8.04–7.97 (m, 2H, H_6,8 quinoline_), 7.81 (t, 1H, *J* = 9 Hz, H_7 quinoline_), 7.64–7.50 (m, 5H, H_9 quinoline_,Ar), 7.24 (brs, 2H, Ar), 7.07 (t, 2H, *J* = 9 Hz, Ar), 4.96 (s, 2H, CH_2_). ^13^CNMR (75 MHz, DMSO-*d*_*6*_) δ: 162.5 (d, CF, ^1^*J*_CF_ = 289.5 Hz), 158.9, 149.9, 148.2, 138.0, 136.2, 132.8, 136.2, 132.8, 132.6, 129.8, 128.8, 126.1, 124.5, 124.0, 116.6, 116.2, 34.8. Anal. Calcd for C_23_H_16_FN_3_S (385.46): C, 71.67; H, 4.18; N, 10.90. Found: C, 71.58; H, 4.10; N, 10.99%.

#### 3-(1H-benzo[d]imidazol-2-yl)-2-((2-chlorobenzyl)thio)quinoline (**6e**)

IR (ν,cm^-1^): 3369, 1645, 1630, 1450, 1580. ^1^HNMR (300 MHz, DMSO-d_6_) δ: 13.01 (s, 1H, NH), 8.69 (s, 1H, H_4 quinoline_), 8.05 (d, 1H, *J* = 9 Hz, H_6 quinoline_), 7.81 (t, 3H, *J* = 9 Hz, Ar), 7.74–7.39 (m, 5H, Ar), 7.26–7.22 (m, 4H, Ar), 4.64 (brs, 1H, CH_2_), 4.66 (s, 2H, CH_2_). ^13^CNMR (75 MHz, DMSO-*d*_*6*_) δ: 158.7, 150.0, 148.2, 145.0, 138.0, 137.1, 136.0, 134.9, 133.1, 132.7, 130.8, 130.4, 129.7, 128.8, 128.6, 127.7, 126.1, 124.6, 124.5, 123.3, 120.8, 112.7, 33.7. Anal. Calcd for C_23_H_16_ClN_3_S (401.91): C, 68.73; H, 4.01; N, 10.46;Found: C, 68.67; H, 3.91; N, 10.35%.

#### 3-(1H-benzo[d]imidazol-2-yl)-2-((3-chlorobenzyl)thio)quinoline (**6f**)

IR (ν,cm^-1^): 3372, 1640, 1625, 1454, 1585. ^1^HNMR (300 MHz, DMSO-d_6_) δ: 13.06 (s, 1H, NH), 8.73 (s, 1H, H_4 quinoline_), 8.06–8.01 (m, 2H, H_6,8 quinoline_), 7.85 (t, 1H, *J* = 9 Hz, H_7 quinoline_), 7.78–7.74 (m, 1H, Ar), 7.63–7.59 (m, 2H, Ar), 7.54–7.50 (m, 1H, Ar), 7.42 (s, 1H, Ar), 7.33–7.24 (m, 15H, Ar), 4.56 (s, 2H, CH_2_). ^13^CNMR (75 MHz, DMSO-*d*_*6*_) δ: 157.6, 148.9, 147.1, 141.9, 137.0, 133.1, 131.7, 131.0, 130.5, 129.7, 129.1, 128.8, 128.6, 127.7, 127.2, 126.8, 125.1, 123.4, 122.3, 119.7, 112.0, 34.0. Anal. Calcd for C_23_H_16_ClN_3_S (401.91): C, 68.73; H, 4.01, N, 10.46. Found: C, 68.65; H, 4.08, N, 10.40%.

#### 3-(1H-benzo[d]imidazol-2-yl)-2-((4-chlorobenzyl)thio)quinoline (**6g**)

IR (ν,cm^-1^): 3372, 1640, 1625, 1454, 1585. ^1^HNMR (300 MHz, DMSO-d_6_) δ: 12.97 (s, 1H, NH), 8.66 (s, 1H, H_4 quinoline_), 8.03–7.50 (m, 9H, Ar), 7.31–7.23 (m, 3H, Ar), 4.52 (s, 2H, CH_2_). ^13^CNMR (75 MHz, DMSO-*d*_*6*_) δ: 155.3, 148.2, 146.7, 145.9, 141.9, 139.3, 138.0, 132.7, 129.8, 129.6, 128.8, 127.7, 126.1, 124.6, 123.3, 120.7, 112.9, 34.9. Anal. Calcd for C_23_H_16_ClN_3_S (401.91): C, 68.73; H, 4.01; N, 10.46. Found: C, 68.65; H, 4.08, N, 10.39%.

#### 3-(1H-benzo[d]imidazol-2-yl)-2-((2-bromobenzyl)thio)quinoline (**6h**)

IR (ν,cm^-1^): 3380, 1645, 1635, 1452, 1577. ^1^HNMR (300 MHz, DMSO-d_6_) δ: 13.00 (s, 1H, NH), 8.69 (s, 1H, H_4 quinoline_), 8.07–7.97 (m, 2H, *J* = 9 Hz, H_6,8 quinoline_), 7.81 (t, 1H, *J* = 9 Hz, H_7 quinoline_), 7.73–7.58 (m, 5H, Ar, H_9 quinoline_), 7.32–7.13 (m, 4H, Ar), 4.66 (s, 2H, CH_2_). ^13^CNMR (75 MHz, DMSO-*d*_*6*_) δ: 158.7, 149.9, 148.2, 138.7, 138.0, 134.1, 133.2, 132.7, 130.6, 129.8, 129.2, 128.8, 127.8, 126.1, 125.8, 124.4, 124.0, 36.4. Anal. Calcd for C_23_H_16_BrN_3_S (446.36): C, 61.89; H, 3.61; N, 9.41. Found: C, 61.80; H, 3.51; N, 9.52%.

#### 3-(1H-benzo[d]imidazol-2-yl)-2-((3-bromobenzyl)thio)quinoline (**6i**)

IR (ν,cm^-1^): 3384, 1655, 1620, 1459, 1588. ^1^HNMR (300 MHz, DMSO-d_6_) δ: 13.06 (s, 1H, NH), 8.73 (s, 1H, H_4 quinoline_), 8.04 (d, 1H, *J* = 8.9 Hz, H_6 quinoline_), 7.98 (d, 1H, *J* = 8.7 Hz, H_8 quinoline_), 7.86 (t, 1H, *J* = 9 Hz, H_7 quinoline_), 7.70–7.62 (m, 3H, Ar), 0.757 (t, 1H, *J* = 9 Hz, Ar), 7.30–7.25 (m, 3H, Ar, 7.13–7.11 (m, 2H, Ar), 4.55 (s, 2H, CH_2_). ^13^CNMR (75 MHz, DMSO-*d*_*6*_) δ: 157.90, 149.10, 147.78, 147.07, 143.04, 141.29, 141.06, 140.15, 136.64, 136.22, 133.61, 131.90, 131.57, 130.94, 128.77, 127.63, 126.74, 125.4, 123.33, 123.25, 123.02, 36.67. Anal. Calcd for C_23_H_16_BrN_3_S (446.37): C, 61.89; H, 3.61, N, 9.41. Found: C, 61.78; H, 3.58, N, 9.55%.

#### 3-(1H-benzo[d]imidazol-2-yl)-2-((4-bromobenzyl)thio)quinoline (**6j**)

IR (ν,cm^-1^): 3374, 1645, 1629, 1454, 1580. ^1^HNMR (300 MHz, DMSO-d_6_) δ: 12.96 (s, 1H, NH), 8.67 (s, 1H, H_4 quinoline_), 8.00–7.23 (m, 12H, Ar), 4.50 (s, 2H, CH_2_). ^13^CNMR (75 MHz, DMSO-*d*_*6*_) δ: 158.0, 149.0, 148.0, 139.7, 137.9, 133.0, 132.5, 129.7, 128.8, 127.7, 126.1, 124.6, 123.3, 120.7, 113.0, 34.9. Anal. Calcd for C_23_H_16_BrN_3_S (446.36): C, 61.89; H, 3.61; N, 9.41. Found: C, 61.77; H, 3.54; N, 9.49%.

#### 3-(1H-benzo[d]imidazol-2-yl)-2-((3,4-dichlorobenzyl)thio)quinoline (**6k**)

IR (ν,cm^-1^): 3376, 1645, 1630, 1452, 1580. ^1^HNMR (300 MHz, DMSO-d_6_) δ: 13.00 (s, 1H, NH), 8.89 (s, 1H, H_4 quinoline_), 8.05–7.97 (m, 2H, H_6,8 quinoline_), 7.85–7.81 (m, 2H, Ar), 7.72 (d, 1H, *J* = 9 Hz, H_7 quinoline_), 7.49–7.26 (m, 4H, Ar), 7.30–7.21 (m, 2H, Ar), 4.52 (s, 2H, CH_2_). ^13^CNMR (75 MHz, DMSO-*d*_*6*_) δ: 158.4, 149.1, 146.7, 145.9, 144.9, 141.9, 138.0, 133.9, 132.8, 132.7, 131.9, 131.7, 131.2, 129.8, 128.7, 127.8, 126.1, 124.7, 123.3, 120.7, 112.9, 34.4. Anal. Calcd for C_23_H_15_C_l2_N_3_S (436.36): C, 63.31; H, 3.46; N, 9.63. Found: C, 63.26; H, 3.38; N, 9.69%.

#### 3-(1H-benzo[d]imidazol-2-yl)-2-((2-methylbenzyl)thio)quinoline (**6l**)

IR (ν,cm^-1^): 3372, 1650, 1633, 1455, 1585. ^1^HNMR (300 MHz, DMSO-d_6_) δ: 8.04 (s, 1H, H_4 quinoline_), 8.04–7.98 (m, 2H, H_6,8 quinoline_), 7.81 (t, 1H, *J* = 9 Hz, H_7 quinoline_), 7.69–7.44 (m, 4H, Ar, H_9 quinoline_), 7.22–7.09 (m, 5H, Ar), 4.53 (s, 2H, CH_2_), 2.37 (s, 3H, CH_3_). ^13^CNMR (75 MHz, DMSO-*d*_*6*_) δ: 159.3, 149.9, 148.3, 138.0, 136.9, 132.6, 131.7, 129.8, 128.7, 127.7, 127.4, 126.1, 124.6, 123.3, 120.7, 112.9, 34.1, 20.4. Anal. Calcd for C_24_H_19_N_3_S (381.49): C, 75.56; H, 5.02; N, 11.01. Found: C, 75.47; H, 5.11; N, 10.92%.

#### 3-(1H-benzo[d]imidazol-2-yl)-2-((3-methylbenzyl)thio)quinoline (**6m**)

IR (ν,cm^-1^): 3382, 1674, 1635, 1465, 1575. ^1^HNMR (300 MHz, DMSO-d_6_) δ: 12.78 (s, 1H, NH), 8.45 (s, 1H, H_4 quinoline_), 8.04–801 (m, 2H, H_6,8 quinoline_), 7.99 (t, 1H, *J* = 8.8 Hz, H_7 quinoline_), 7.84–7.80 (m, 2H, Ar), 7.68–7.62 (m, 2H, Ar), 7.60 (1H, *J* = 8.7 Hz,Ar), 7.01–6.94 (m, 3H, Ar), 4.51 (s, 2H, CH_2_) ), 2.20 (s, 2H, CH_2_). ^13^CNMR (75 MHz, DMSO-*d*_*6*_) δ: 157.99, 137.39, 136.66, 131.59 ,128.81, 127.77, 127.34, 126.78, 125.16, 123.78, 123.39, 35.75, 20.59. Anal. Calcd for C_24_H_19_N_3_S (381.13): C, 75.56; H, 5.02, N, 11.01. Found: C, 75.80; H, 5.18, N, 11.21%.

#### 3-(1H-benzo[d]imidazol-2-yl)-2-((4-methylbenzyl)thio)quinoline (**6n**)

IR (ν,cm^-1^): 3378, 1670, 1648, 1465, 1585. ^1^HNMR (300 MHz, DMSO-d_6_) δ: 13.14 (s, 1H, NH), 8.77 (s, 1H, H_4 quinoline_), 8.02 (d, 1H,* J* = 8.7 Hz, H_5 quinoline_), 8.00 (d, 1H,* J* = 8.5 Hz, H_8 quinoline_) 7.90–7.88 (m, 2H, Ar), 7. 7.78 (d, 1H,* J* = 8.2 Hz, 2H, Ar), 7.68–7.66 (m, 1H, Ar), 7.60 (1H, *J* = 8.1 Hz,Ar), 7.37 (d, 1H,* J* = 8.1 Hz, 2H, Ar), 7.35–7.28 (m, 2H, Ar), 4.76 (s, 2H, CH_2_) ), 2.07 (s, 2H, CH_2_). ^13^CNMR (75 MHz, DMSO-*d*_*6*_) δ: 157.98, 148.99, 147.18, 143.01, 137.38, 136.86, 136.65, 131.58, 128.81, 127.76, 127.33, 126.78, 125.63, 125.14, 123.77, 123.38,35.72, 20.60. Anal. Calcd for C_24_H_19_N_3_S (381.13): C, 75.56; H, 5.02, N, 11.01. Found: C, 75.74; H, 5.09, N, 11.17%.

#### 3-(1H-benzo[d]imidazol-2-yl)-2-((2,3-dimethylbenzyl)thio)quinoline (**6o**)

IR (ν,cm^-1^): 3384, 1658, 1635, 1458, 1575. ^1^HNMR (300 MHz, DMSO-d_6_) δ: 13.15 (s, 1H, NH), 8.79 (s, 1H, H_4 quinoline_), 8.04(d, 1H, *J* = 8.9 Hz, H_6 quinoline_), -7.96 (d, 1H, *J* = 8.8 Hz, H_8 quinoline_), 7.94 (t,1H, *J* = 8.8 Hz Ar), 7.82 (t, 1H, *J* = 9 Hz, Ar), 7.58–7.30 (m, 4H, Ar), 7.14–7.11 (m, 2H, Ar), , 2.29 (s, 2H, CH_3_), 2.20 (s, 2H, CH_3_). ^13^CNMR (75 MHz, DMSO-*d*_*6*_) δ: 157.98, 149.00, 147.19, 137.38, 136.87, 136.66, 131.60, 131.58, 128.81, 127.77, 127.38, 126.77, 125.62, 125.15, 123.78, 123.39, 35.74, 20.59, 14.49. Anal. Calcd for C_25_H_21_N_3_S (395.15): C, 75.92; H, 5.35; N, 10.62. Found: C, 75.87.26; H, 5.38; N, 10.69%.

#### 3-(1H-benzo[d]imidazol-2-yl)-2-((4-nitrobenzyl)thio)quinoline (**6p**)

IR (ν,cm^-1^): 3374, 1639, 1630, 1448, 1582. ^1^HNMR (300 MHz, DMSO-d_6_) δ: 13.00 (s, 1H, NH), 8.66 (s, 1H, H_4 quinoline_), 8.11–7.90 (m, 3H, Ar), 7.83–7.65 (m, 3H, Ar), 7.60–7.45 (m, 3H, Ar), 7.28–7.20 (m, 4H, Ar), 4.64 (brs, 1H, CH_2_), 4.54 (brs, 1H, CH_2_). ^13^CNMR (75 MHz, DMSO-*d*_*6*_) δ: 159.0, 158.2, 149.9, 149.1, 148.2, 144.9, 139.8, 137.9, 136.0, 132.6, 132.6, 132.0, 130.8, 129.7, 128.8, 128.3, 127.8, 127.7, 126.1, 124.7, 123.3, 120.7, 112.9, 35.8. Anal. Calcd for C_23_H_16_N_4_O_2_S (412.46): C, 66.97; H, 3.91; N, 13.58. Found: C, 66.88; H, 3.80; N, 13.65%.

#### 3-(1H-benzo[d]imidazol-2-yl)-2-((4-methoxybenzyl)thio)quinoline (**6q**)

IR (ν,cm^-1^): 3372, 1645, 1633, 1454, 1585. ^1^HNMR (300 MHz, DMSO-d_6_) δ: 12.97 (s, 1H, NH), 8.67 (s, 1H, H_4 quinoline_), 8.04–7.98 (m, 2H, H_6,8 quinoline_), 7.84–7.69 (m, 2H, Ar), 7.61–7.55 (m, 2H, Ar), 7.26–7.14 (m, 3H, Ar), 7.08–7.03 (m, 2H, Ar), 6.74 (d, 1H, J = 9 Hz, Ar), 4.51 (s, 2H, CH_2_), 3.67 (s, 3H, CH_3_). ^13^CNMR (75 MHz, DMSO-*d*_*6*_) δ: 160.6, 149.9, 148.2, 144.9, 141.3, 138.0, 136.0, 132.6, 130.8, 129.8, 127.7, 126.1, 124.6, 123.3, 123.1, 120.7, 116.4, 113.9, 112.9, 56.4, 35.7. Anal. Calcd for C_24_H_19_N_3_OS (397.49): C, 72.52; H, 4.82; N, 10.57. Found: C, 72.47; H, 4.74; N, 10.65%.

#### 3-(1H-benzo[d]imidazol-2-yl)-2-(ethylthio)quinoline (**6r**)

IR (ν,cm^-1^): 3377, 1645, 1630, 1452, 1588. ^1^HNMR (300 MHz, DMSO-d_6_) δ: 13.00 (s, 1H, NH), 8.66 (s, 1H, H_4 quinoline_), 8.01 (d, 1H, *J* = 9 Hz, H_6 quinoline_), 7.96 (d, 1H, *J* = 9 Hz, H_9 quinoline_), 7.81 (t, 1H, *J* = 9 Hz, H_7 quinoline_), 7.76 (d, 1H, Ar), 7.61–7.57 (m, 2H, Ar), 7.30–7.25 (m, 2H, Ar), 3.29 (q, 2H, *J* = 6 Hz, CH_2_), 1.36 (t, 3H, *J* = 6 Hz, CH_3_). ^13^CNMR (75 MHz, DMSO-*d*_*6*_) δ: 158.6, 149.1, 147.5, 144.0, 137.1, 135.0, 131.5, 128.8, 127.8, 126.5, 124.1, 123.5, 122.3, 119.7, 112.0, 24.7, 14.5. Anal. Calcd for C_18_H_15_N_3_S (305.40): C, 70.79; H, 4.95; N, 13.76. Found: C, 70.69; H, 4.86; N, 13.85%.

### In vitro α-glucosidase inhibition assay

α-Glucosidase enzyme (EC3.2.1.20, *Saccharomyces cerevisiae*, 20 U/mg) and substrate (p-nitrophenyl glucopyranoside) were purchased from Sigma-Aldrich. 1 mg of α-glucosidase was dissolved in potassium phosphate buffer (50 mM, pH = 6.8) to obtain the initial activity of 1 U ml^–1^. Then, 20 µl of this enzyme solution was incubated with 135 µl of potassium phosphate buffer and 20 µl of test compound at various concentrations in DMSO. Therefore, the final concentration of the enzyme was about 0.1 U ml^–1^. After 10 min incubation at 37 °C, 25 µl of the substrate at a final concentration of 4 mM was added to the mixture and allowed to incubate at 37 °C for 20 min. Then, the change in absorbance was measured at 405 nm spectroscopically. DMSO (10% final concentration) as control and acarbose as the standard inhibitor were used^41,42^.

DMSO as control (10% final concentration) and acarbose as the standard drug were used. The percentage of inhibition for each entry was calculated by using the following formula:

$$\% \,{\text{Inhibition}} = [({\text{Abs}}\,{\text{control}} - {\text{Abs}}\,{\text{sample}})/{\text{Abs}}\,{\text{control}}] \times 100$$

IC_50_ values were obtained from the nonlinear regression curve using the Logit method.

### Enzyme kinetic studies

The mode of inhibition of the most active compound (**6h**), identified with the lowest IC_50_, was investigated against an α-glucosidase activity with different concentrations of *p*-nitrophenyl *α*-D-glucopyranoside (1–16 mM) as substrate in the absence and presence of **6h** at different concentrations (0, 7, 14, and 28 µM). A Lineweaver–Burk plot was generated to identify the type of inhibition and the Michaelis–Menten constant (*K*_m_) value was determined from the plot between the reciprocal of the substrate concentration (1/[S]) and reciprocal of enzyme rate (1/V) over various inhibitor concentrations. The experimental inhibitor constant (*K*_i_) value was constructed by secondary plots of the inhibitor concentration [I] versus *K*_m_^43,44^.

### Molecular docking

To perform the molecular docking studies, the Maestro Molecular Modeling platform (version 10.5) by Schrödinger, L.L.C. was used. The homology model structure of a-glucosidase was obtained according to the previously reported procedure. The protein was then prepared using a protein preparation wizard. PROPKA assigned H-bonds at pH: 7.4. To prepare the ligands, the 2D structures of the ligands were drawn in ChemDraw (ver. 16) and converted into SDF files, which were used further by the ligprep module. Ligands were prepared by OPLS_2005 force field using EPIK. The grid box was generated for each binding site using entries with a box size of 25 A, all derivatives were docked on binding sites using induced-fit docking, reporting 10 poses per ligand to form the final complex.

### Molecular dynamics simulations

MD simulations were conducted using the desmond operator of Schrodingers suit maestro. To build the system for MD simulation, the protein–ligand complexes were solvated with SPC explicit water molecules and placed in the center of an orthorhombic box in the periodic boundary condition^42^. The system’s charge was neutralized by adding Na^+^ and Cl^-^ to simulate the real cellular ionic concentrations. The MD simulations protocol involved minimization, pre-production, and finally production MD simulation steps. In the minimization procedure, the entire system was allowed to relax for 2500 steps by the steepest descent approach. Then the temperature of the system was raised from 0 to 300 K with a small force constant on the enzyme to restrict any drastic changes. MD simulations were performed via NPT (constant number of atoms; constant pressure, i.e., 1.01325 bar; and constant temperature, i.e., 300 K) ensemble. The Nose–Hoover chain method was used as the default thermostat with 1.0 ps interval and Martyna-Tobias-Klein as the default barostat with 2.0 ps interval by applying an isotropic coupling style. Long-range electrostatic forces were calculated based on the particle-mesh-based Ewald approach with the cutoff radius for Columbia forces set to 9.0 Å. Finally, the system was subjected to produce MD simulations for each protein–ligand complex. The dynamic behavior and structural changes of the systems were analyzed by the calculation of the RMSD and RMSF^45^.

### In silico pharmacokinetic properties of synthesized compounds

Prediction of the molecular properties of the synthesized compounds was performed using the online servers such as SwissADME and pkCSM so that the structure of each molecule were uploaded and the physicochemical and drug-likeness properties were reported.

## Supplementary Information

Supplementary Information 1.

Supplementary Information 2.

## Data Availability

All data generated or analyzed during this study are included in this published article and its supplementary information file.
