# Supplementary material for: Synthesis and structure–activity relationship studies of benzimidazole-thioquinoline derivatives as α-glucosidase inhibitors
Source: Sci Rep. 2023 Mar 16;13:4392. doi: 10.1038/s41598-023-31080-2 (PMC10020548; doi:10.1038/s41598-023-31080-2)
Supplement: Supplementary file 1 — Supplementary Information 1. [file 41598_2023_31080_MOESM1_ESM.pdf]

CHNS Analysis Report

Sample Set ID: CHNS-2022-09[120]-S11 Analysis Date: 1/12/2022

Institution: - Instrument Model: Eager 300 for EA1112

Supervisor:Student: Ms. Moghadam Farid

Sample Name	% C	% H	% N	% S
**6a**	75.18	4.66	11.44	-
**6b**	71.78	4.24	19.82	-
**6c**	71.58	4.09	10.98	-
**6d**	71.67	4.18	10.90	-
**6e**	68.67	3.91	10.35	-
**6f**	68.65	4.08	10.40	-
**6g**	68.65	4.08	10.39	-
**6h**	61.80	3.51	9.52	-
**6i**	61.78	3.58	9.55	-
**6j**	61.77	3.54	9.49	-
**6k**	63.26	3.38	9.69	-
**6l**	75.47	5.11	10.92	-
**6m**	75.80	5.18	11.21	-
**6n**	75.74	5.09	11.17	-
**6o**	66.97	3.91	13.58	-
**6p**	72.47	4.74	10.65	-
**6q**	70.69	4.86	13.85	-
