# Supplementary material for: Synthesis and structure–activity relationship studies of benzimidazole-thioquinoline derivatives as α-glucosidase inhibitors
Source: Sci Rep. 2023 Mar 16;13:4392. doi: 10.1038/s41598-023-31080-2 (PMC10020548; doi:10.1038/s41598-023-31080-2)
Supplement: Supplementary file 2 — Supplementary Information 2. [file 41598_2023_31080_MOESM2_ESM.pdf]

Fig. S1. 3-(1H-benzo[d]imidazol-2-yl)-2-(benzylthio)quinolone (**6a**)

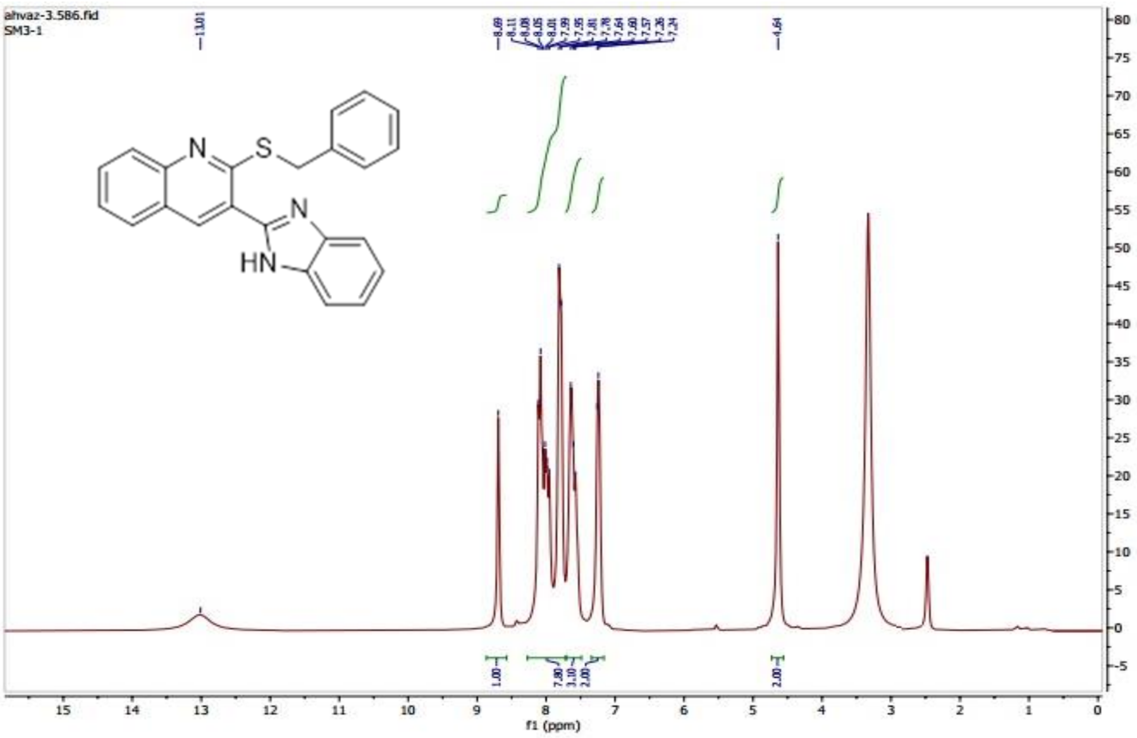

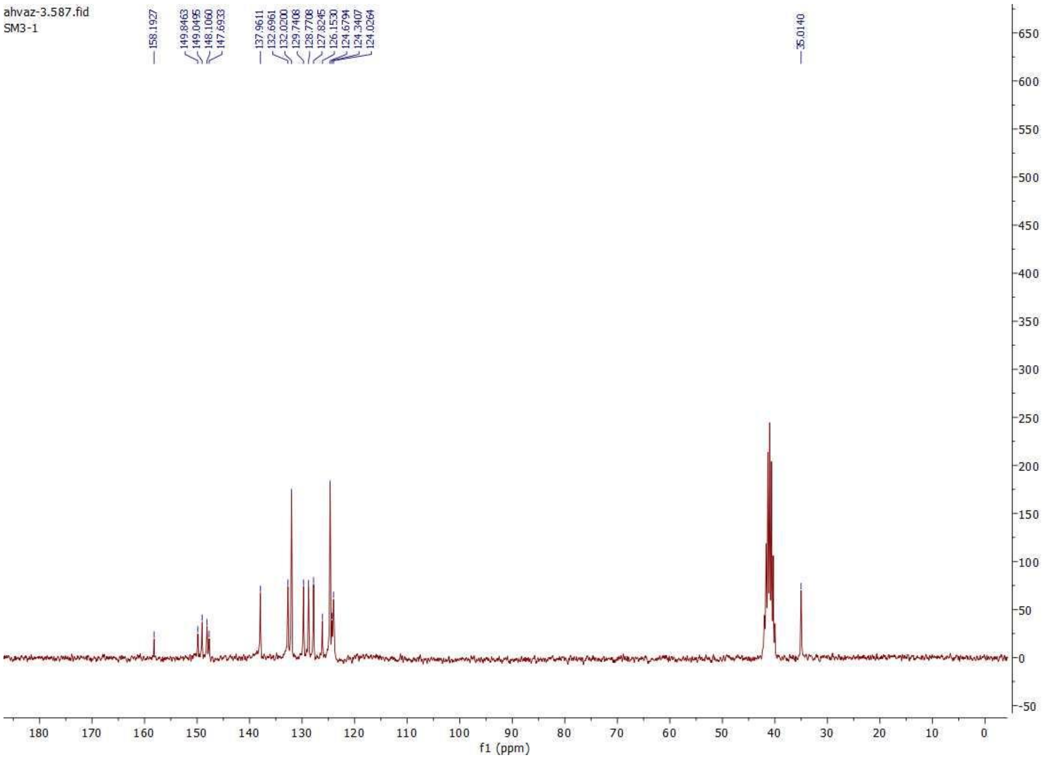
ahvaz-3.587.fid
SM3-1
158.1927
149.8463
149.0495
148.1060
147.6933
137.9611
132.6961
132.0200
129.7408
128.7708
127.8245
126.1530
124.6794
124.3407
124.0264
35.0140
f1 (ppm)

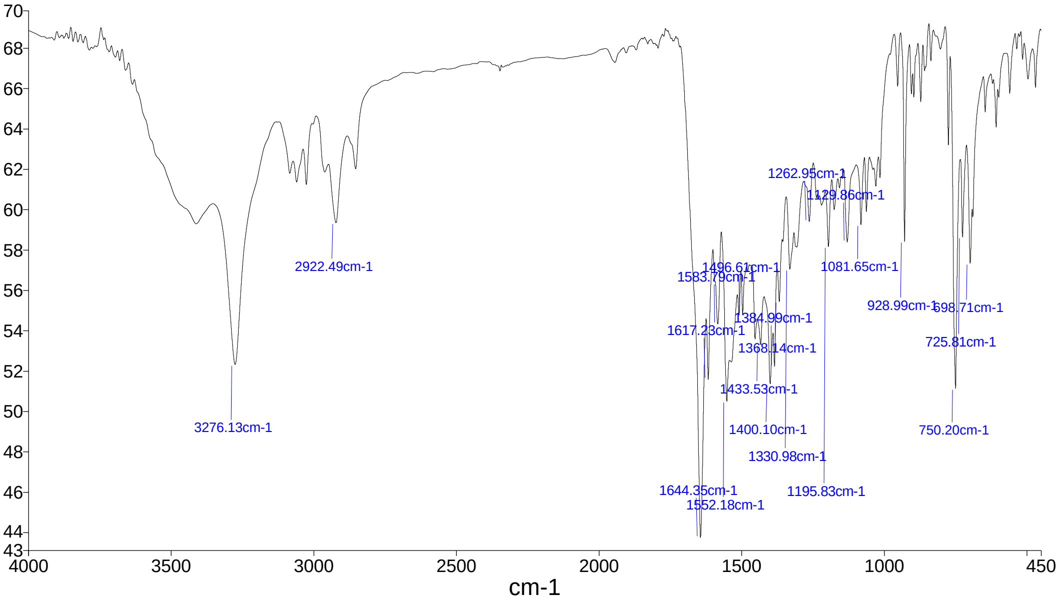
3276.13cm-1
2922.49cm-1
1644.35cm-1
1617.23cm-1
1583.79cm-1
1552.18cm-1
1496.61cm-1
1433.53cm-1
1400.10cm-1
1384.99cm-1
1368.14cm-1
1330.98cm-1
1262.95cm-1
1195.83cm-1
1129.86cm-1
1081.65cm-1
928.99cm-1
750.20cm-1
725.81cm-1
698.71cm-1
cm-1

Fig. S2.*3-(1H-benzo[d]imidazol-2-yl)-2-((2-fluorobenzyl)thio)quinolone (**6b**)*

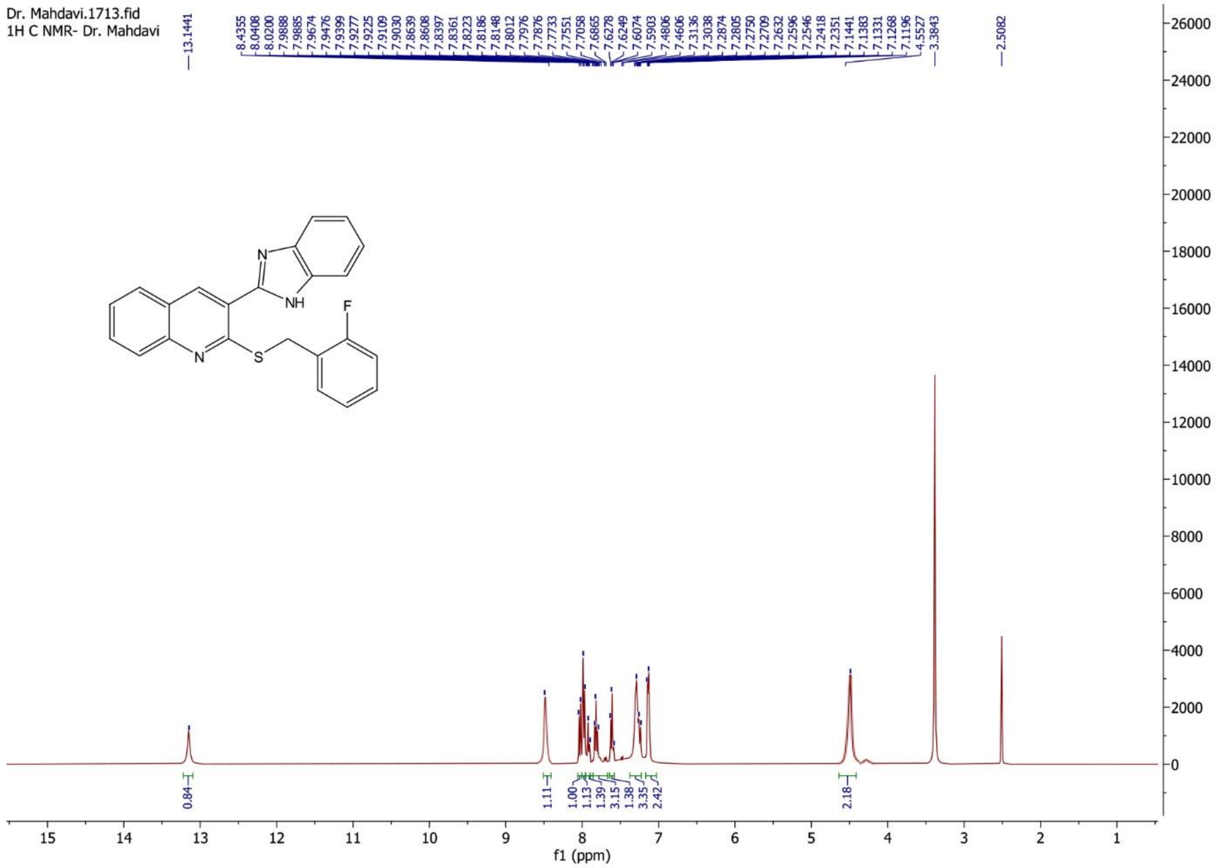

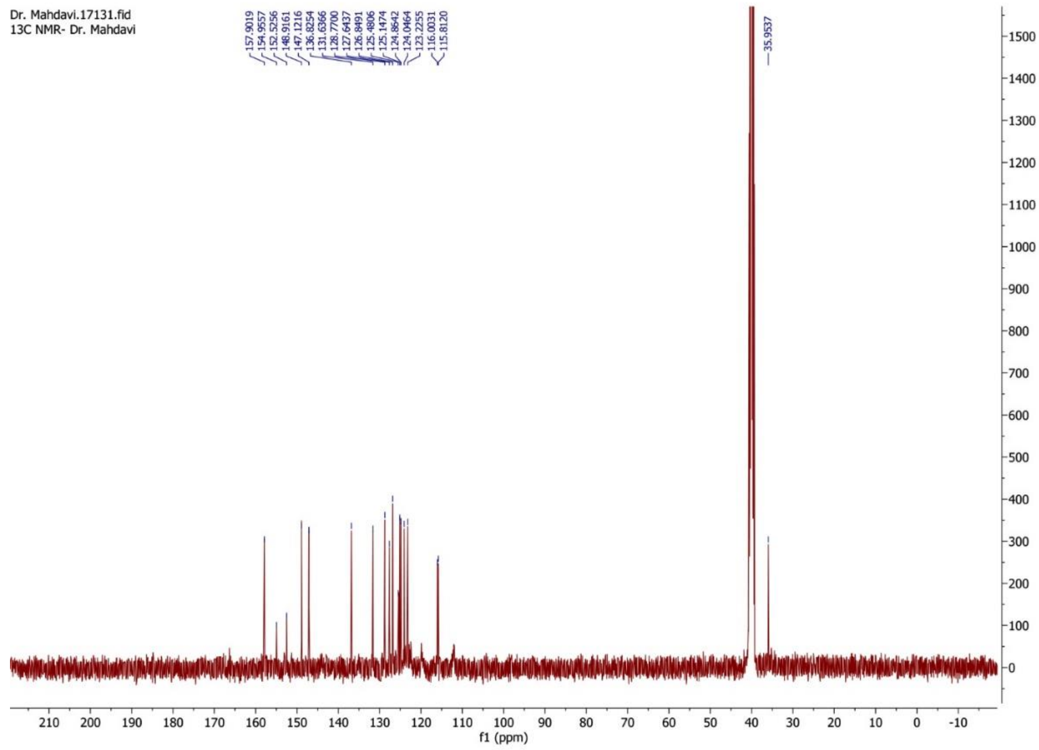

Dr. Mahdavi.17131.fid
13C NMR- Dr. Mahdavi
157.9019
154.9557
152.5256
148.9161
147.1216
136.8254
131.6366
128.7700
127.6437
126.8491
125.4806
125.1474
124.8642
124.0464
123.2255
116.0031
115.8120
35.9537
f1 (ppm)

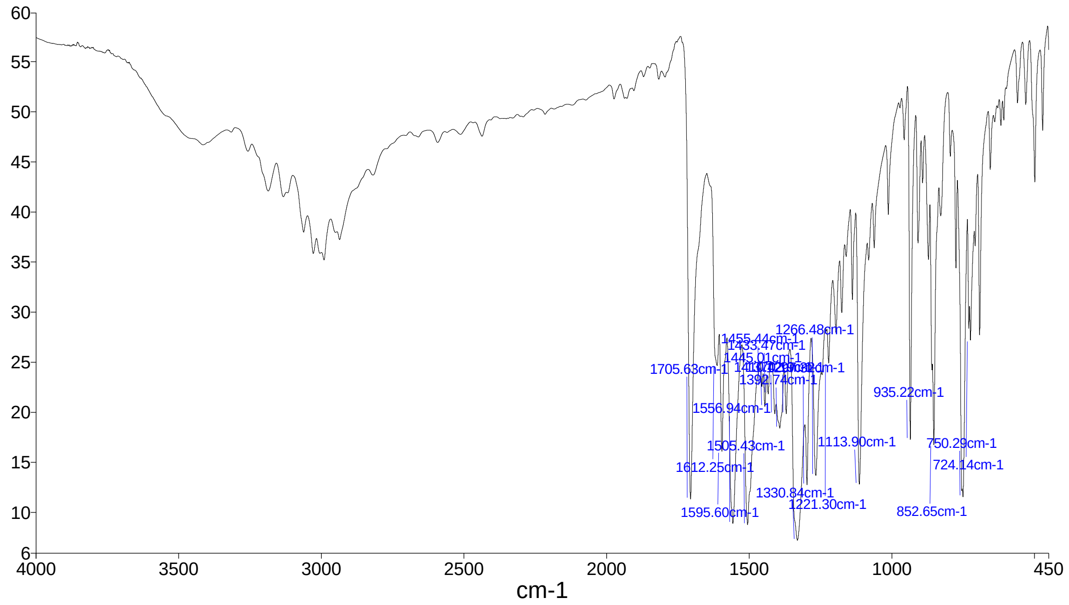

1705.63cm-1
1612.25cm-1
1595.60cm-1
1556.94cm-1
1505.43cm-1
1455.44cm-1
1433.47cm-1
1445.01cm-1
1392.74cm-1
1330.84cm-1
1266.48cm-1
1221.30cm-1
1113.90cm-1
935.22cm-1
852.65cm-1
750.29cm-1
724.14cm-1
cm-1

Fig. S3.*3-(1H-benzo[d]imidazol-2-yl)-2-((3-fluorobenzyl)thio)quinolone* (***6c***)

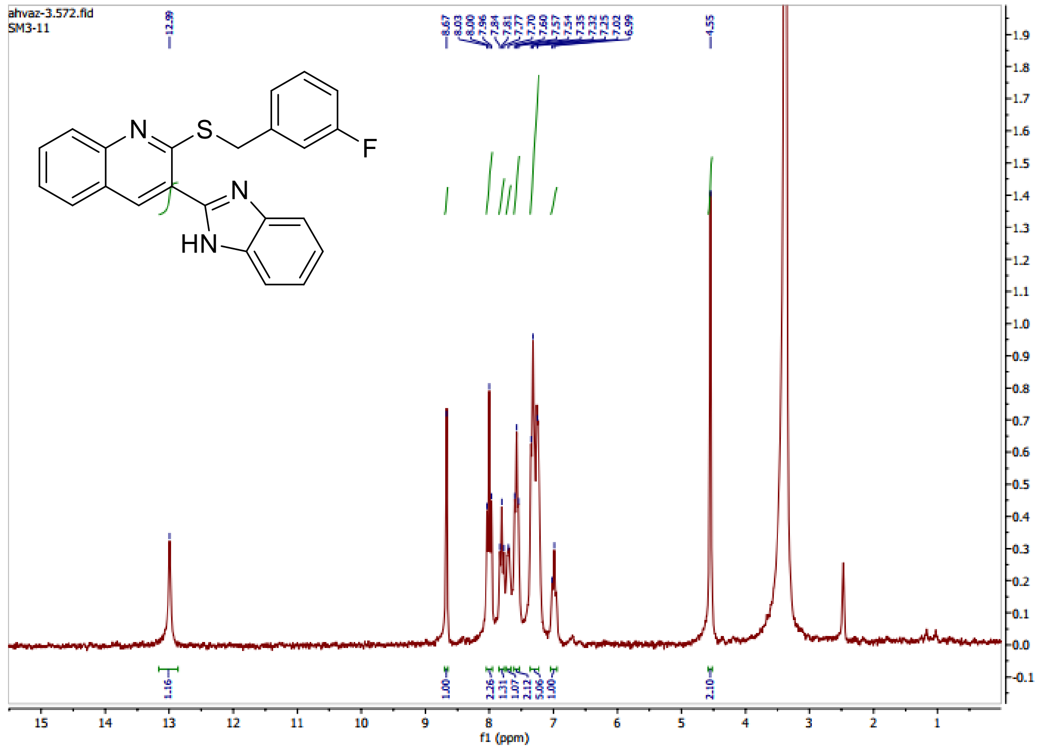

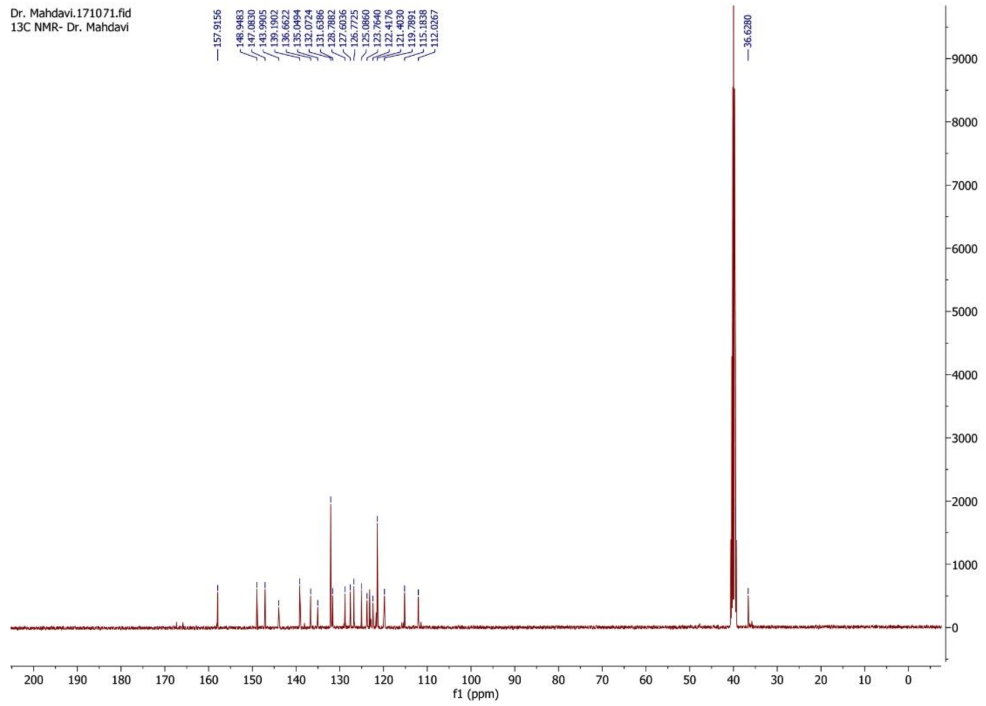
Dr. Mahdavi.171071.fid
13C NMR- Dr. Mahdavi
157.9156
148.9483
147.0830
143.9905
139.1902
136.6622
135.0494
132.0724
131.6386
128.7882
127.6036
126.7725
125.0860
123.7640
122.4176
121.4030
119.7891
115.1838
112.0267
36.6280
f1 (ppm)

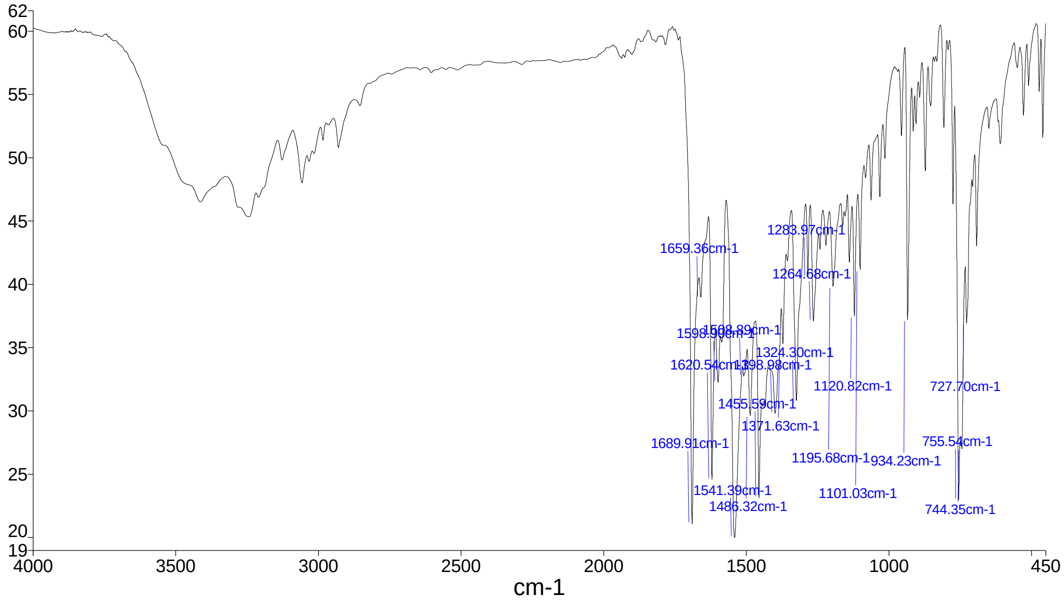
1659.36cm-1
1283.97cm-1
1264.68cm-1
1324.30cm-1
1620.54cm-1
1398.98cm-1
1120.82cm-1
727.70cm-1
1455.59cm-1
1371.63cm-1
1689.91cm-1
755.54cm-1
1195.68cm-1
934.23cm-1
1541.39cm-1
1486.32cm-1
1101.03cm-1
744.35cm-1
cm-1

Fig. S4. *3-(1H-benzo[d]imidazol-2-yl)-2-((4-fluorobenzyl)thio)quinolone (**6d**)*

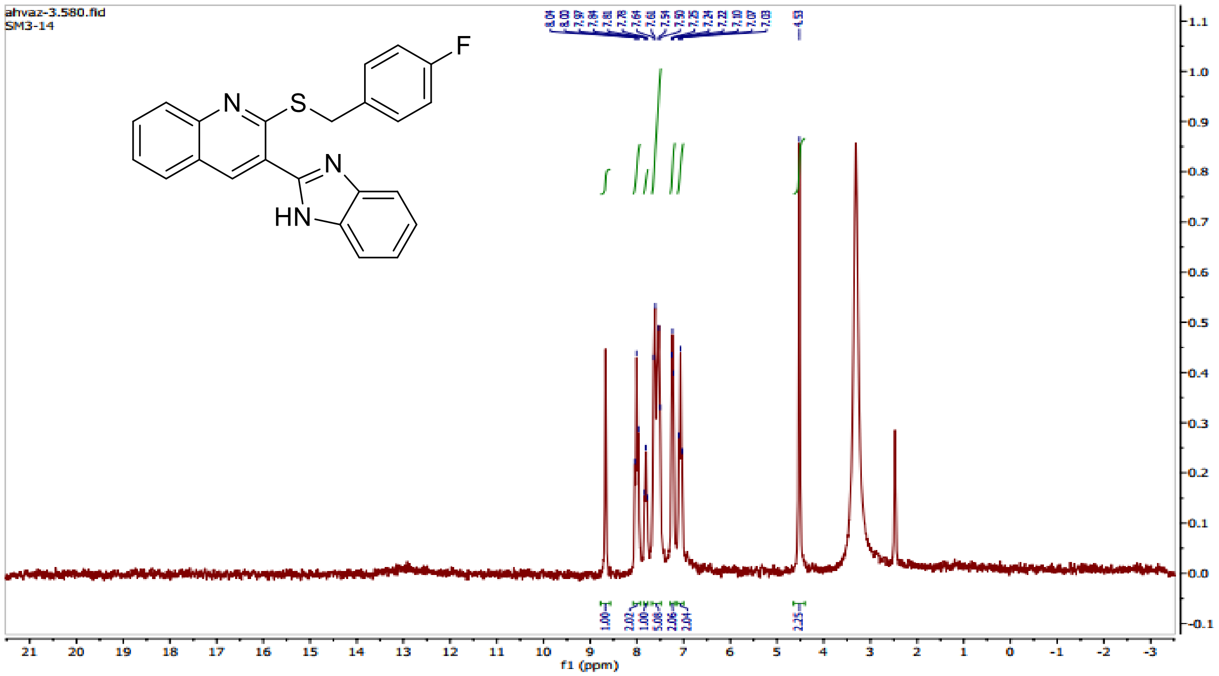

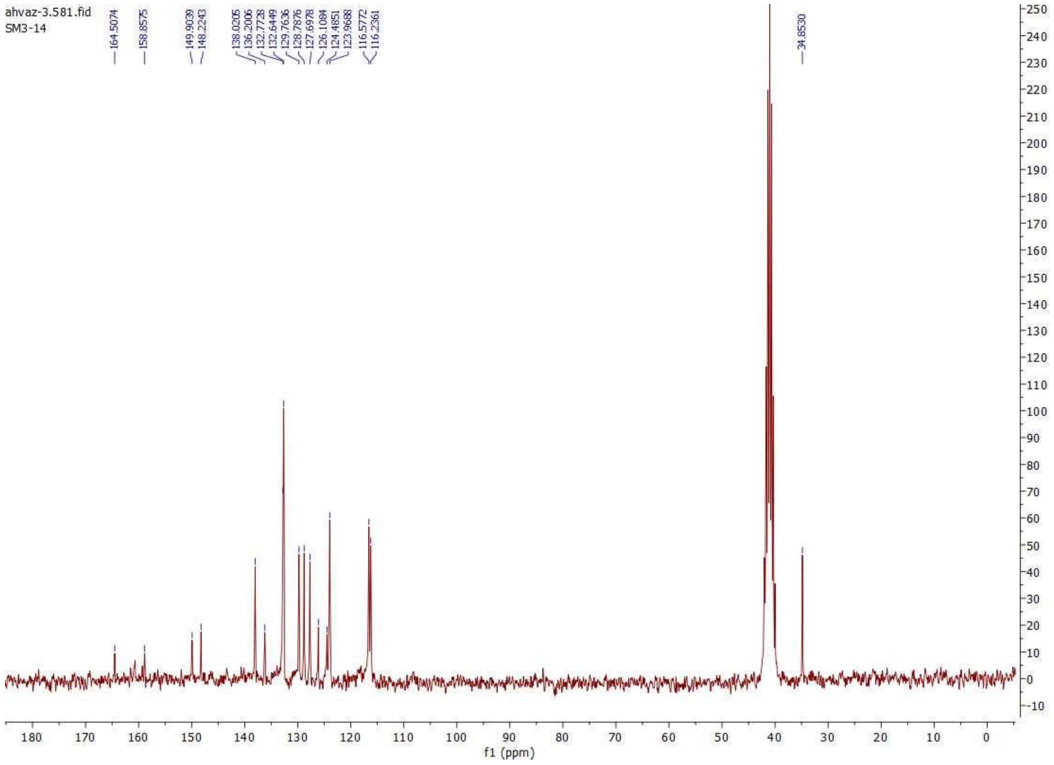
ahvaz-3.581.fid
SM3-14
164.5074
158.8575
149.9039
148.2243
138.0205
136.2006
132.7728
132.6449
129.7636
128.7876
127.6978
126.1084
124.4851
123.9688
116.5772
116.2361
34.8530
f1 (ppm)

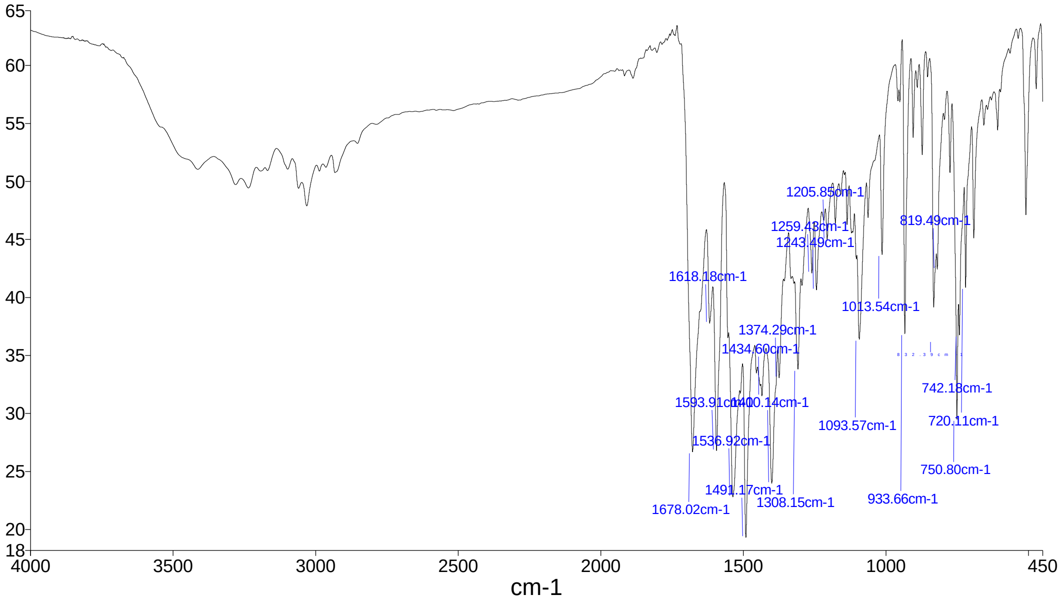
1205.85cm-1
1259.43cm-1
1243.49cm-1
819.49cm-1
1618.18cm-1
1013.54cm-1
1374.29cm-1
1434.60cm-1
742.18cm-1
1593.91cm-1
1400.14cm-1
720.11cm-1
1093.57cm-1
1536.92cm-1
750.80cm-1
1491.17cm-1
1308.15cm-1
933.66cm-1
1678.02cm-1
cm-1

Fig. S5. *3-(1H-benzo[d]imidazol-2-yl)-2-((2-chlorobenzyl)thio)quinolone (**6e**)*

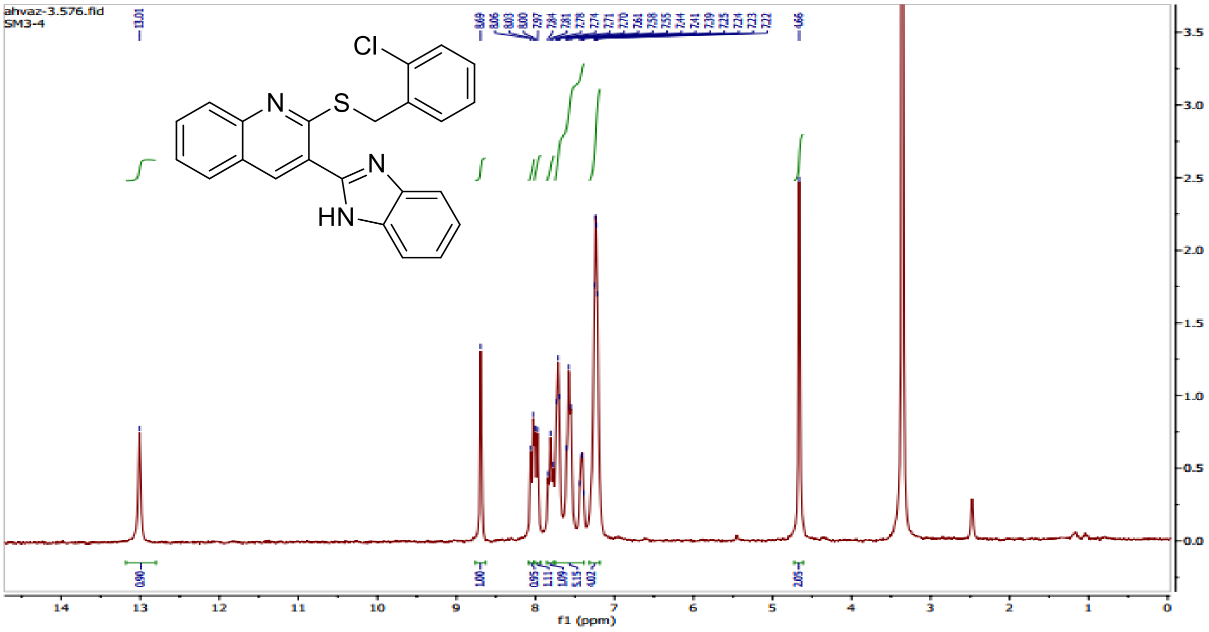

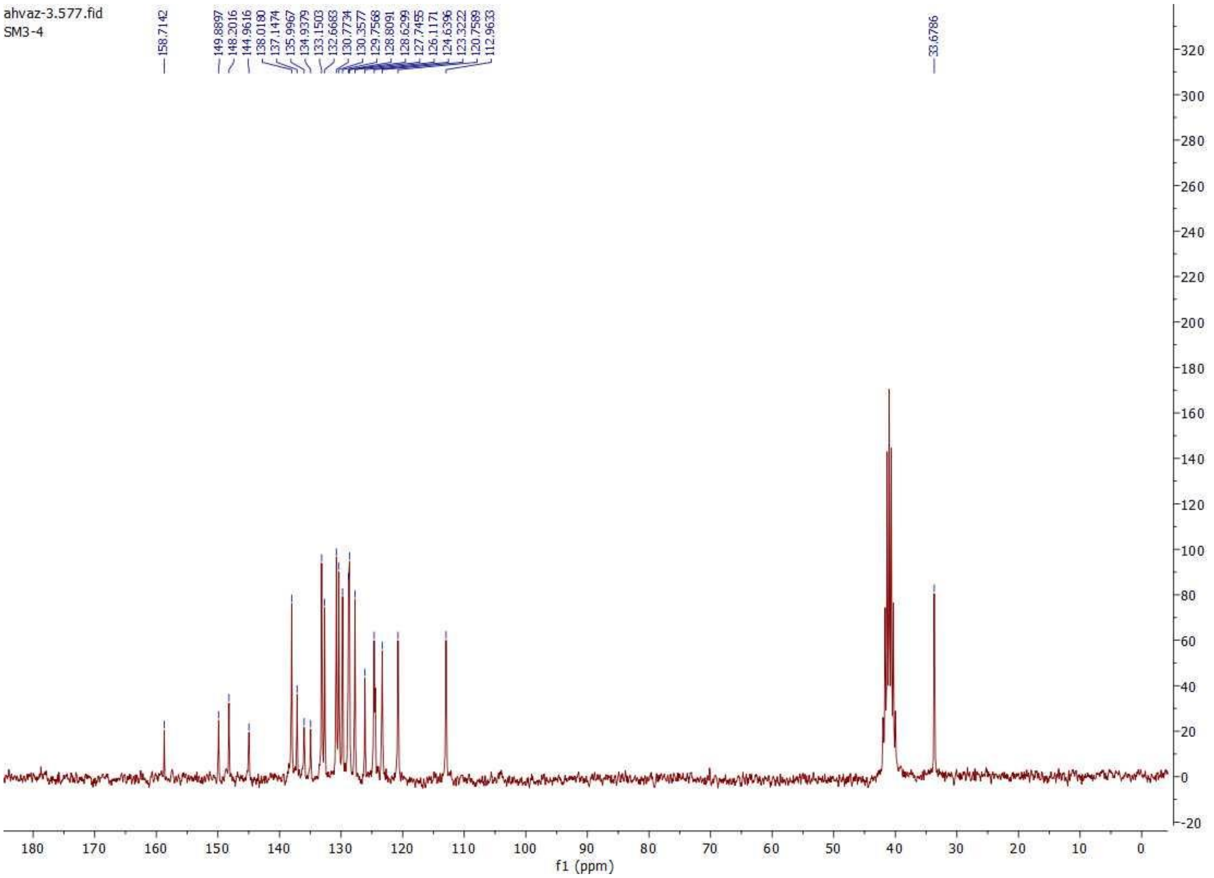
ahvaz-3.577.fid
SM3-4
158.7142
149.8897
148.2016
144.9616
138.0180
137.1474
135.9967
134.9379
133.1503
132.6683
130.7734
130.3577
129.7568
128.8091
128.6299
127.7455
126.1171
124.6396
123.3222
120.7589
112.9633
33.6786
f1 (ppm)

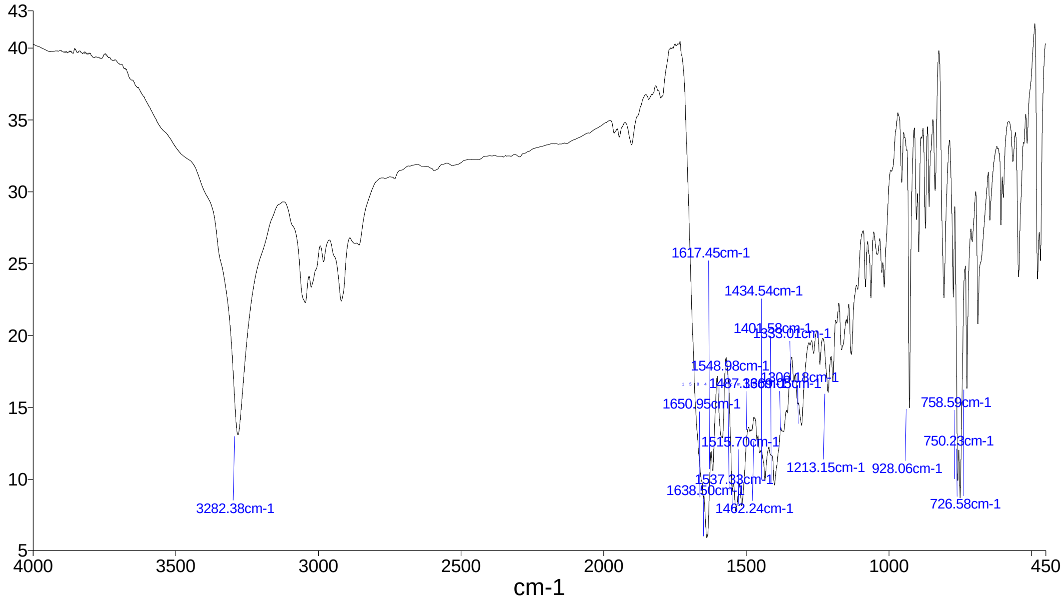
1617.45cm-1
1434.54cm-1
1548.98cm-1
1650.95cm-1
1515.70cm-1
1537.33cm-1
1638.50cm-1
1462.24cm-1
1213.15cm-1
928.06cm-1
758.59cm-1
750.23cm-1
726.58cm-1
3282.38cm-1
cm-1

Fig. S6. *3-(1H-benzo[d]imidazol-2-yl)-2-((3-chlorobenzyl)thio)quinolone* (***6f***)

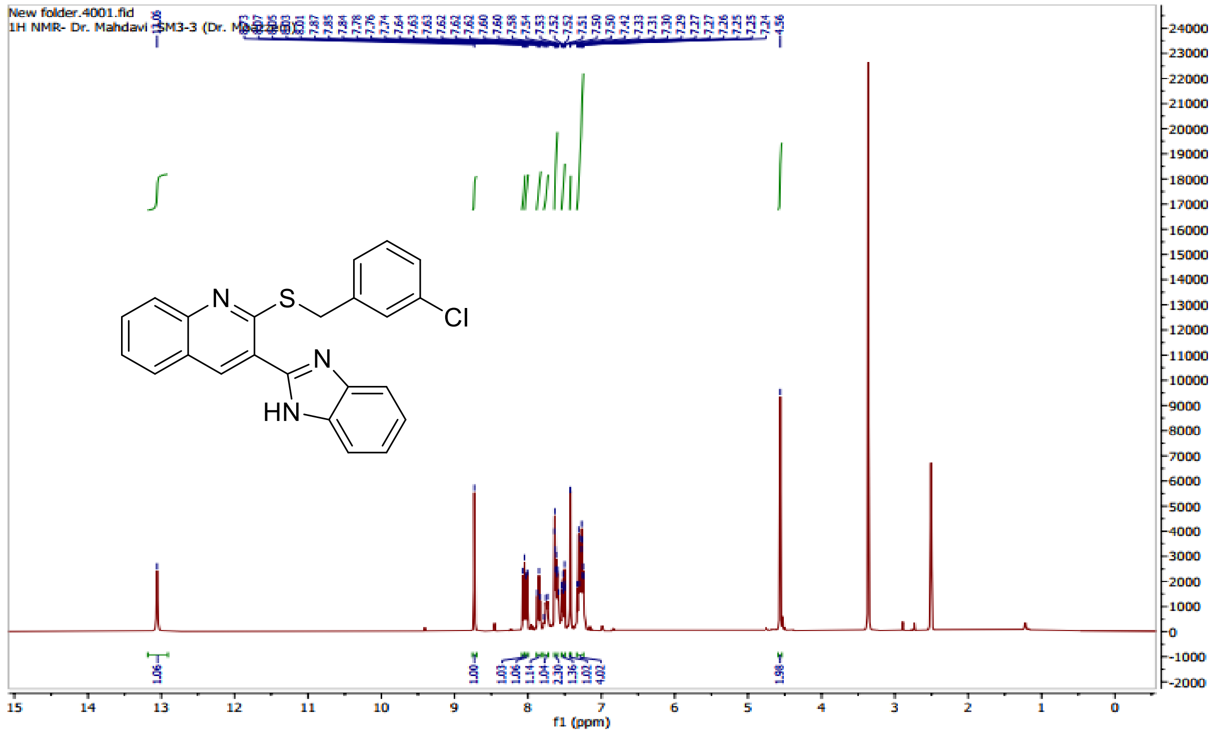

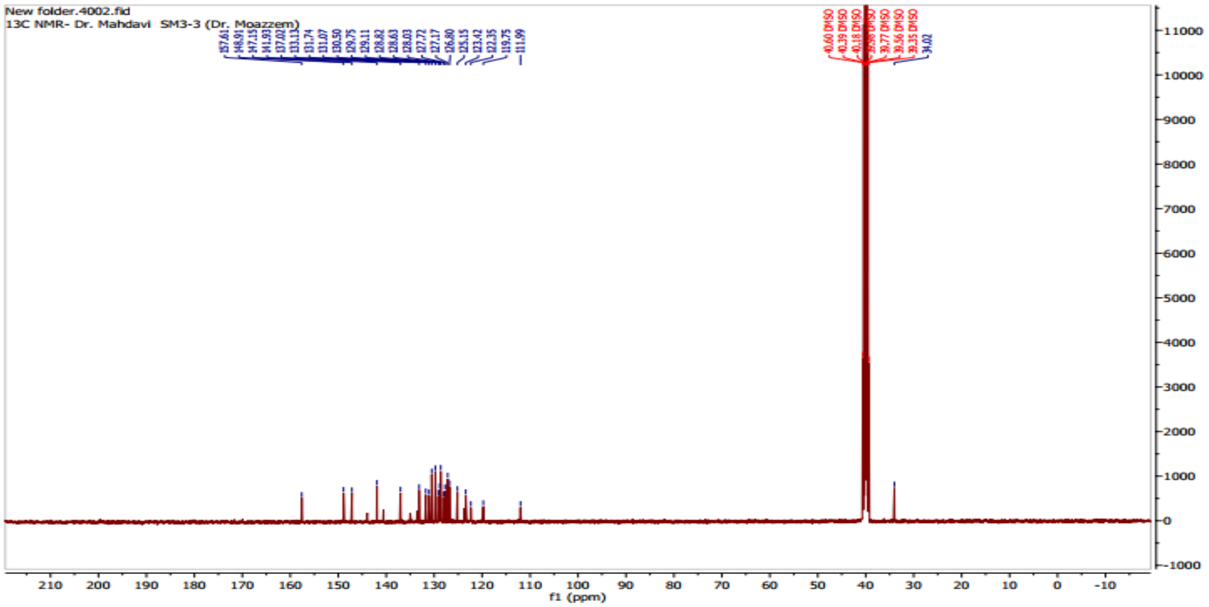
New folder.4002.fid
13C NMR- Dr. Mahdavi SM3-3 (Dr. Moazzem)
157.61
148.91
147.15
141.93
137.02
133.13
131.74
131.07
130.50
129.75
129.11
128.82
128.63
128.03
127.72
127.17
126.80
125.15
123.42
122.35
119.75
111.99
40.60 DMSO
40.39 DMSO
40.18 DMSO
39.98 DMSO
39.77 DMSO
39.56 DMSO
39.35 DMSO
34.02
f1 (ppm)

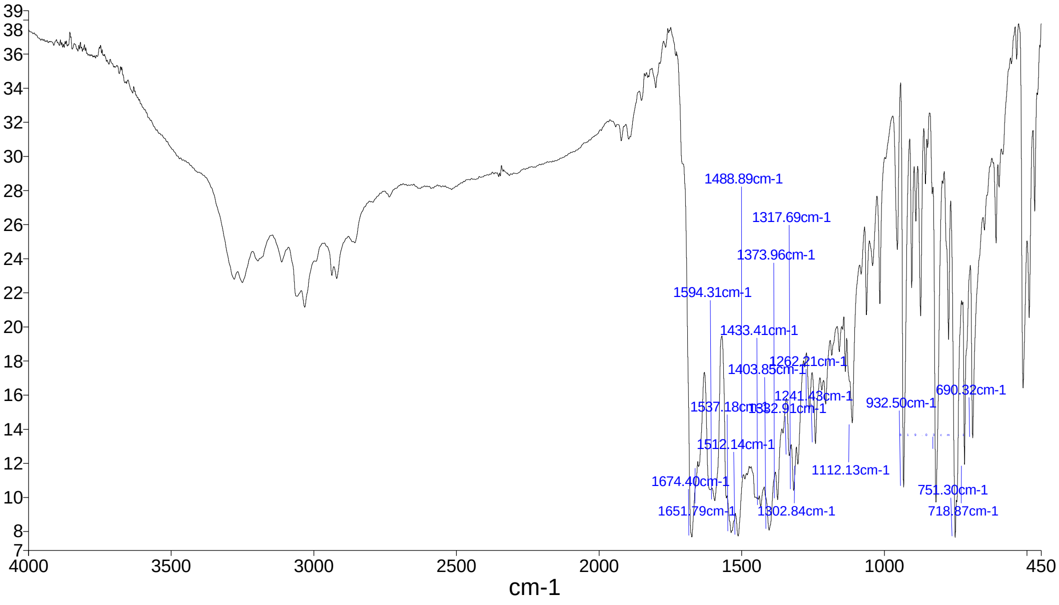
1488.89cm-1
1317.69cm-1
1373.96cm-1
1594.31cm-1
1433.41cm-1
1262.21cm-1
1403.85cm-1
1241.43cm-1
932.50cm-1
690.32cm-1
1537.18cm-1
1332.91cm-1
1512.14cm-1
1112.13cm-1
1674.40cm-1
751.30cm-1
1651.79cm-1
1302.84cm-1
718.87cm-1
cm-1

Fig. S7. *3-(1H-benzo[d]imidazol-2-yl)-2-((4-chlorobenzyl)thio)quinolone* ***(6g)***

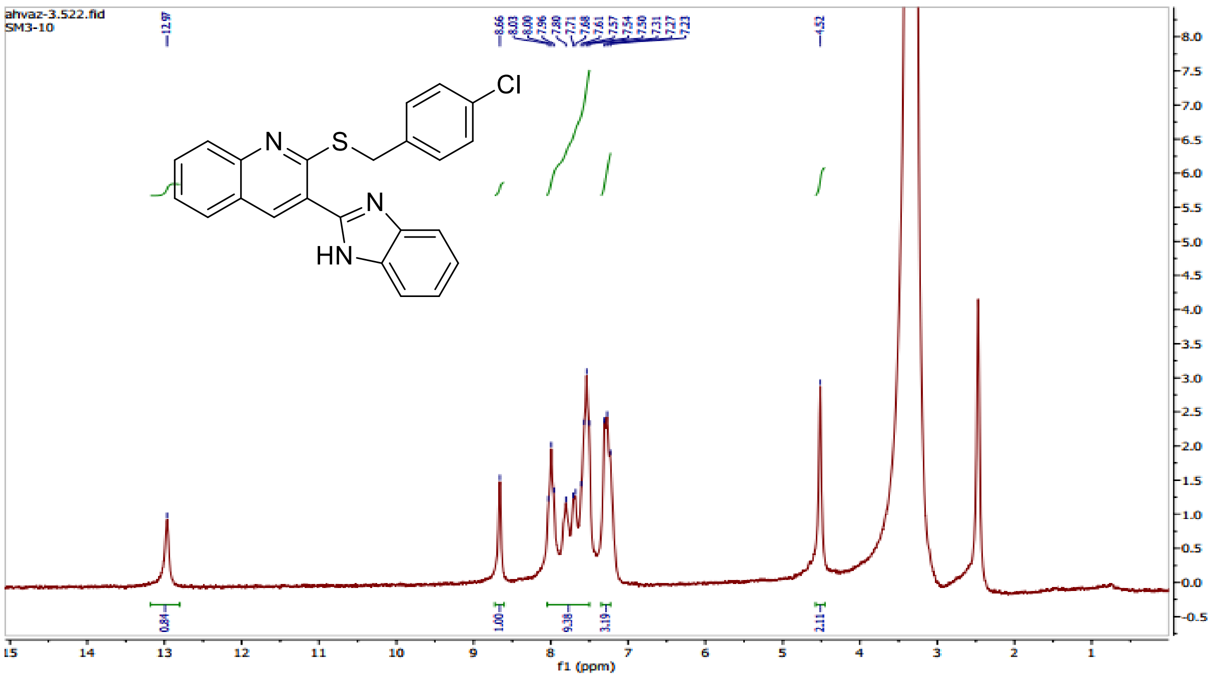

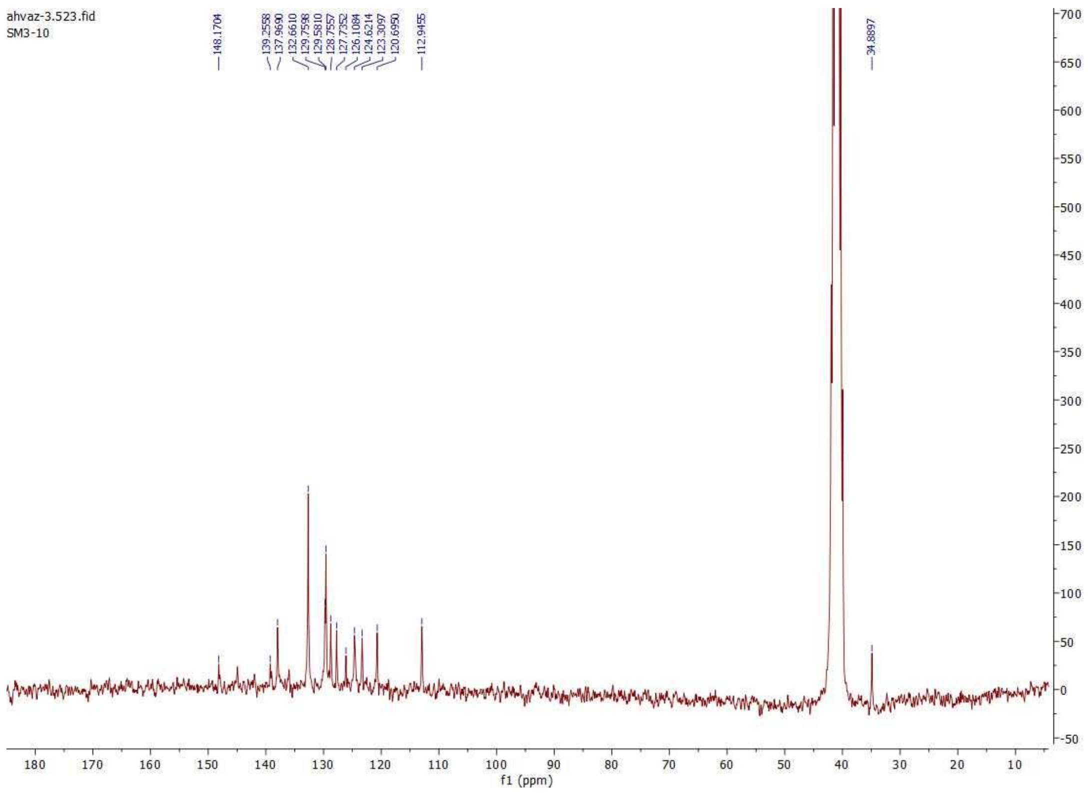
ahvaz-3.523.fid
SM3-10
148.1704
139.2558
137.9690
132.6610
129.7598
129.5810
128.7557
127.7352
126.1084
124.6214
123.3097
120.6950
112.9455
34.8897
f1 (ppm)

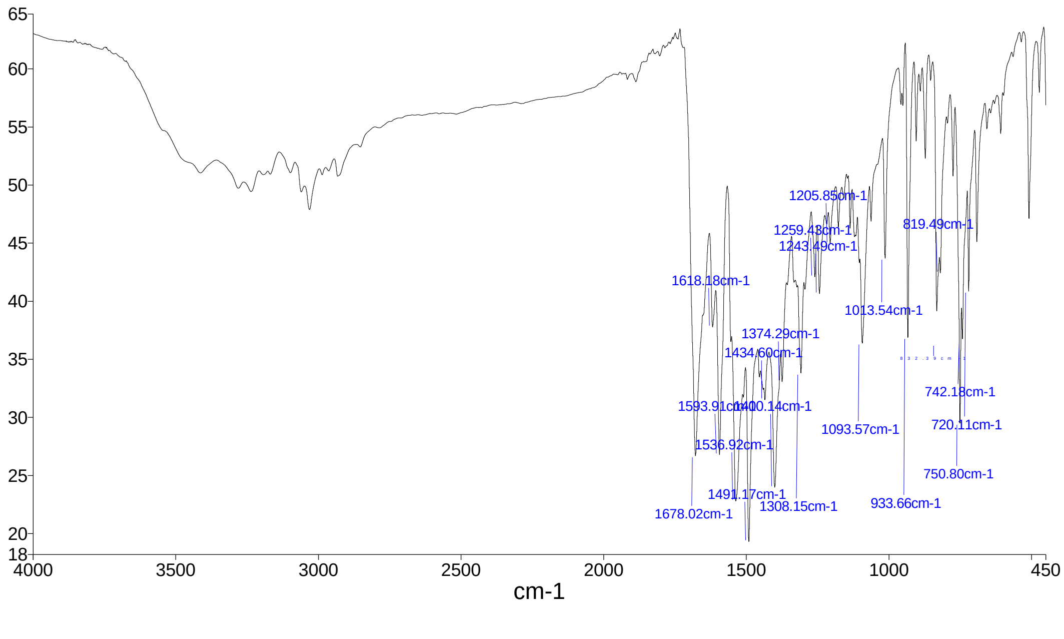
1205.85cm-1
1259.48cm-1
1243.49cm-1
819.49cm-1
1618.18cm-1
1013.54cm-1
1374.29cm-1
1434.60cm-1
742.18cm-1
1593.91cm-1
1400.14cm-1
720.11cm-1
1093.57cm-1
1536.92cm-1
750.80cm-1
1491.17cm-1
1308.15cm-1
933.66cm-1
1678.02cm-1
cm-1

Fig. S8. *3-(1H-benzo[d]imidazol-2-yl)-2-((2-bromobenzyl)thio)quinolone* ***(6h)***

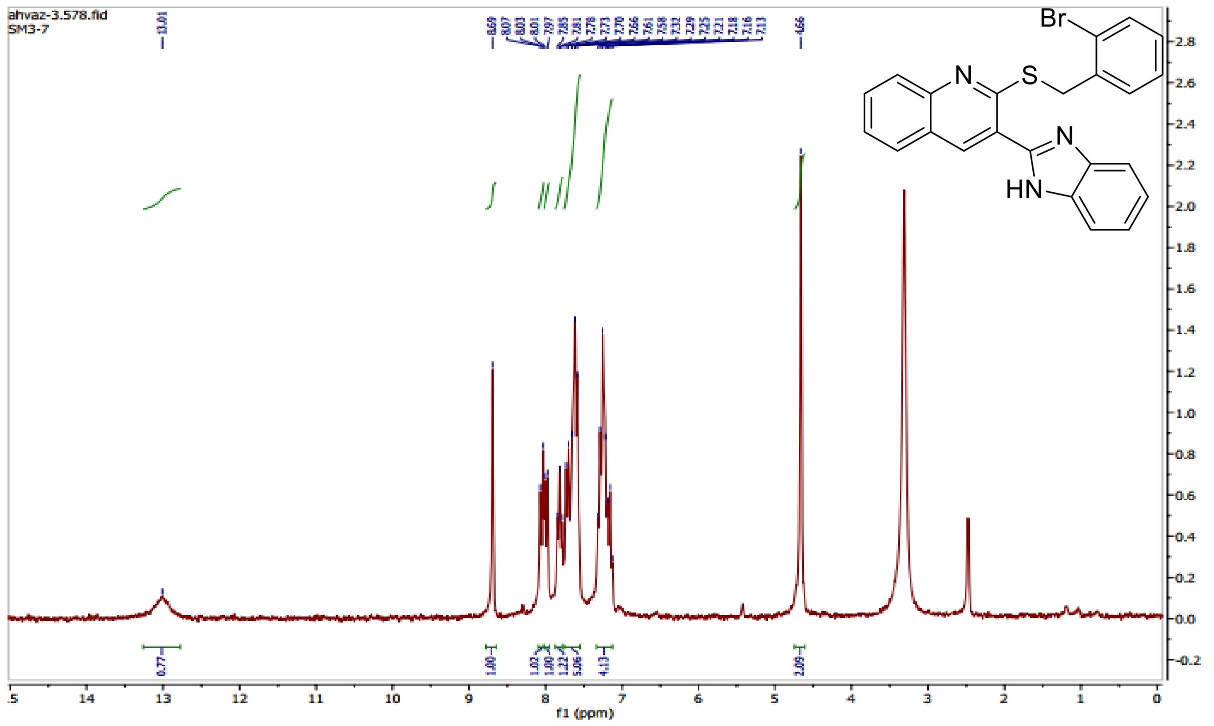

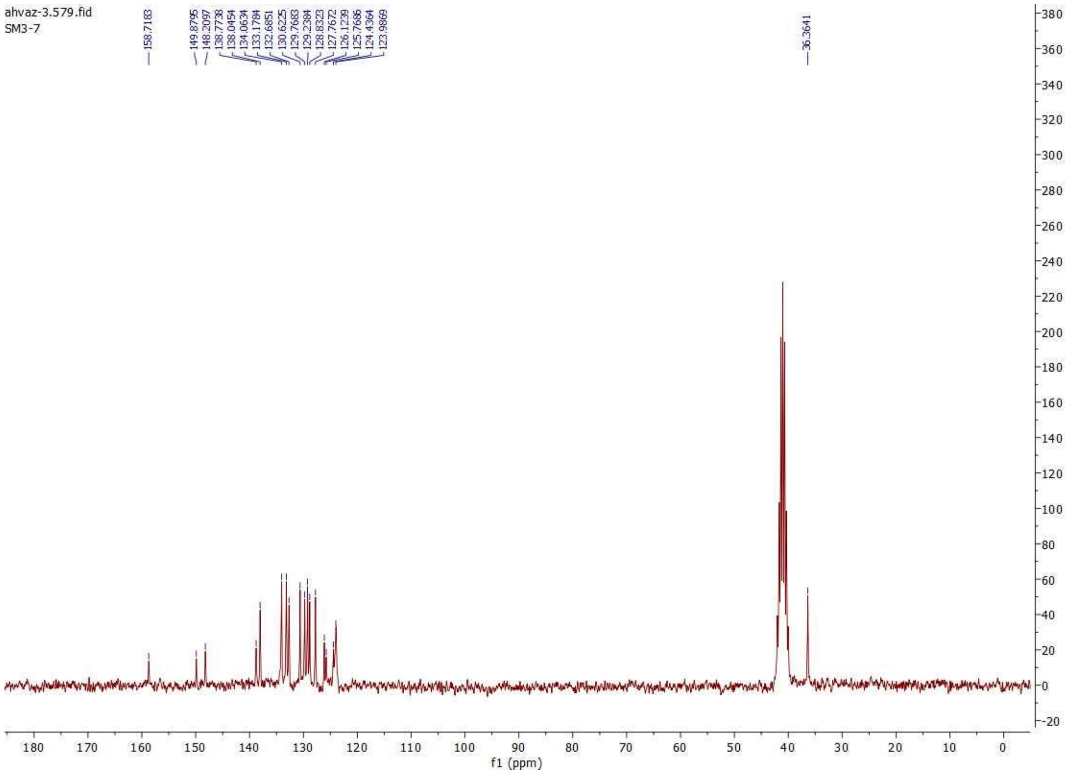
ahvaz-3.579.fid
SM3-7
158.7183
149.8795
148.2097
138.7738
138.0454
134.0634
133.1784
132.6851
130.6225
129.7683
129.2384
128.8323
127.7672
126.1239
125.7686
124.4364
123.9869
36.3641
f1 (ppm)

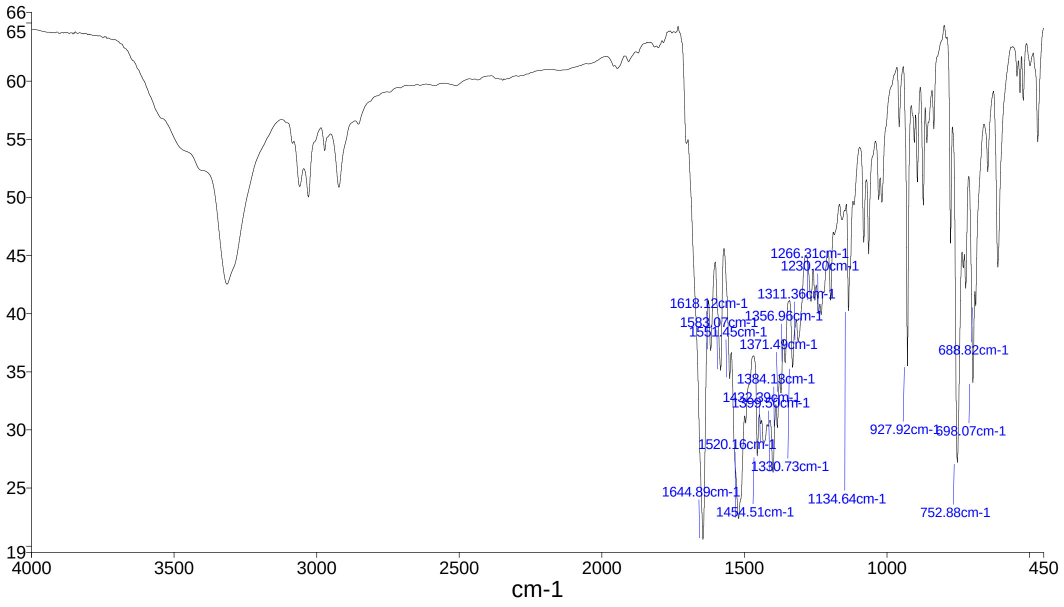
1266.31cm-1
1230.20cm-1
1311.36cm-1
1618.12cm-1
1356.96cm-1
1583.07cm-1
1551.45cm-1
1371.49cm-1
688.82cm-1
1384.18cm-1
1432.39cm-1
1399.50cm-1
927.92cm-1
698.07cm-1
1520.16cm-1
1330.73cm-1
1644.89cm-1
1134.64cm-1
1454.51cm-1
752.88cm-1
cm-1

Fig. S9. *3-(1H-benzo[d]imidazol-2-yl)-2-((3-bromobenzyl)thio)quinolone* (***6i***)

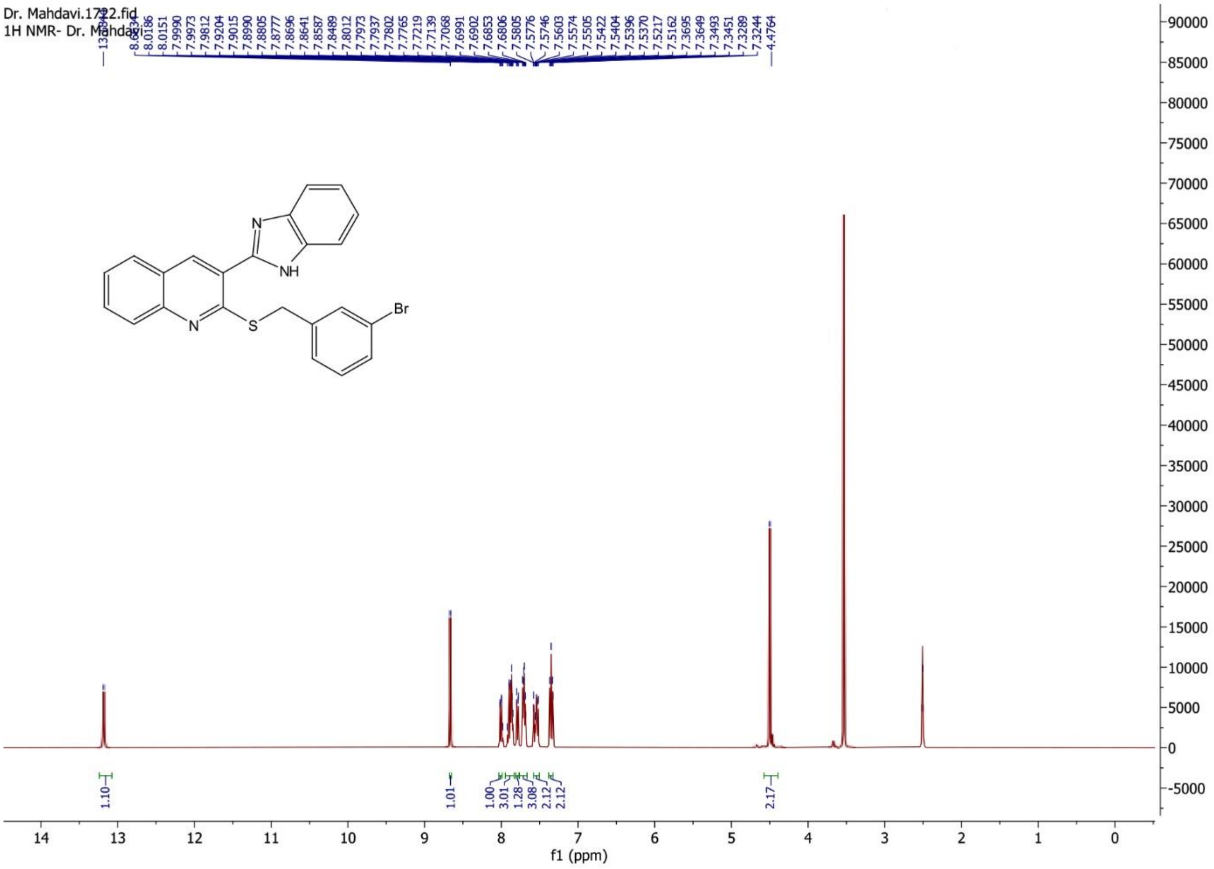

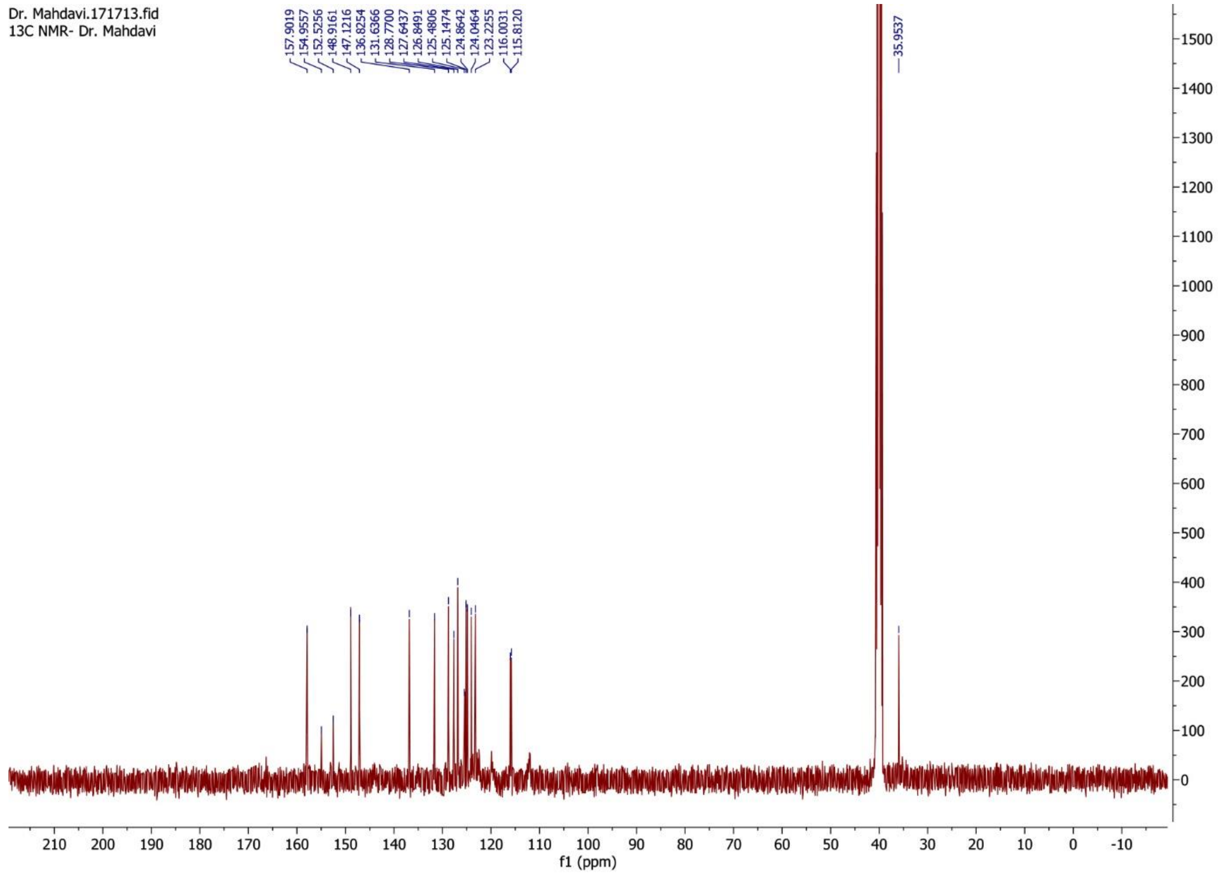

Dr. Mahdavi.171713.fid
13C NMR- Dr. Mahdavi
157.9019
154.9557
152.5256
148.9161
147.1216
136.8254
131.6366
128.7700
127.6437
126.8491
125.4806
125.1474
124.8642
124.0464
123.2255
116.0031
115.8120
35.9537
f1 (ppm)

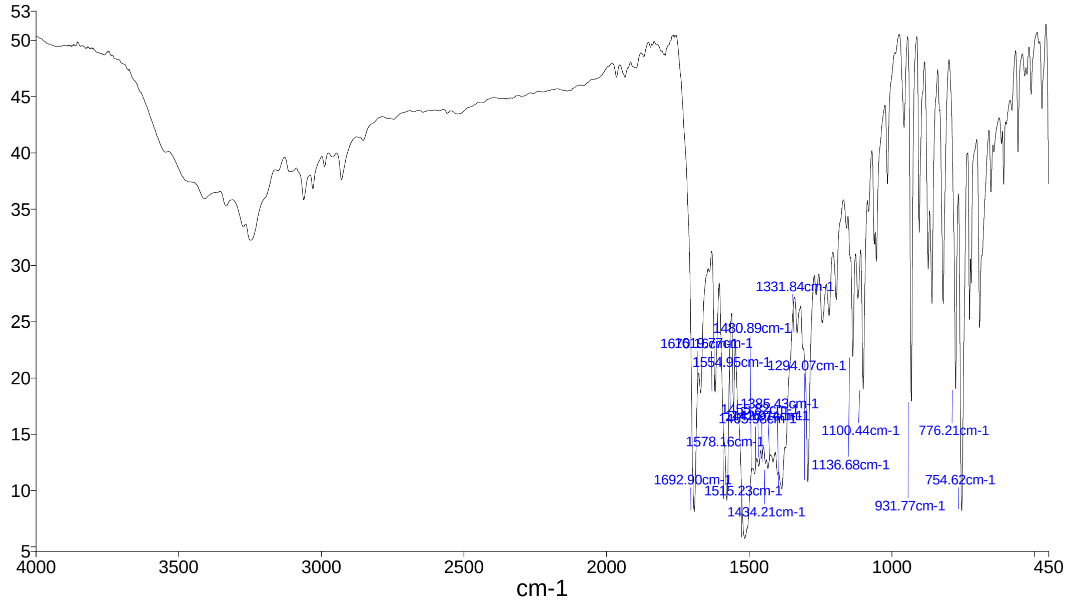

1331.84cm-1
1480.89cm-1
1554.95cm-1
1294.07cm-1
1385.43cm-1
1100.44cm-1
776.21cm-1
1578.16cm-1
1136.68cm-1
1692.90cm-1
754.62cm-1
1515.23cm-1
931.77cm-1
1434.21cm-1
cm-1

Fig. S10. *3-(1H-benzo[d]imidazol-2-yl)-2-((4-bromobenzyl)thio)quinolone (**6j**)*

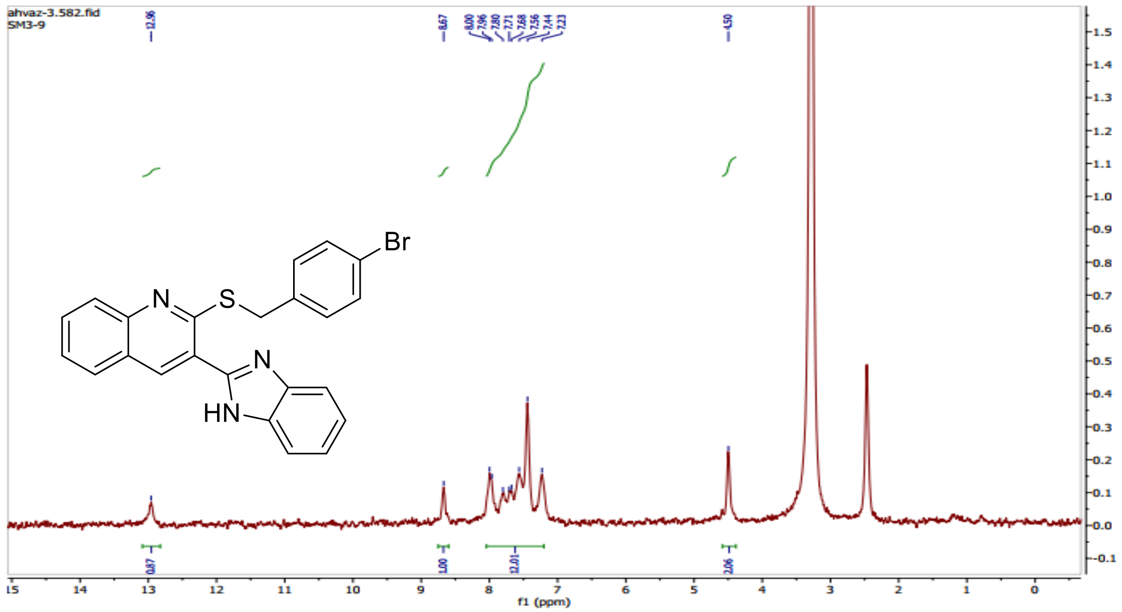

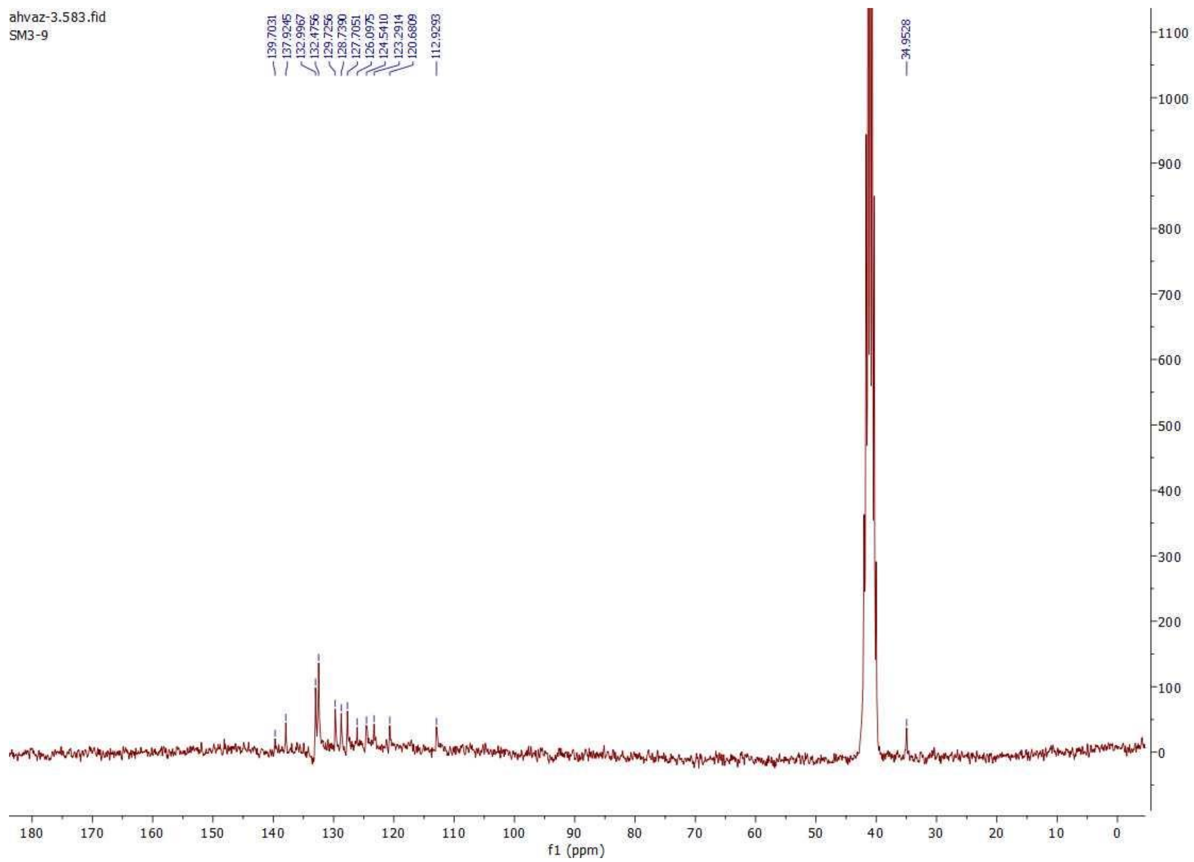
ahvaz-3.583.fid
SM3-9
139.7031
137.9245
132.9967
132.4756
129.7256
128.7390
127.7051
126.0975
124.5410
123.2914
120.6809
112.9293
34.9528
f1 (ppm)

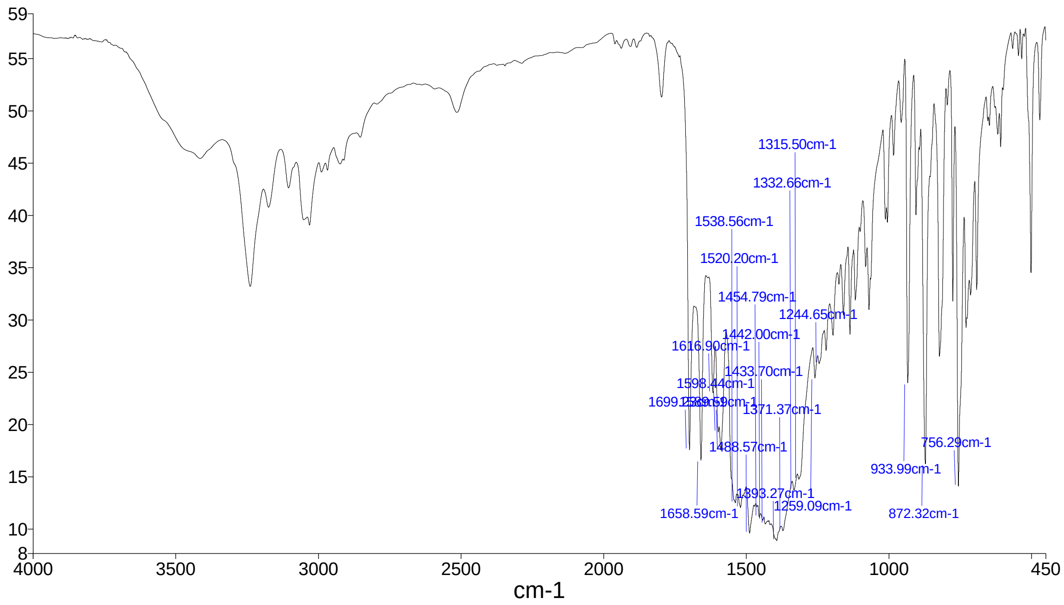
1315.50cm-1
1332.66cm-1
1538.56cm-1
1520.20cm-1
1454.79cm-1
1244.65cm-1
1442.00cm-1
1616.90cm-1
1433.70cm-1
1598.44cm-1
1371.37cm-1
1488.57cm-1
756.29cm-1
933.99cm-1
1393.27cm-1
1259.09cm-1
1658.59cm-1
872.32cm-1
cm-1

Fig. S11. *3-(1H-benzo[d]imidazol-2-yl)-2-((3,4-dichlorobenzyl)thio)quinolone* ***(6k)***

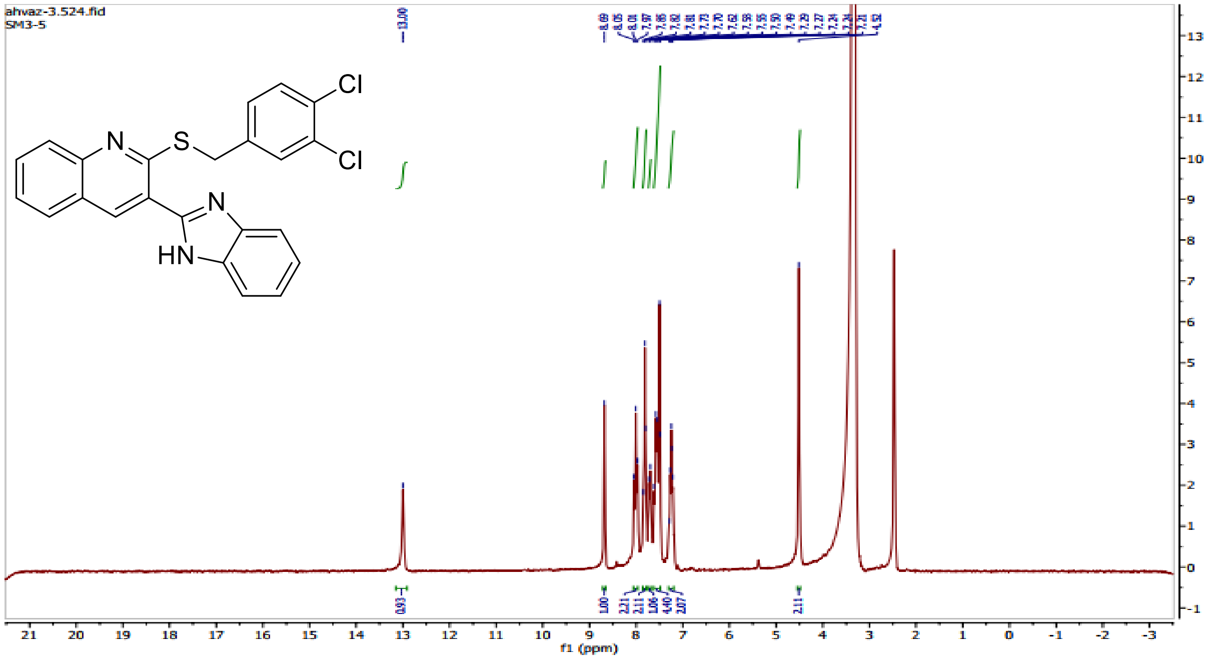

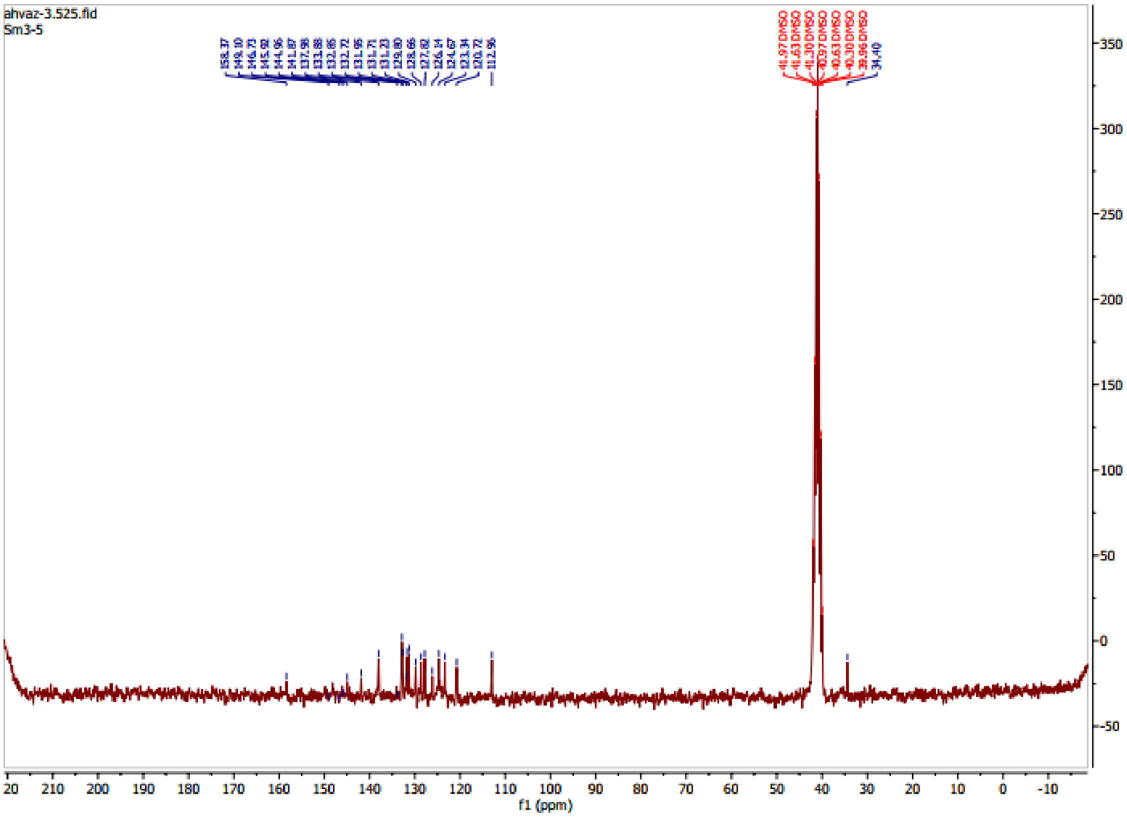
ahvaz-3.525.fid
Sm3-5
158.37
149.10
146.73
145.92
144.96
141.87
137.98
133.88
132.85
132.72
131.95
131.71
131.23
129.80
128.66
127.82
126.14
124.67
123.34
120.72
112.96
41.97 DMSO
41.63 DMSO
41.30 DMSO
40.97 DMSO
40.63 DMSO
40.30 DMSO
39.96 DMSO
34.40
f1 (ppm)

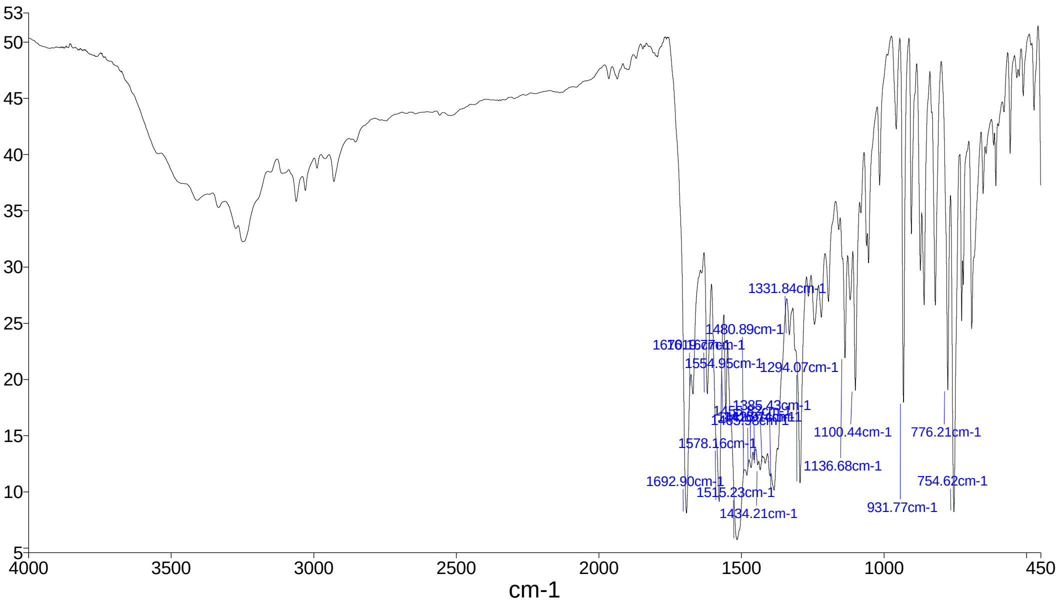
1331.84cm-1
1480.89cm-1
1554.95cm-1
1294.07cm-1
1385.43cm-1
1578.16cm-1
1100.44cm-1
776.21cm-1
1136.68cm-1
1692.90cm-1
754.62cm-1
1515.23cm-1
931.77cm-1
1434.21cm-1
cm-1

Fig. S12. *3-(1H-benzo[d]imidazol-2-yl)-2-((2-methylbenzyl)thio)quinolone (**6l**)*

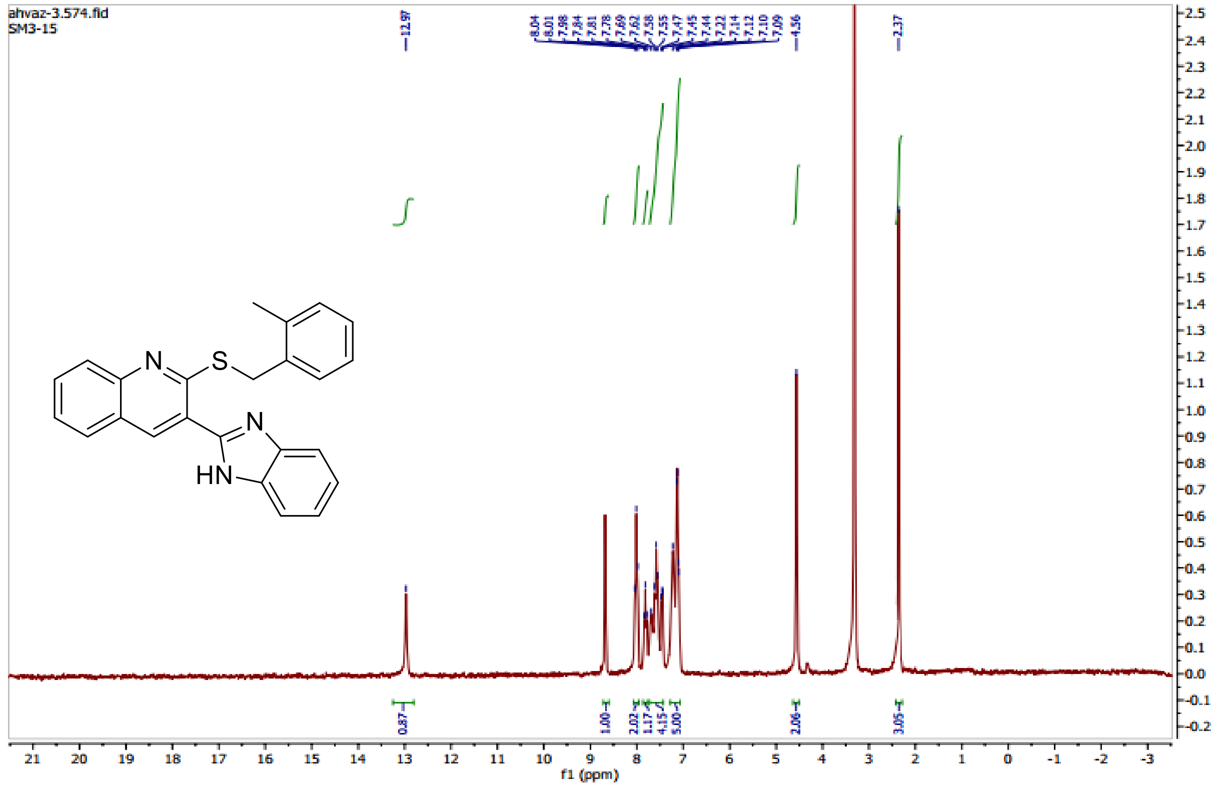

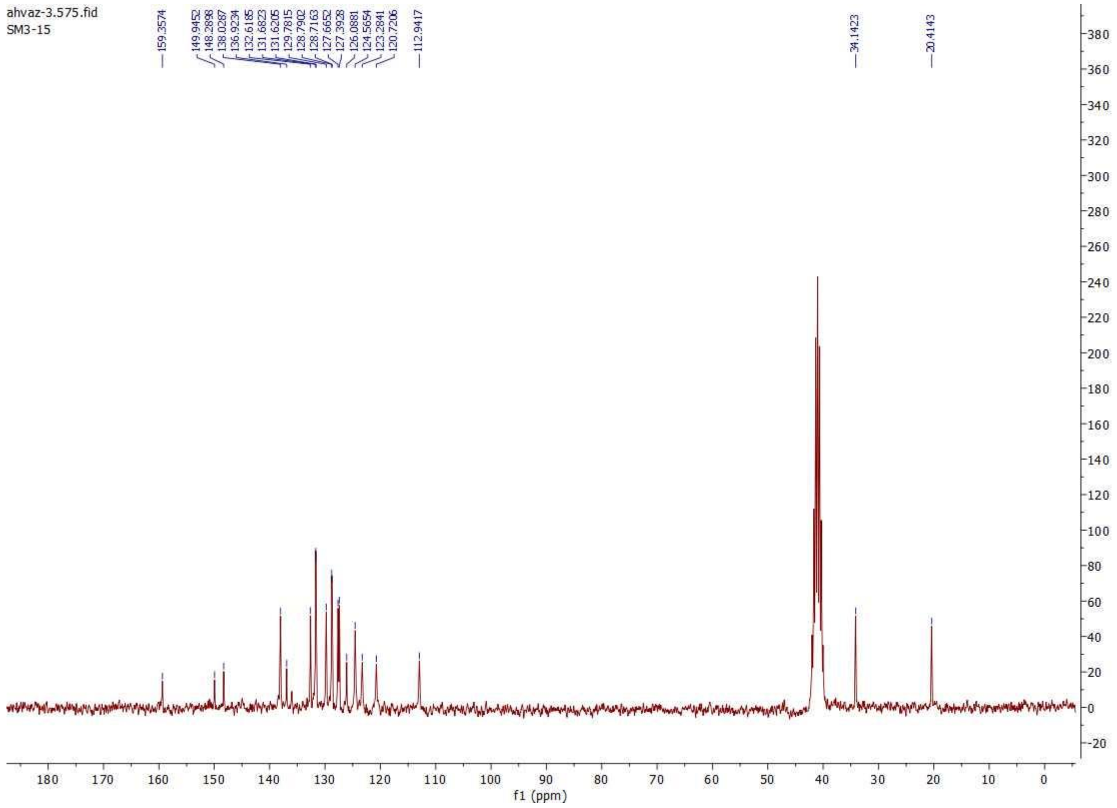

ahvaz-3.575.fid
SM3-15
159.3574
149.9452
148.2898
138.0287
136.9234
132.6185
131.6823
131.6205
129.7815
128.7902
128.7163
127.6652
127.3928
126.0881
124.5654
123.2841
120.7206
112.9417
34.1423
20.4143
380
360
340
320
300
280
260
240
220
200
180
160
140
120
100
80
60
40
20
0
-20
180 170 160 150 140 130 120 110 100 90 80 70 60 50 40 30 20 10 0
f1 (ppm)

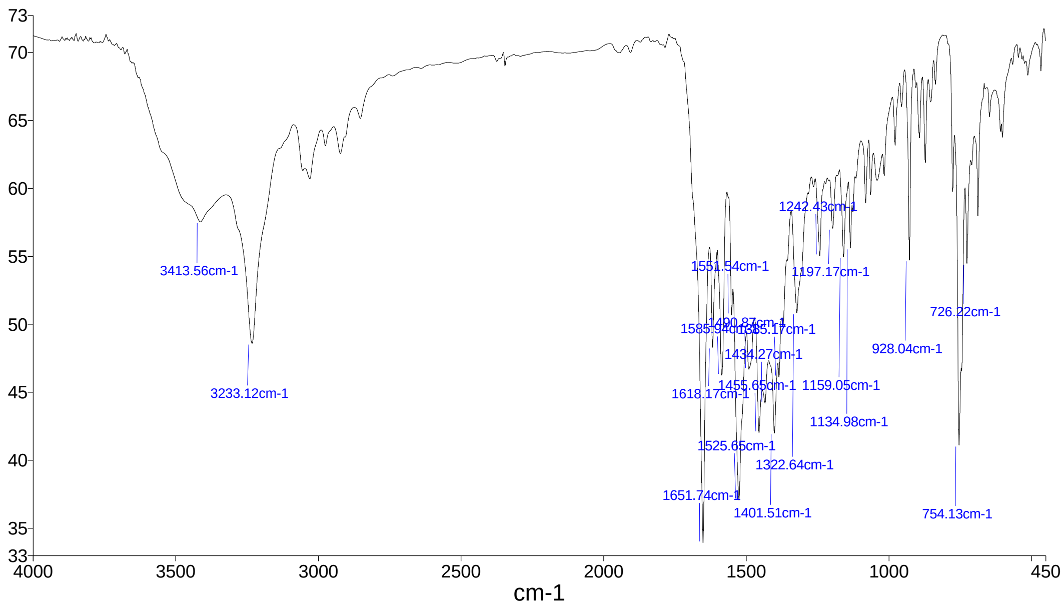

73
70
65
60
55
50
45
40
35
33
3413.56cm-1
3233.12cm-1
1242.43cm-1
1551.54cm-1
1197.17cm-1
1490.87cm-1
1585.94c
1385.17cm-1
1434.27cm-1
1455.65cm-1
1159.05cm-1
1618.17cm-1
1525.65cm-1
1322.64cm-1
1651.74cm-1
1401.51cm-1
1134.98cm-1
928.04cm-1
726.22cm-1
754.13cm-1
4000 3500 3000 2500 2000 1500 1000 450
cm-1

Fig.S13.*3-(1H-benzo[d]imidazol-2-yl)2-((3-methylbenzyl)thio)quinolone(***6m***)*

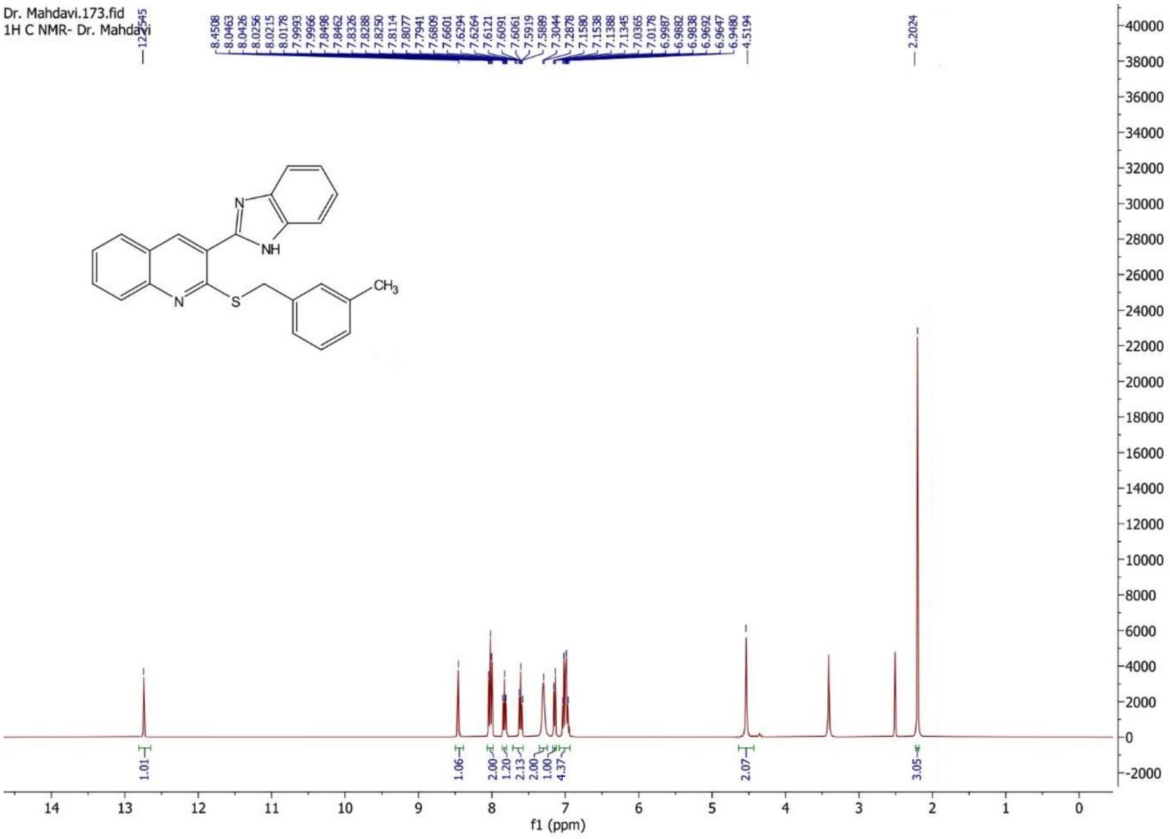

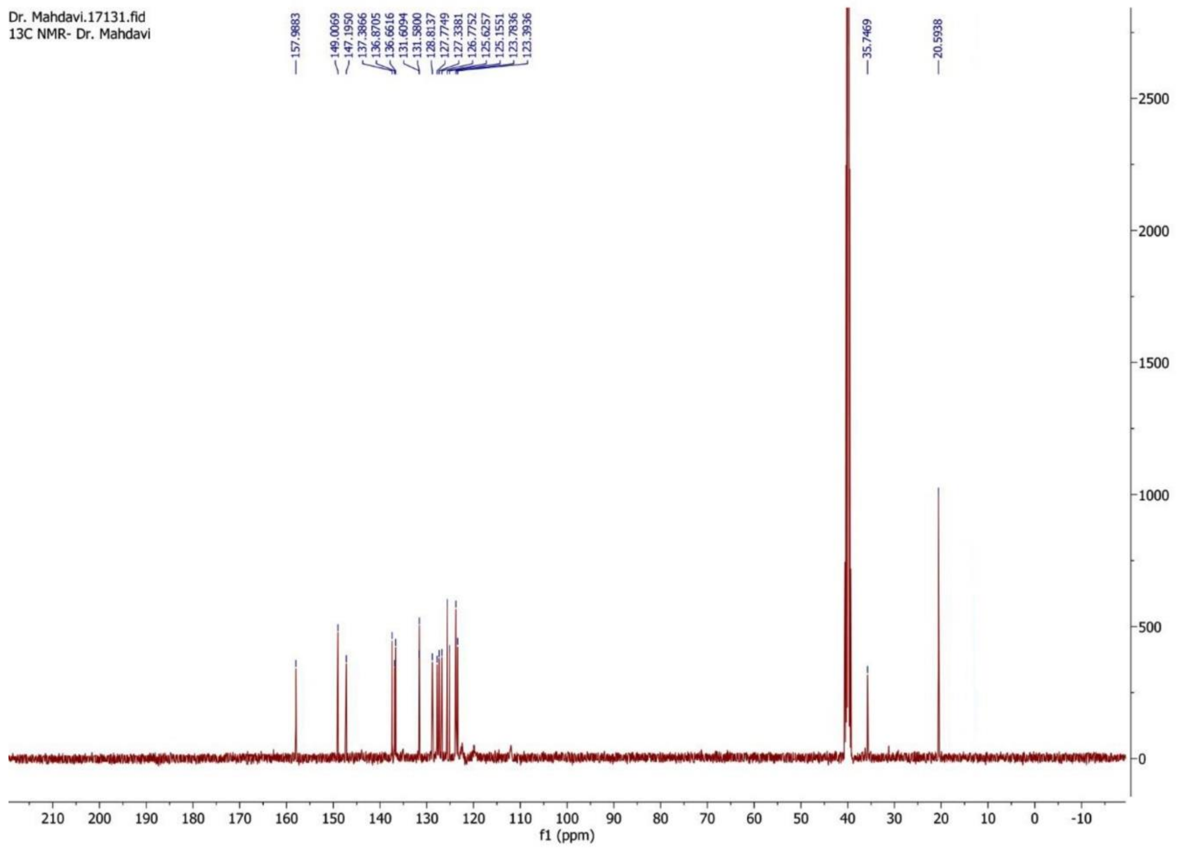
Dr. Mahdavi.17131.fid
13C NMR- Dr. Mahdavi
157.9883
149.0069
147.1950
137.3866
136.8705
136.6616
131.6094
131.5800
128.8137
127.7749
127.3381
126.7752
125.6257
125.1551
123.7836
123.3936
35.7469
20.5938
f1 (ppm)

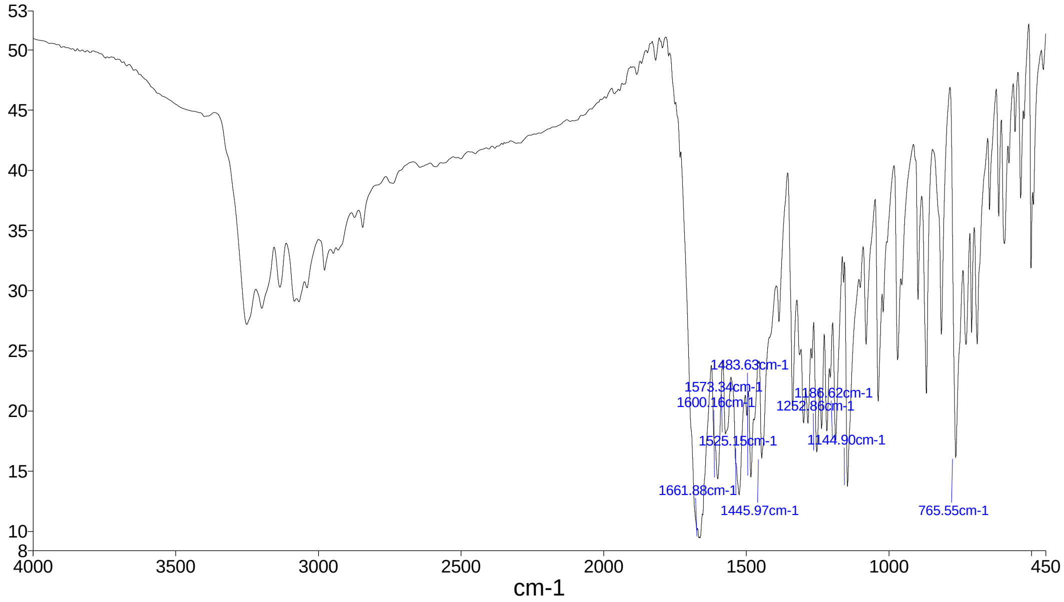
1483.63cm-1
1573.34cm-1
1600.16cm-1
1186.62cm-1
1252.86cm-1
1525.15cm-1
1144.90cm-1
1661.88cm-1
1445.97cm-1
765.55cm-1
cm-1

Fig. S14. *3-(1H-benzo[d]imidazol-2-yl)-2-((4-methylbenzyl)thio)quinolone (**6n**)*

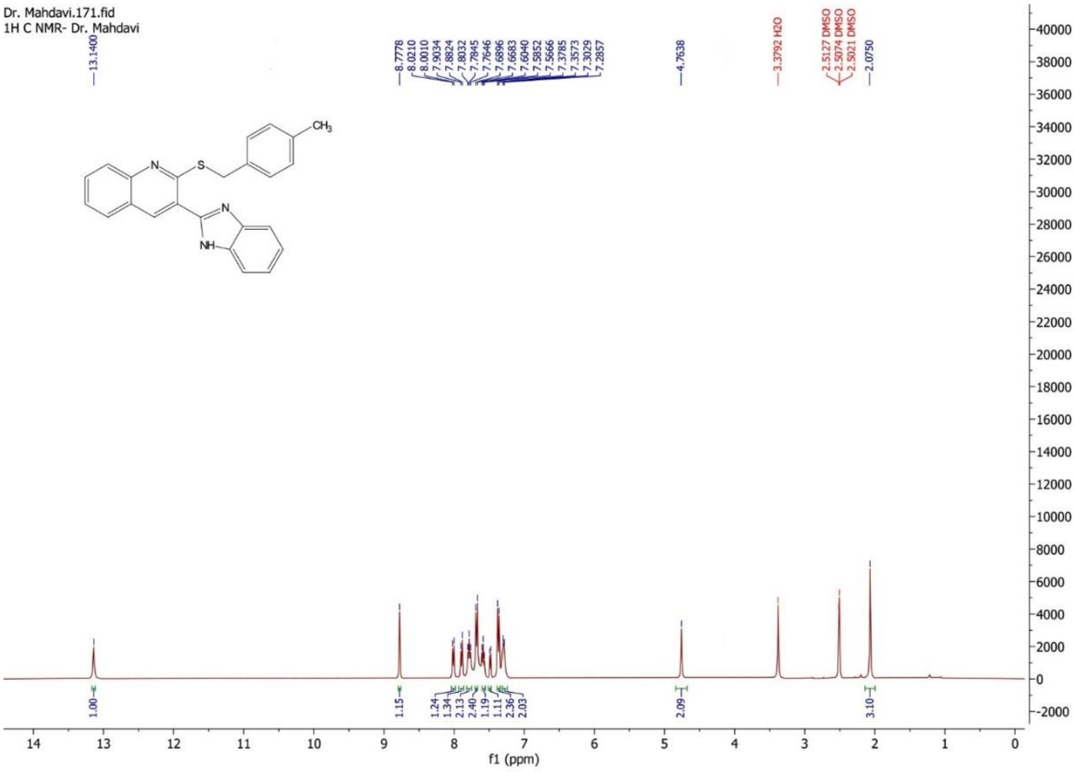

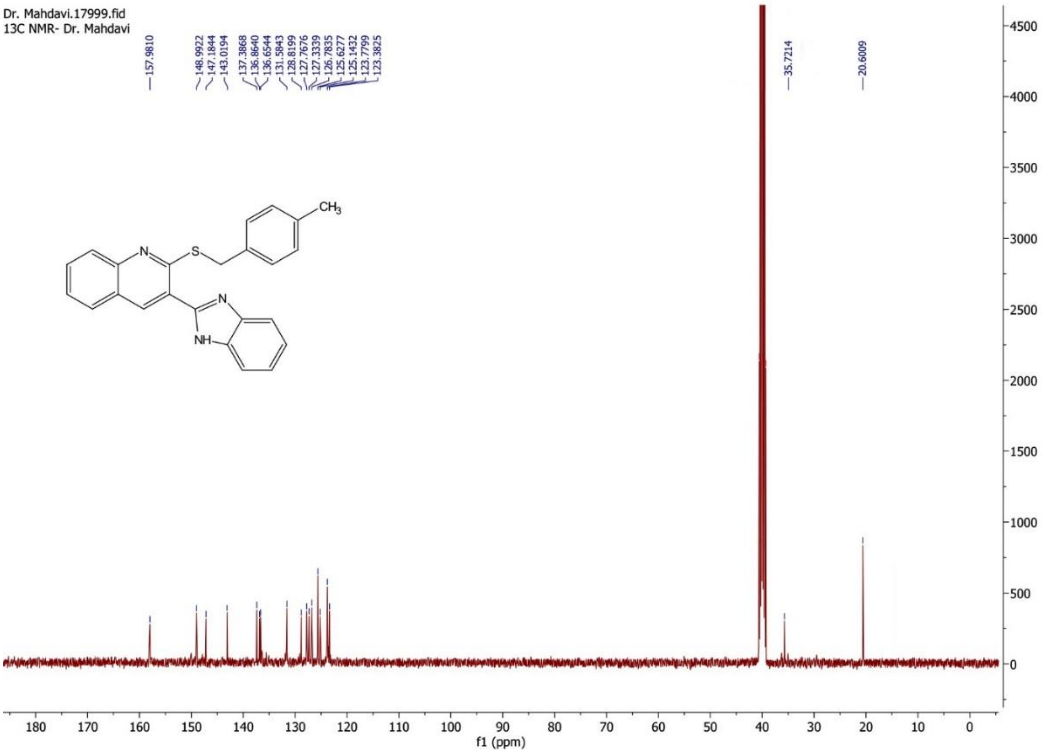

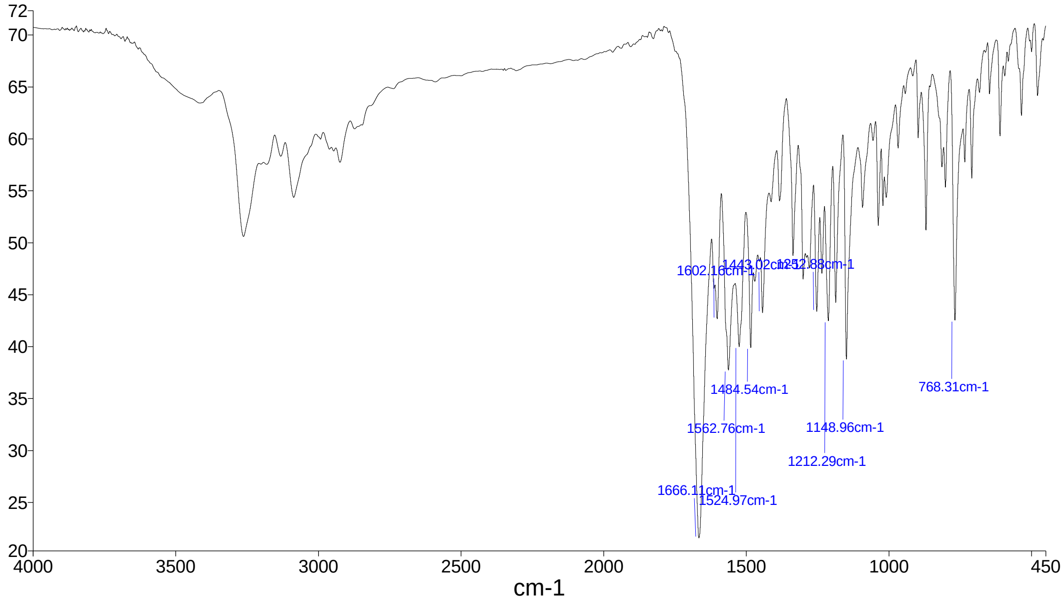

72
70
65
60
55
50
45
40
35
30
25
20
4000
3500
3000
2500
2000
1500
1000
450
cm-1
1602.16cm-1
1443.02cm-1
1252.88cm-1
1484.54cm-1
1562.76cm-1
1148.96cm-1
1212.29cm-1
1666.11cm-1
1524.97cm-1
768.31cm-1

Fig. S15. *3-(1H-benzo[d]imidazol-2-yl)-2-((2,3dimethylbenzyl)thio)quinolone (**6o**)*

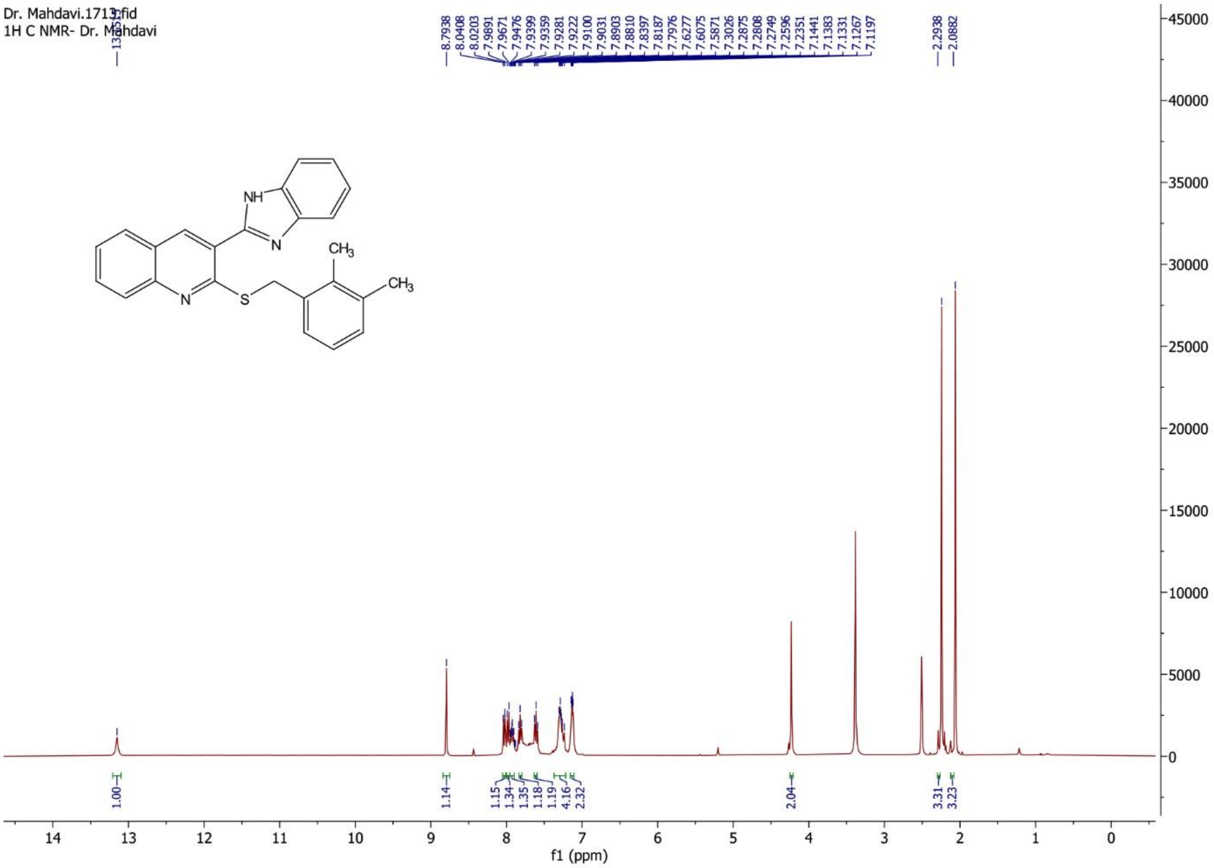

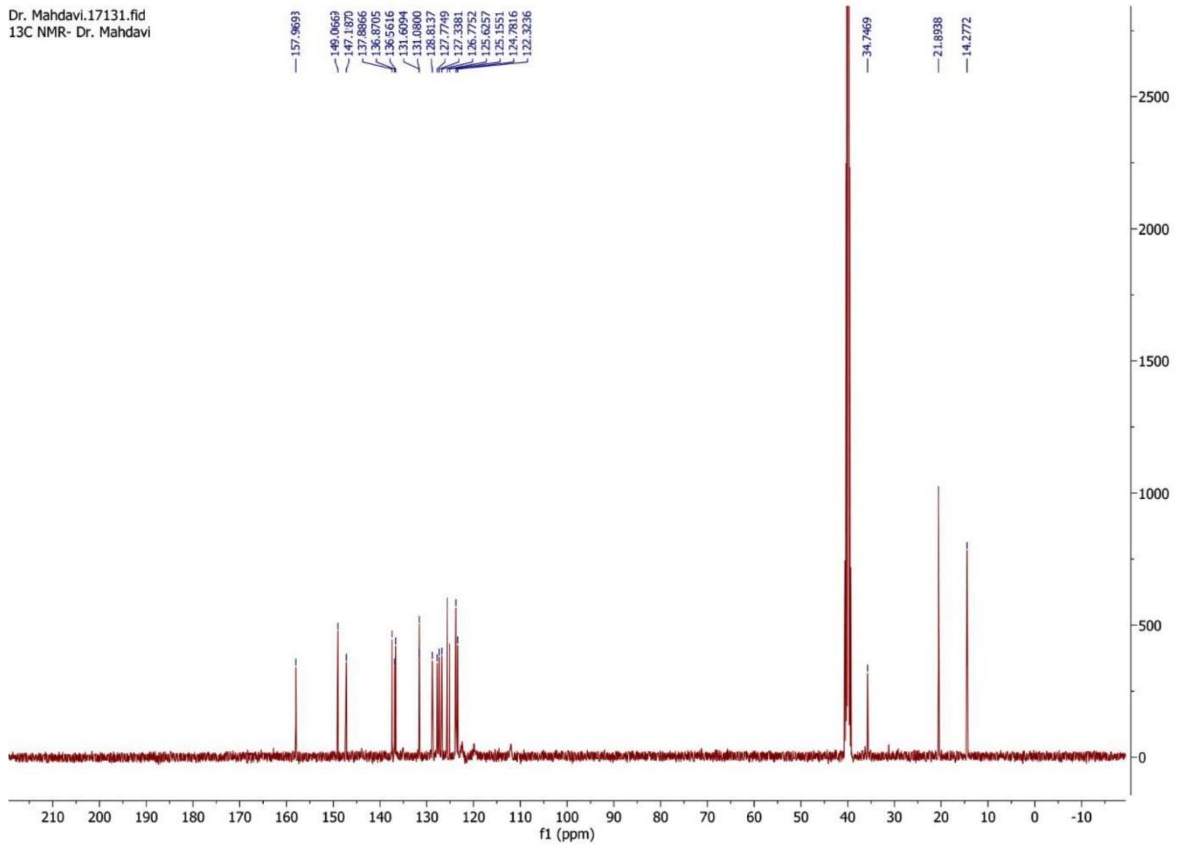
Dr. Mahdavi.17131.fid
13C NMR- Dr. Mahdavi
157.9693
149.0669
147.1870
137.8866
136.8705
136.5616
131.6094
131.0800
128.8137
127.7749
127.3381
126.7752
125.6257
125.1551
124.7816
122.3236
34.7469
21.8938
14.2772
f1 (ppm)

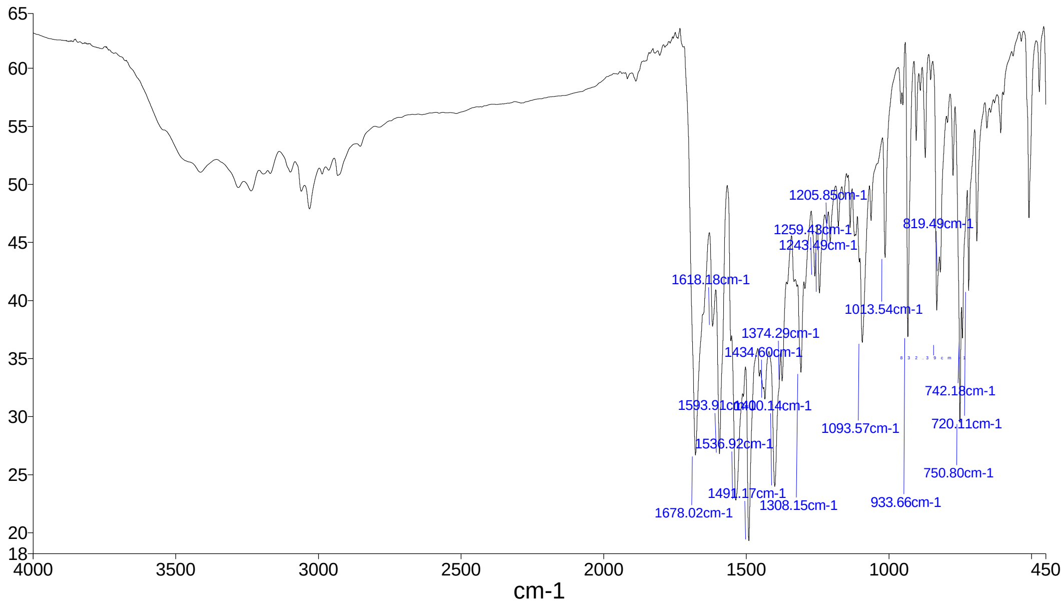
1678.02cm-1
1618.18cm-1
1593.91cm-1
1536.92cm-1
1491.17cm-1
1434.60cm-1
1400.14cm-1
1374.29cm-1
1308.15cm-1
1259.43cm-1
1243.49cm-1
1205.85cm-1
1093.57cm-1
1013.54cm-1
933.66cm-1
819.49cm-1
750.80cm-1
742.18cm-1
720.11cm-1
cm-1

Fig. S16. *3-(1H-benzo[d]imidazol-2-yl)-2-((4-nitrobenzyl)thio)quinolone (**6p**)*

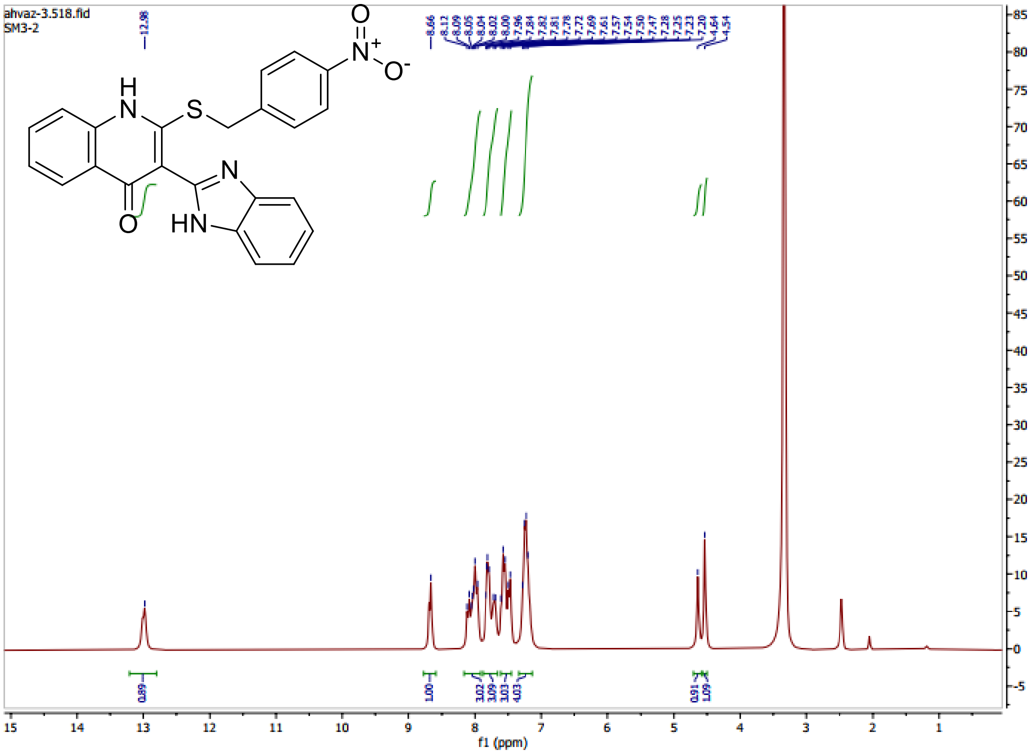

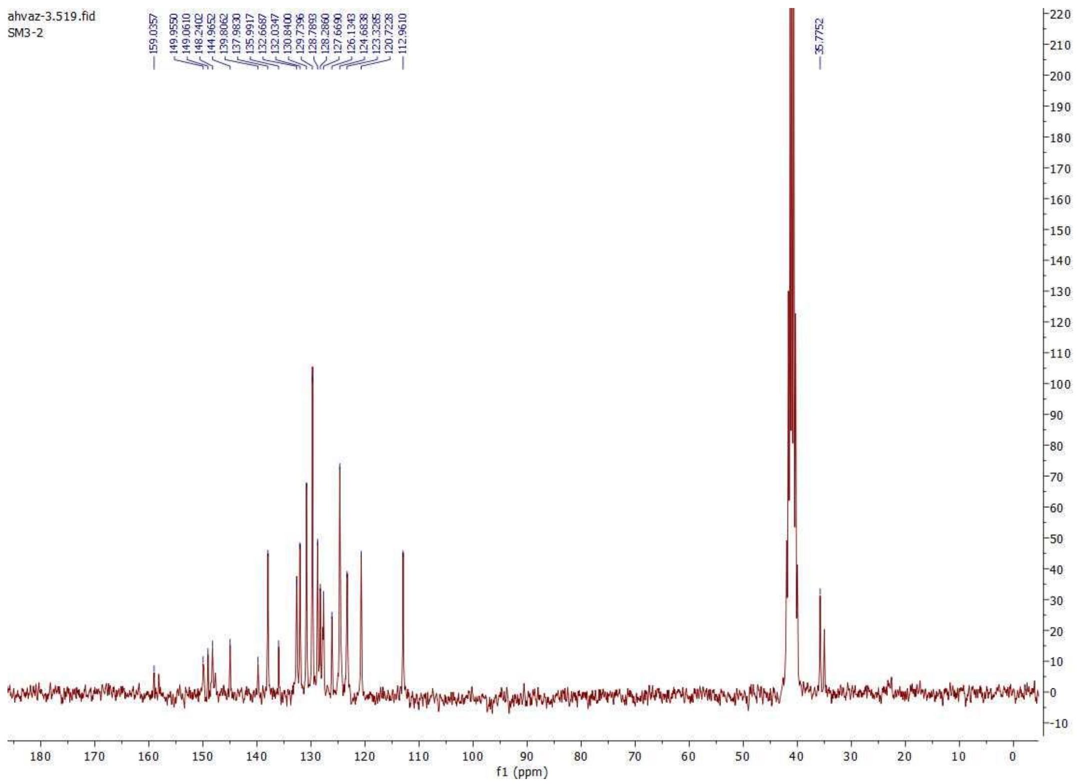
ahvaz-3.519.fid
SM3-2
159.0357
149.9550
149.0610
148.2402
144.9652
139.8062
137.9830
135.9917
132.6687
132.0347
130.8400
129.7396
128.7893
128.2860
127.6690
126.1343
124.6838
123.3285
120.7228
112.9610
35.7752
f1 (ppm)

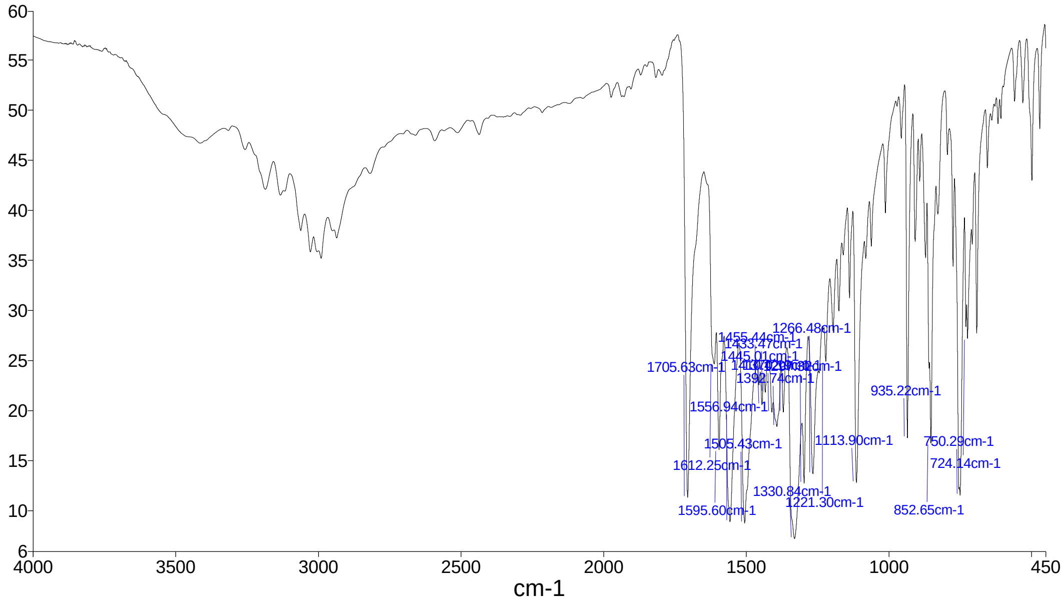
1266.48cm-1
1455.44cm-1
1433.47cm-1
1445.01cm-1
1705.63cm-1
1392.74cm-1
935.22cm-1
1556.94cm-1
1505.43cm-1
1113.90cm-1
750.29cm-1
724.14cm-1
1612.25cm-1
1330.84cm-1
1221.30cm-1
1595.60cm-1
852.65cm-1
cm-1

Fig. S17. *3-(1H-benzo[d]imidazol-2-yl)-2-((4-methoxybenzyl)thio)quinolone (**6q**)*

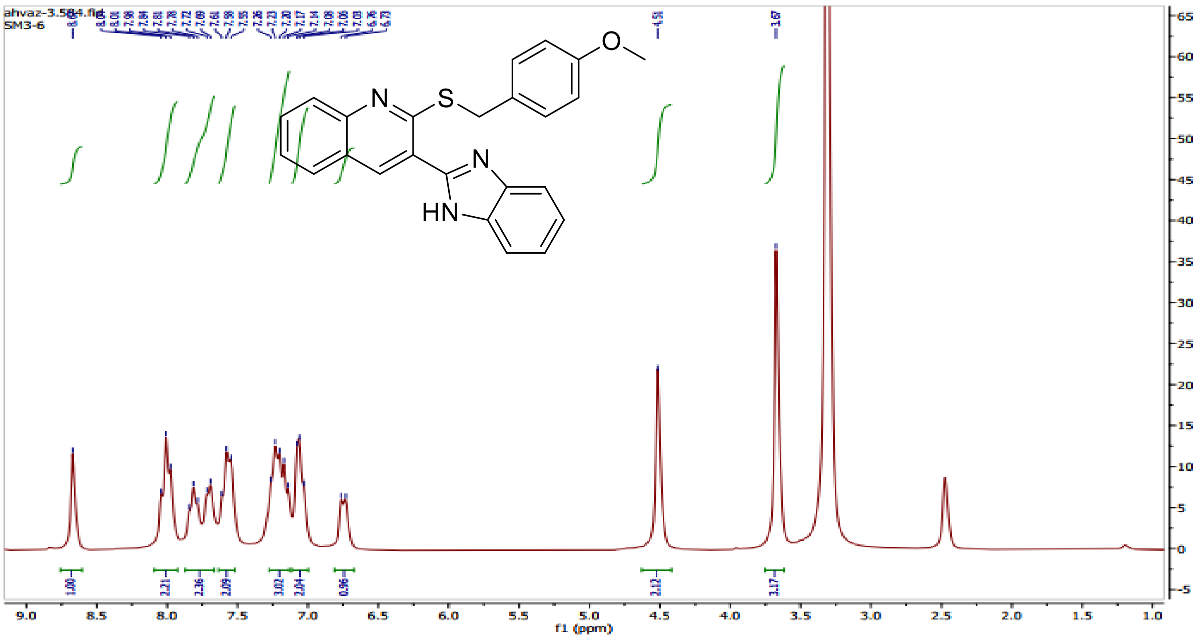

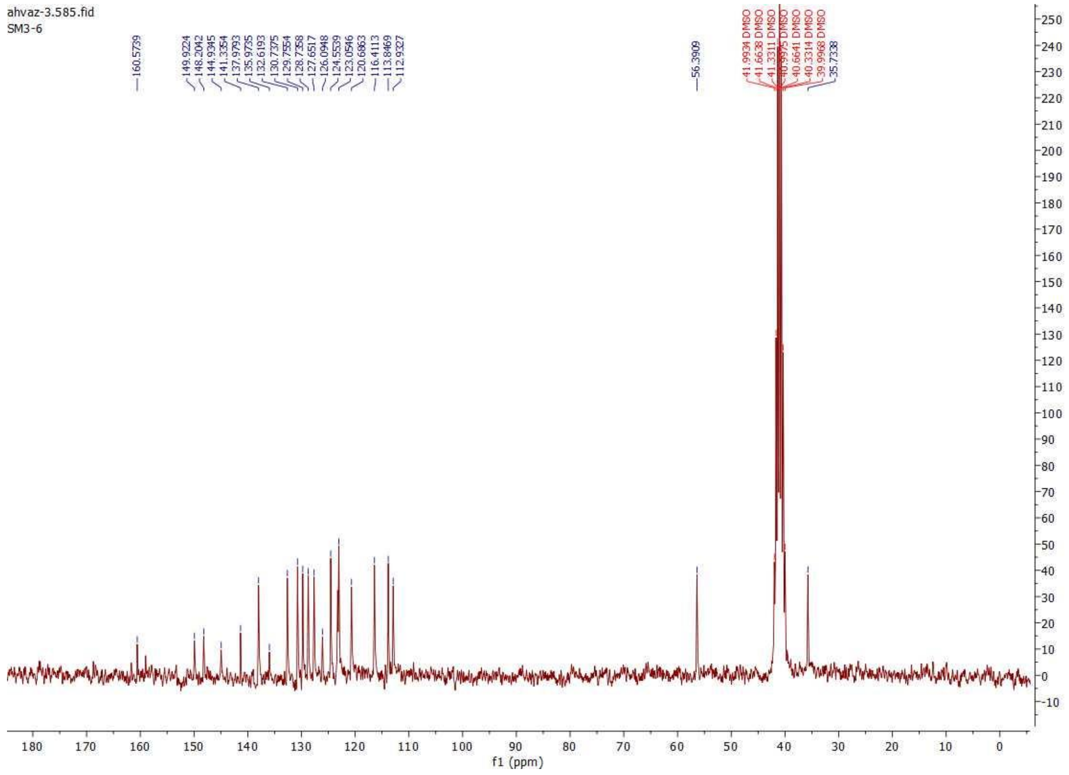
ahvaz-3.585.fid
SM3-6
160.5739
149.9224
148.2042
144.9345
141.3354
137.9793
135.9735
132.6193
130.7375
129.7554
128.7358
127.6517
126.0948
124.5539
123.0546
120.6863
116.4113
113.8469
112.9327
56.3909
41.9934 DMSO
41.6638 DMSO
41.3311 DMSO
40.9975 DMSO
40.6641 DMSO
40.3314 DMSO
39.9968 DMSO
35.7338
f1 (ppm)

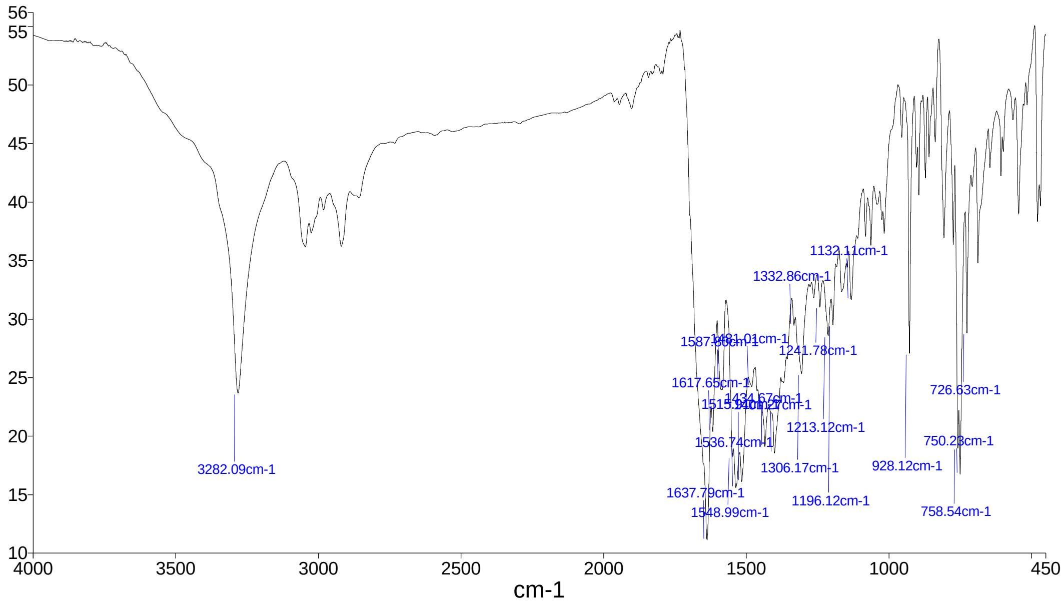
3282.09cm-1
1637.79cm-1
1617.65cm-1
1548.99cm-1
1536.74cm-1
1434.67cm-1
1332.86cm-1
1306.17cm-1
1241.78cm-1
1213.12cm-1
1196.12cm-1
1132.11cm-1
928.12cm-1
758.54cm-1
750.23cm-1
726.63cm-1
cm-1

Fig. S18. *3-(1H-benzo[d]imidazol-2-yl)-2-(ethylthio)quinolone* (***6r***)

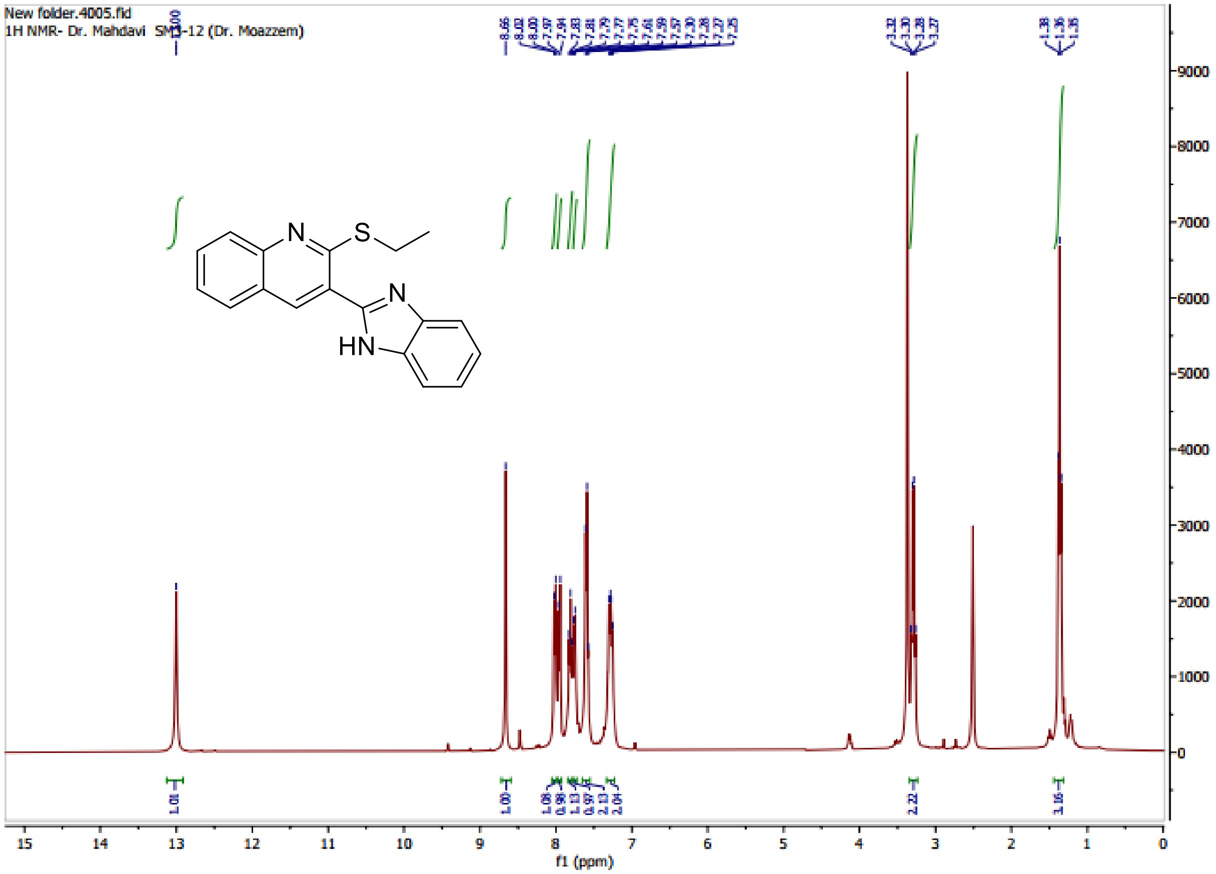

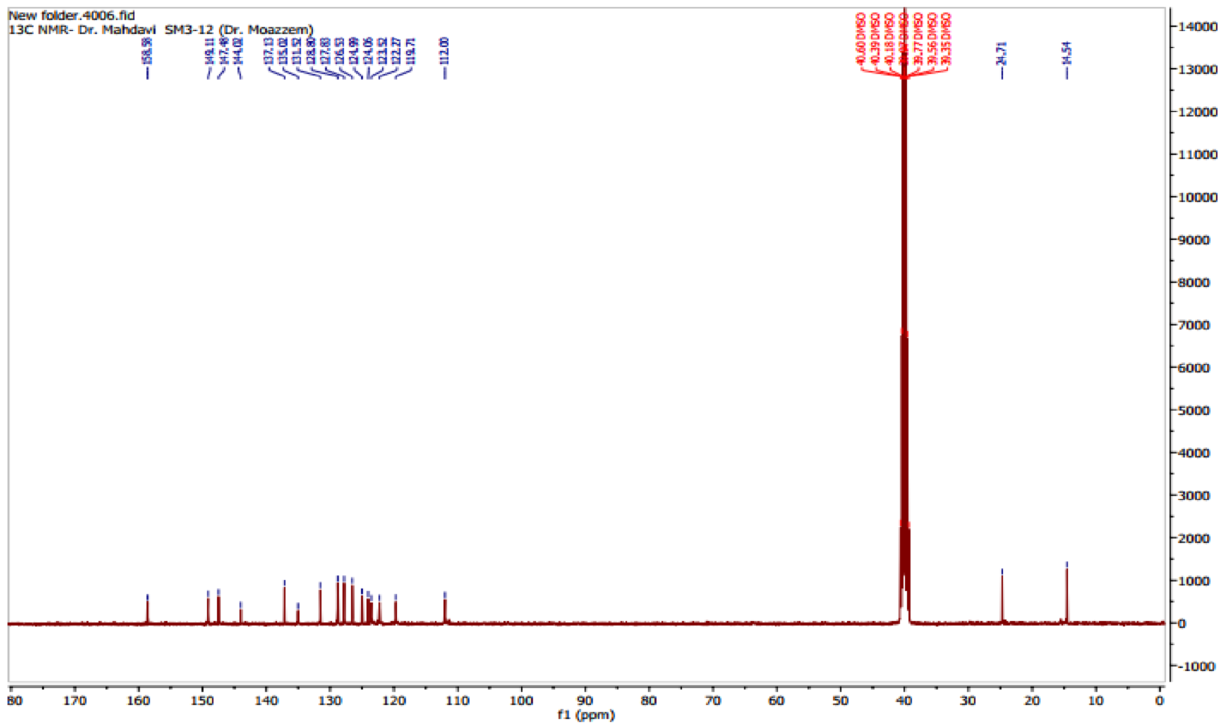
New folder.4006.fid
13C NMR- Dr. Mahdavi SM3-12 (Dr. Moazzem)
158.58
149.11
147.48
144.02
137.13
135.02
131.52
128.80
127.83
126.53
124.99
124.06
123.52
122.77
119.71
112.00
40.60 DMSO
40.39 DMSO
40.18 DMSO
39.77 DMSO
39.56 DMSO
39.35 DMSO
24.71
14.54
f1 (ppm)

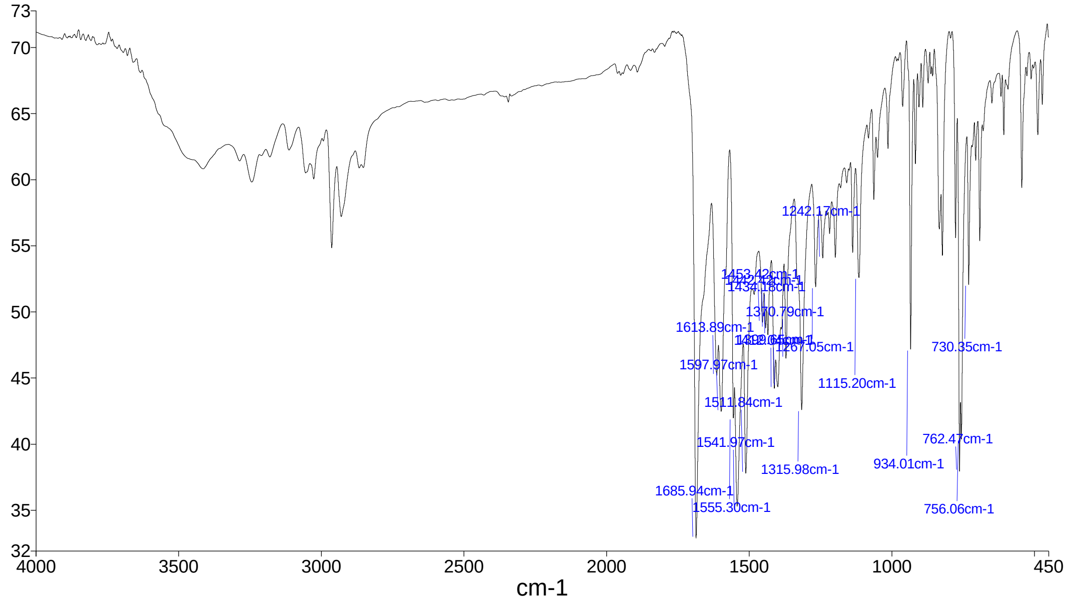
1242.17cm-1
1453.42cm-1
1434.18cm-1
1370.79cm-1
1613.89cm-1
1267.05cm-1
1597.97cm-1
1115.20cm-1
730.35cm-1
1511.84cm-1
1541.97cm-1
762.47cm-1
1315.98cm-1
934.01cm-1
1685.94cm-1
1555.30cm-1
756.06cm-1
cm-1
